# Host-directed clearance of nuclear tegument U14 terminates NF-κB signaling and supports HHV-6A replication

**DOI:** 10.1371/journal.ppat.1014576

**Published:** 2026-09-23

**Authors:** Khoir Amaliin, Mansaku Hirai, Salma Aktar, Tetsuo Koshizuka, Yasuko Mori, Jun Arii

**Affiliations:** 1 Department of Virology, Graduate School of Biomedical and Health Sciences, Hiroshima University, Hiroshima, Japan; 2 Division of Clinical Virology, Center for Infectious Diseases, Kobe University Graduate School of Medicine, Kobe, Hyogo, Japan; 3 Department of Microbiology and Immunology, Gifu Pharmaceutical University, Gifu, Japan; Emory Vaccine Center, UNITED STATES OF AMERICA

## Abstract

Innate immune and cellular stress responses triggered by infection must be transient and tightly controlled to restrain pathogens without harming the host. Herpesvirus infection induces NF-κB signaling through multiple viral and host inputs, yet how this response is terminated during productive infection remains unclear. Here, we identify a host-regulated timing mechanism that limits infection-associated NF-κB signaling during human herpesvirus 6A (HHV-6A) infection. The HHV-6A tegument protein U14 has the intrinsic capacity to induce NF-κB signaling and contributes to infection-associated NF-κB responses. The nuclear adaptor PDLIM2 associates with U14 and couples proteasome-dependent U14 turnover to phospho-p65 (Ser536) downregulation and NF-κB termination. CRISPRi-mediated PDLIM2 knockdown prolonged phospho-p65 (Ser536) signaling and nuclear U14 accumulation, enhanced antiviral and DNA damage-associated programs, increased γH2AX-marked nuclear stress, and reduced extracellular HHV-6A genome copies. Conversely, U14 knockdown attenuated viral gene expression, extracellular viral genome accumulation, and NF-κB-linked inflammatory gene induction. Collectively, these findings define the U14–PDLIM2 axis as a host-regulated mechanism that coordinates NF-κB termination with control of nuclear stress to support productive HHV-6A infection.

## Author summary

Herpesviruses package viral proteins within the virus particle and deliver them into cells at the moment of entry. Some of these proteins rapidly reach the nucleus and can contribute to NF-κB–dependent host responses. These early signals can help initiate infection, but they must be shut off before they become harmful to the infected cell. In this study, we asked how infection-associated nuclear signals are switched off at the right time. We found that, during human herpesvirus 6A (HHV-6A) infection, the viral tegument protein U14 has the capacity to induce NF-κB signaling and contributes to NF-κB–linked responses. We also found that the host nuclear adaptor PDLIM2 associates with U14 and helps terminate this signal by promoting phospho-p65 (Ser536) downregulation and U14 turnover through a proteasome-dependent process. This timed regulation limits ATM-linked nuclear stress and supports viral gene expression and extracellular viral genome accumulation. Upon PDLIM2 knockdown, antiviral and DNA damage-associated programs increase, cellular fitness declines, and extracellular viral genome copies decrease. These findings reveal a host-regulated timing mechanism that coordinates NF-κB shutdown with U14 turnover to support productive HHV-6A infection.

## Introduction

Potent innate immune and cellular stress signals triggered by infection often must be transient to preserve cellular survival and fitness: they are essential to restrain pathogens and restore homeostasis, yet they become harmful if sustained over time [1–3]. Herpesviruses deliver preformed, virion-borne tegument proteins (proteins positioned between the capsid and envelope) into infected cells immediately after entry, and some of these proteins rapidly reach the nucleus, where they acutely engage host pathways [4]. However, it remains unclear how infection-associated nuclear signals are terminated and associated nuclear stress is resolved. We therefore propose a “trigger–timer–terminator” logic for herpesvirus infection, in which multiple entry- and infection-associated inputs initiate nuclear signaling; host regulatory mechanisms shape its amplitude and duration; and proteasome-dependent turnover of contributing nuclear tegument proteins helps terminate the response.

NF-κB has phase- and context-dependent roles during herpesvirus infection. Transient canonical NF-κB signaling, commonly mediated by p50/p65 complexes and regulated by IκBα, can support viral gene expression and infected-cell survival. Conversely, NF-κB activation can also stimulate type I interferon and inflammatory cytokine responses and, when excessive or inadequately controlled, contribute to cellular stress and immunopathology. Thus, the net effect of NF-κB depends on the viral species, cell type, and stage of infection [5–9]. Within this framework, PDLIM2 is a compelling timer–terminator candidate because it constrains NF-κB signaling by facilitating ubiquitin-dependent proteasomal degradation of activated NF-κB p65 (RelA) in the nucleus [10]. Human herpesvirus 6A (HHV-6A; genus *Roseolovirus*, subfamily *Betaherpesvirinae*) provides a tractable context to test this logic because it undergoes multi-day replication cycles in which unresolved nuclear stress could impose late-phase costs. HHV-6A delivers the tegument protein U14 at entry; previous work showed that U14 associates with p65 and promotes NF-κB-dependent transcription. Moreover, pharmacological inhibition of NF-κB reduces HHV-6A protein and viral genome accumulation, supporting a positive contribution of controlled NF-κB activity to productive infection [8]. These observations raise the question of how U14-linked NF-κB signaling is terminated before prolonged activation becomes detrimental.

Here, we show that PDLIM2 links termination of a U14-linked NF-κB pulse to U14 turnover during HHV-6A infection. The ubiquitin adaptor PDLIM2 associates with HHV-6A U14 and promotes proteasome-dependent U14 turnover together with phospho-p65 (Ser536) downregulation; PDLIM2 expression was also associated with enhanced ubiquitin modification of U14. CRISPRi-mediated PDLIM2 knockdown was associated with prolonged phospho-p65 (Ser536) signaling and nuclear U14 accumulation, enhanced antiviral and DNA damage-associated programs, compromised cellular fitness during infection, and reduced extracellular HHV-6A genome copies. Conversely, late-phase ATM inhibition increased extracellular viral genome copy numbers, supporting a restrictive role for ATM-linked DNA damage signaling during HHV-6A infection. Taken together, these findings define the U14–PDLIM2 axis as a host-regulated timing mechanism that coordinates NF-κB termination with control of nuclear stress during HHV-6A infection.

## Results

### HHV-6A infection elicits transient NF-κB activation, while U14-linked signaling undergoes active termination

**Fig 1:**
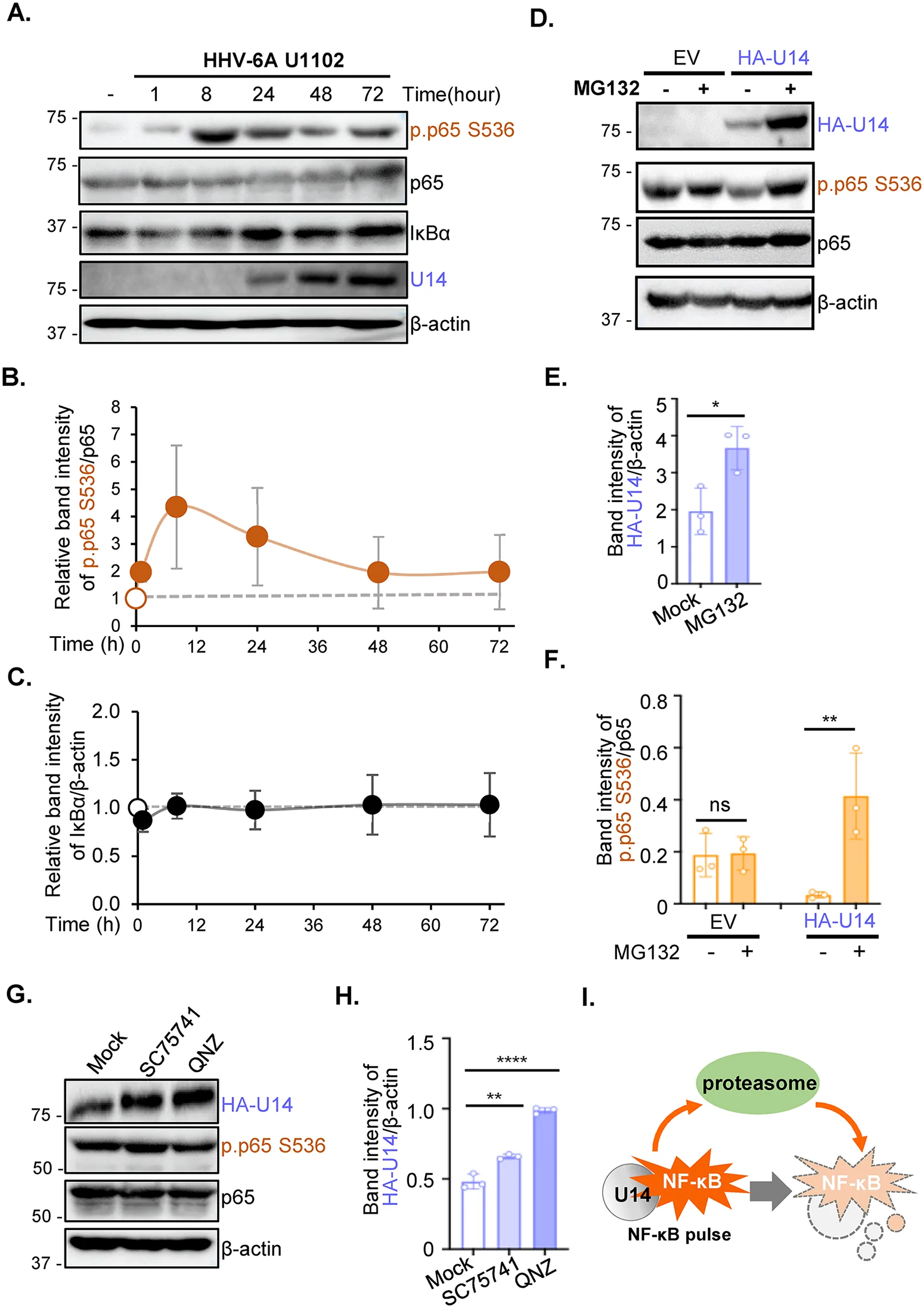
HHV-6A infection elicits transient NF-κB activation followed by a proteasome-dependent decline. (A) Immunoblots for phospho-p65 (Ser536), total p65, IκBα, U14, and β-actin at the indicated hours post-infection (hpi) of HHV-6A-infected JJhan T cell line. Representative of three independent experiments. (B, C) Densitometric quantification of phospho-p65 (Ser536) normalized to total p65 (B) and IκBα normalized to β-actin (C) over time. Data are means ± SD (n = 3). (D) HEK293T cells were transfected with HA–HHV-6A U14 or empty vector (EV) for 24 h and treated with MG132 (2.5 μM) or vehicle for the final 4 h before harvest; immunoblot for HA–U14, phospho-p65 (Ser536), total p65, and β-actin to assess proteasome dependence of the post-peak decline. (E, F) Densitometric quantification of HA–HHV-6A U14 normalized to β-actin (E) and phospho-p65 (Ser536) normalized to p65 (F) from (D). Data are means ± SD (n = 3). Two-tailed Student’s t-test: *, p < 0.05; **, p < 0.01; ns, not significant. (G) HEK293T cells were transfected with HA–HHV-6A U14 for 24 h and treated with the NF-κB inhibitor SC75741 (200 nM) or QNZ (10 nM) or vehicle for 30 min before harvest; immunoblot for HA–U14, phospho-p65 (Ser536), total p65, and β-actin to test whether NF-κB pathway activity contributes to U14 turnover. (H) Densitometric quantification of HA–HHV-6A U14 normalized to β-actin from (G). Bar graphs show means ± SD (n = 3). One-way ANOVA with Tukey’s multiple-comparison test: **, p < 0.01; ****, p < 0.0001. (I) Schematic model showing that U14-linked NF-κB signaling is coupled to proteasome-dependent downregulation of phospho-p65 (Ser536), thereby terminating the pulse.

Guided by the trigger–timer–terminator framework, we first quantified NF-κB signaling dynamics during HHV-6A infection using phosphorylated p65 (RelA) as a time-resolved sentinel. A time-course analysis revealed an NF-κB activation peaking at 8 hours post-infection (hpi) (4.35-fold, baseline = 1.0) followed by return toward baseline; the downslope was well fit by a single exponential with 𝜏_decay_ = 47.8 h (half-life 33.1 h), consistent with a linear-interpolation estimate of 34.9 h over 24–48 h (Fig 1A–1C). The transient decrease in IκBα observed at 1 hpi, followed by its recovery at 8 hpi, could be consistent with an early canonical NF-κB response initiated by viral glycoprotein–receptor engagement, as reported for other herpesviruses. However, the limited sampling during the earliest phase of infection does not allow us to distinguish this possibility from other virion-derived inputs. Because early NF-κB activation after herpesvirus entry can also be driven by tegument proteins [11–13], and because HHV-6A U14 is known to associate with p65 [8], we tested the intrinsic capacity of U14 to activate NF-κB in the absence of other viral proteins. Doxycycline-inducible expression of U14 produced a transient phospho-p65 (Ser536) pulse, supporting the capacity of U14 to induce NF-κB signaling in isolation (S1 Fig). Thus, receptor-mediated signaling and U14-mediated activation are not mutually exclusive and may both contribute to NF-κB activation during the early phase of HHV-6A infection. Notably, despite the transient early decrease, IκBα levels were not persistently increased during the subsequent decline in phospho-p65 (Ser536) in either HHV-6A-infected cells or HHV-6A U14-expressing cells (Fig 1A and S1A Fig), suggesting that the decline in phospho-p65 (Ser536) is unlikely to result from IκB upregulation. As shown in S1B–S1D Fig, doxycycline-inducible expression of the mScarlet-1 control or HHV-6A U29, a viral capsid protein, did not detectably increase phospho-p65 (Ser536), supporting the specificity of the U14 effect on NF-κB activation.

To distinguish active termination from passive decay, we perturbed proteasome-dependent turnover during the decline phase in the U14 expression system. Brief proteasome blockade with MG132 during this post-peak interval increased both phospho-p65 (Ser536) and U14 abundance and attenuated their decline (Fig 1D–1F), indicating that the post-peak fall depends on proteasome activity. Quantitatively, MG132 increased the phospho-p65 (Ser536) signal by 12.2-fold in U14-expressing cells but had little effect in empty-vector controls (1.04-fold). Under MG132, phospho-p65 (Ser536) remained 2.13-fold higher in U14-expressing cells than in empty-vector cells, along with accumulation of U14 expression, which remained 1.87-fold higher, consistent with a U14-driven signal that is curtailed by proteasome-dependent clearance. Conversely, pharmacologic inhibition of NF-κB with SC75741, which impairs DNA binding by the NF-κB subunit p65 [14,15], or QNZ (EVP4593), an NF-κB transcriptional blocker that acts through inhibition of store-operated calcium entry [16,17], attenuated U14 turnover (Fig 1G, 1H) without affecting cell viability under these conditions (S2A Fig). Together, these results are consistent with a model in which U14-linked NF-κB signaling is coupled to proteasome-dependent downregulation of phospho-p65 (Ser536), thereby terminating the pulse (Fig 1I). We next investigated the mechanism underlying this proteasome-dependent termination and U14 turnover.

### PDLIM2 couples NF-κB termination to U14 turnover

**Fig 2:**
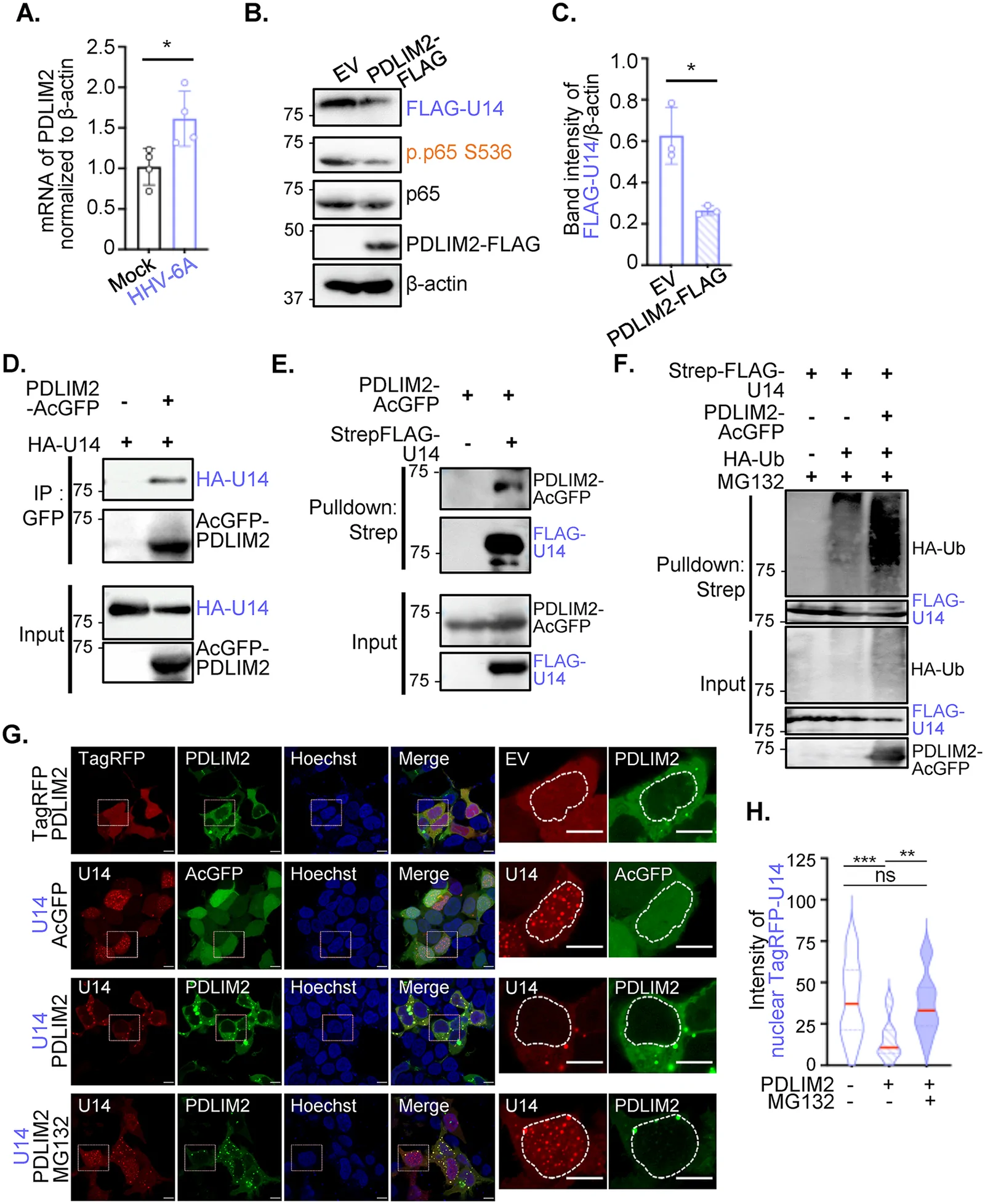
PDLIM2 links NF-κB termination to enhanced ubiquitin modification and turnover of U14. (A) RT-qPCR of PDLIM2 mRNA in JJhan cells that were mock-infected or infected with HHV-6A U1102 for 72 h. PDLIM2 mRNA levels were normalized to β-actin. Bar graphs show means ± SD (n = 3). Two-tailed Student’s t-test: *, p < 0.05. (B) Immunoblot of HEK293T cells transfected to express Strep–FLAG–HHV-6A U14 and PDLIM2–FLAG or empty vector (EV), probed with the indicated antibodies. Representative of three independent experiments. (C) Densitometric quantification of FLAG–U14 normalized to β-actin from (B). Data are means ± SD (n = 3). Two-tailed Student’s t-test: *, p < 0.05. (D) HEK293T cells expressing HA–HHV-6A U14 with PDLIM2–AcGFP or EV for 48 h were lysed and subjected to immunoprecipitation with anti-GFP antibody, followed by immunoblotting for the indicated proteins. (E) HEK293T cells expressing PDLIM2–AcGFP with Strep–FLAG–HHV-6A U14 or EV for 48 h were lysed and subjected to Strep-Tactin pull-down, followed by immunoblotting for the indicated proteins. (F) HEK293T cells expressing Strep–FLAG–HHV-6A U14 together with HA–ubiquitin (HA–Ub) and/or PDLIM2–AcGFP for 18 h were treated with MG132 (20 μM) for the final 6 h. Strep-Tactin affinity purifications were analyzed by immunoblotting for PDLIM2–AcGFP, FLAG–U14, and HA–ubiquitin. (G) Representative images of HEK293T cells co-expressing TagRFP–HHV-6A U14 with PDLIM2–AcGFP or the corresponding EVs for 24 h, with or without MG132. Rightmost panels show magnified views of the regions indicated by white dashed boxes in the corresponding overview images. In the magnified views, nuclear contours were traced from the Hoechst 33342 channel and are delineated with white dotted lines. Scale bars, 10 μm. (H) Quantification of nuclear TagRFP–HHV-6A U14 intensity under the indicated conditions. Single-cell measurements from one representative experiment (n = 18 cells per condition). Red horizontal lines indicate the median; diagonal hatch marks indicate the first and third quartiles. Group differences were evaluated with Tukey’s multiple-comparisons test. Independent experiments were repeated three times with similar results. Significance: **, p < 0.01; ***, p < 0.001; ns, not significant.

Ubiquitination followed by proteasomal degradation is a canonical means to terminate innate signaling [18,19]. Because PDLIM2 is a nuclear ubiquitin adaptor that constrains NF-κB signaling by targeting activated p65 for proteasome-dependent removal [10,20], we examined whether PDLIM2 could function as a timer–terminator in this setting. PDLIM2 mRNA levels were significantly higher in HHV-6A-infected cells than in mock-infected cells (Fig 2A). Co-expression of PDLIM2–FLAG with Strep–FLAG–HHV-6A U14 in HEK293T cells reduced phospho-p65 (Ser536) and concomitantly decreased U14 protein abundance (Fig 2B, 2C). Strep-Tactin pull-down of Strep–FLAG–HHV-6A U14 and reciprocal immunoprecipitation of PDLIM2–AcGFP with an anti-GFP antibody showed an association between HHV-6A U14 and PDLIM2 (Fig 2D, 2E). Strep affinity purification of Strep–FLAG–HHV-6A U14 from cells co-expressing HA–ubiquitin, followed by anti-HA immunoblotting, revealed increased anti-HA signal associated with affinity-purified U14 upon PDLIM2 expression (Fig 2F). This pattern is consistent with enhanced ubiquitin modification of U14 in the presence of PDLIM2, in line with its proteasome-dependent loss (Fig 1D, 1E). In cells, PDLIM2–AcGFP diminished nuclear TagRFP–HHV-6A U14 signals; this effect was reversed by proteasome inhibition with MG132 (Fig 2G, 2H). In agreement with Fig 1G and 1H, NF-κB pathway inhibition with SC75741 or QNZ also blunted nuclear U14 loss, suggesting reduced U14 turnover (S2B, S2C Fig). Together, these data support a model in which PDLIM2 links NF-κB termination to proteasome-dependent downregulation of phospho-p65 (Ser536) and turnover of HHV-6A U14.

**Fig 3:**
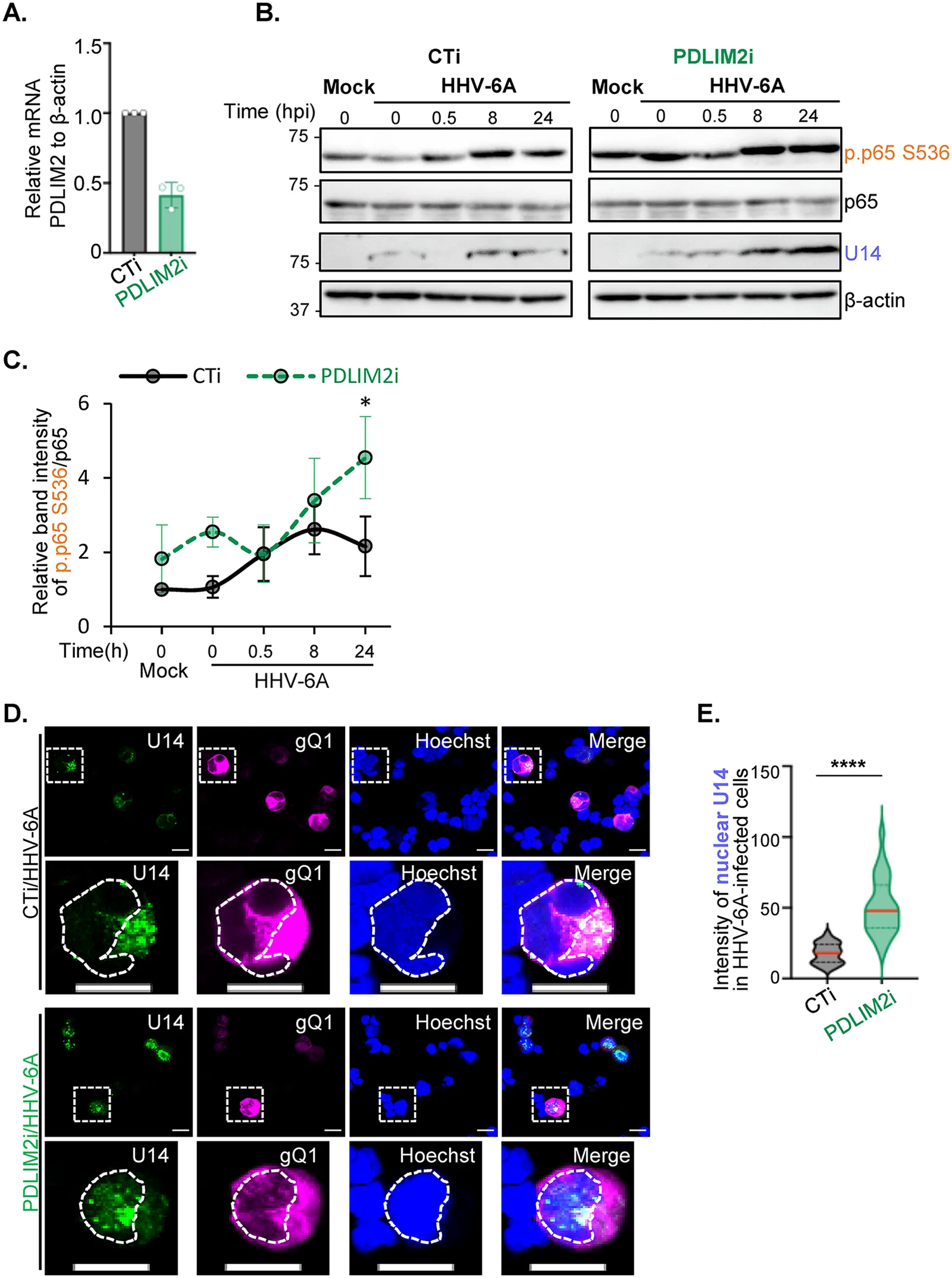
PDLIM2 limits NF-κB activation and nuclear U14 accumulation during HHV-6A infection. (A) PDLIM2 mRNA levels in JJhan control (CTi) and PDLIM2-knockdown (PDLIM2i) cells were quantified by RT-qPCR and normalized to β-actin to validate knockdown efficiency. Data are means ± SD (n = 3). (B) Representative immunoblots of phospho-p65 (Ser536), total p65, U14, and β-actin in JJhan-CTi and JJhan-PDLIM2i cells that were mock-infected or infected with HHV-6A and harvested at the indicated times. (C) Densitometric quantification of phospho-p65 (Ser536) relative to total p65 from (B). Data are presented as the means ± SD from three independent experiments. Statistical significance was assessed by two-way ANOVA followed by Šídák’s multiple-comparisons test. *, p < 0.05. (D) Representative immunofluorescence images of JJhan-CTi and JJhan-PDLIM2i cells infected with HHV-6A for 72 h, stained for U14 (green), gQ1 (magenta), and Hoechst 33342. For each condition, the panel immediately below shows a magnified view of the corresponding white dashed box; nuclear contours were traced from the Hoechst channel and are delineated with white dotted lines. Scale bars, 10 μm. (E) Quantification of nuclear HHV-6A U14 intensity in infected cells. Single-cell measurements from one representative experiment (n = 28 cells per condition); two-tailed unpaired Student’s t-test. Horizontal red lines indicate the median; diagonal hatch marks indicate the first and third quartiles. Significance: ****, p < 0.0001. Independent experiments were repeated three times with similar results.

We next used CRISPR interference-mediated PDLIM2 knockdown to examine the contribution of PDLIM2 to the U14-linked NF-κB response and U14 turnover during HHV-6A infection. These cells were generated using CRISPR interference targeting PDLIM2 (JJhan-PDLIM2i), alongside a no-guide control (JJhan-CTi) (Fig 3A). RT-qPCR confirmed reduced PDLIM2 mRNA expression in JJhan-PDLIM2i cells. Because endogenous PDLIM2 protein could not be reproducibly detected by immunoblotting under our experimental conditions, the extent of knockdown at the protein level was not directly assessed. Following infection, immunoblot analysis showed that HHV-6A induced a transient increase in phospho-p65 (Ser536) in JJhan-CTi cells, with a peak at 8 h followed by a decline at 24 h, consistent with the kinetics observed in wild-type JJhan cells infected with HHV-6A (Fig 1A, 1B). In contrast, phospho-p65 (Ser536) remained higher in JJhan-PDLIM2i cells throughout the time course and did not undergo the same post-peak decline, indicating that CRISPRi-mediated PDLIM2 knockdown prolongs the infection-induced NF-κB response (Fig 3B, 3C). The kinetics of phospho-p65 induction during infection likely reflect the integration of multiple positive and negative inputs, including virion-delivered U14, entry-associated signaling, viral gene expression, and host or viral negative regulators. Therefore, the infection-associated phospho-p65 waveform should not be interpreted as a direct kinetic equivalent of isolated U14 expression. Rather, these data support a model in which U14 contributes to infection-associated NF-κB-linked signaling, whereas PDLIM2 is involved in limiting the persistence of this response. Infected JJhan-PDLIM2i cells showed significantly increased nuclear U14 signals relative to JJhan-CTi cells at 72 h, consistent with reduced control of U14 accumulation under PDLIM2-knockdown conditions (Fig 3D, 3E).

**Fig 4:**
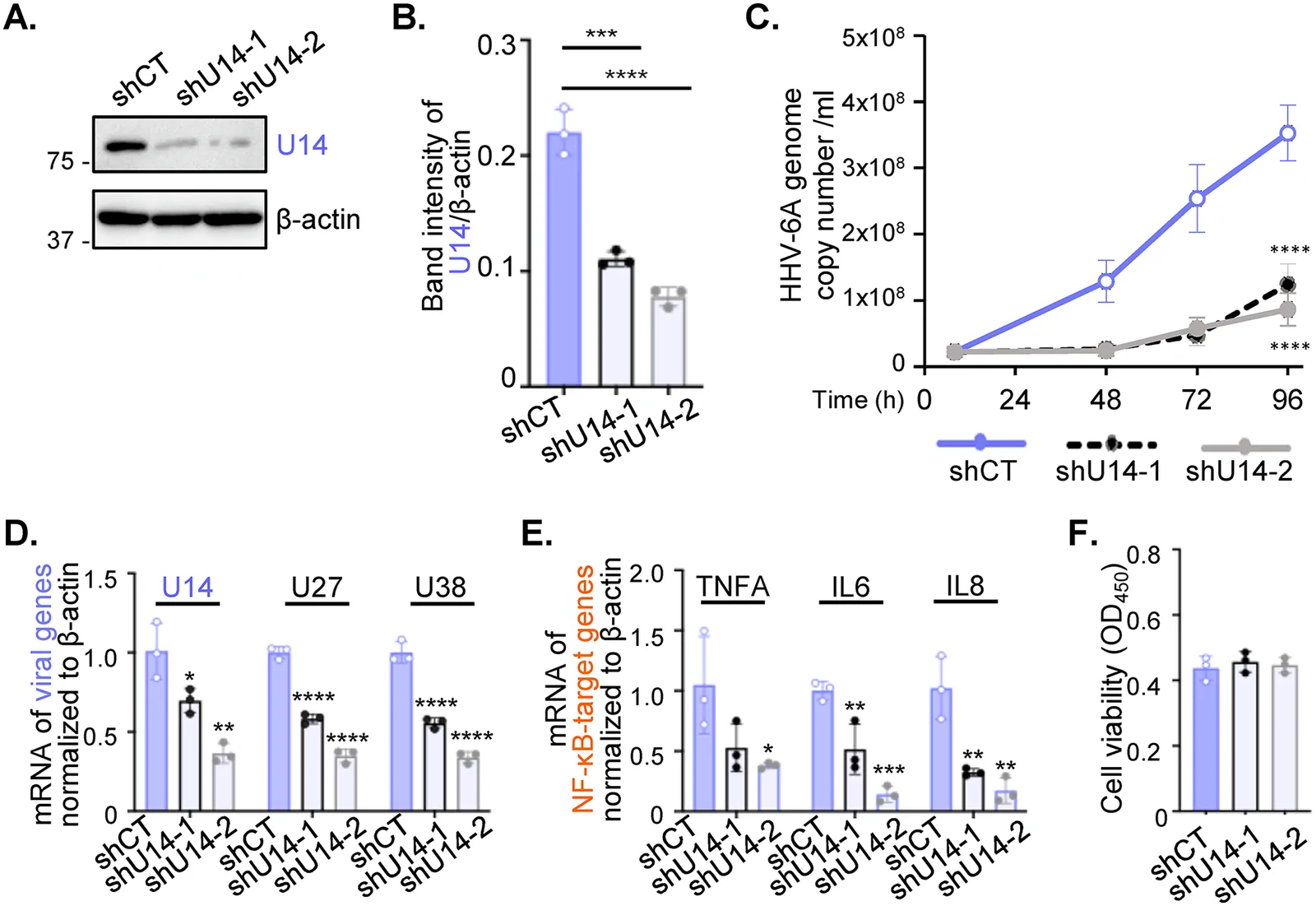
U14 supports HHV-6A amplification and proinflammatory transcription during infection. (A) Immunoblot analysis of U14 in HHV-6A U1102-infected JJhan cells expressing a control shRNA (shCT) or two independent U14-targeting shRNAs (shU14-1 and shU14-2) at 96 h post-infection. β-actin was used as a loading control. Representative of three independent experiments. (B) Densitometric quantification of U14 normalized to β-actin from panel A. Data are shown as means ± SD from three independent experiments. Statistical significance was determined by one-way ANOVA with Tukey’s multiple-comparison test: ***, p < 0.001; ****, p < 0.0001. (C) Viral genome copies released into culture supernatants from HHV-6A U1102-infected JJhan-shCT, JJhan-shU14-1, and JJhan-shU14-2 cells at the indicated times after infection. Statistical significance was determined by two-way ANOVA with Dunnett’s multiple-comparison test: ****, p < 0.0001. (D) RT-qPCR analysis of viral transcripts (U14, U27, and U38) in HHV-6A U1102-infected JJhan-shCT, JJhan-shU14-1, and JJhan-shU14-2 cells at 96 h post-infection. Transcript levels were normalized to β-actin. (E) RT-qPCR analysis of TNFA, IL6, and IL8 mRNA in HHV-6A U1102-infected JJhan-shCT, JJhan-shU14-1, and JJhan-shU14-2 cells at 96 h post-infection. Transcript levels were normalized to β-actin. (F) Cell viability measured by CCK-8 assay in uninfected JJhan-shCT, JJhan-shU14-1, and JJhan-shU14-2 cells. Absorbance was measured at 450 nm. Data are shown as means ± SD from three independent experiments. Statistical significance in (D–F) was determined by one-way ANOVA with Tukey’s multiple-comparison test: *, p < 0.05; **, p < 0.01; ***, p < 0.001; ****, p < 0.0001. In (F), no significant difference was detected.

Having found that CRISPRi-mediated PDLIM2 knockdown was associated with prolonged phospho-p65 signaling and increased nuclear U14 accumulation, we next asked whether U14 itself contributes to HHV-6A gene expression, extracellular viral genome accumulation and NF-κB-linked inflammatory gene induction. To this end, JJhan cells expressing a control shRNA or two independent U14-targeting shRNAs were infected with HHV-6A U1102. Both shRNAs targeted distinct sequences within the U14 coding region. Neither target sequence overlaps the reported mature miR-aU14 sequence [21] or an annotated adjacent protein-coding ORF. Immunoblotting confirmed efficient depletion of U14 (Fig 4A, 4B). Because U14 represents one contributor to infection-associated NF-κB signaling, this experiment was designed to assess the contribution of U14 to infection-associated signaling and viral replication-associated readouts, rather than p65 turnover during the termination phase. U14 knockdown significantly reduced the accumulation of viral genome copies in culture supernatants over time (Fig 4C), decreased the abundance of the viral transcripts U14, U27, and U38 (Fig 4D), and attenuated induction of the NF-κB-responsive inflammatory cytokine transcripts TNFA, IL6, and IL8 (Fig 4E), without significantly affecting cell viability (Fig 4F). These findings are consistent with our recent observation that U14 knockdown reduces phospho-p65 (Ser536) and viral gene expression during HHV-6B infection [22]. Together, these data indicate that U14 contributes to HHV-6A gene expression, extracellular viral genome accumulation, and NF-κB-linked inflammatory gene induction in JJhan cells.

### CRISPRi-mediated PDLIM2 knockdown enhances antiviral and DNA damage-associated programs during HHV-6A infection

**Fig 5:**
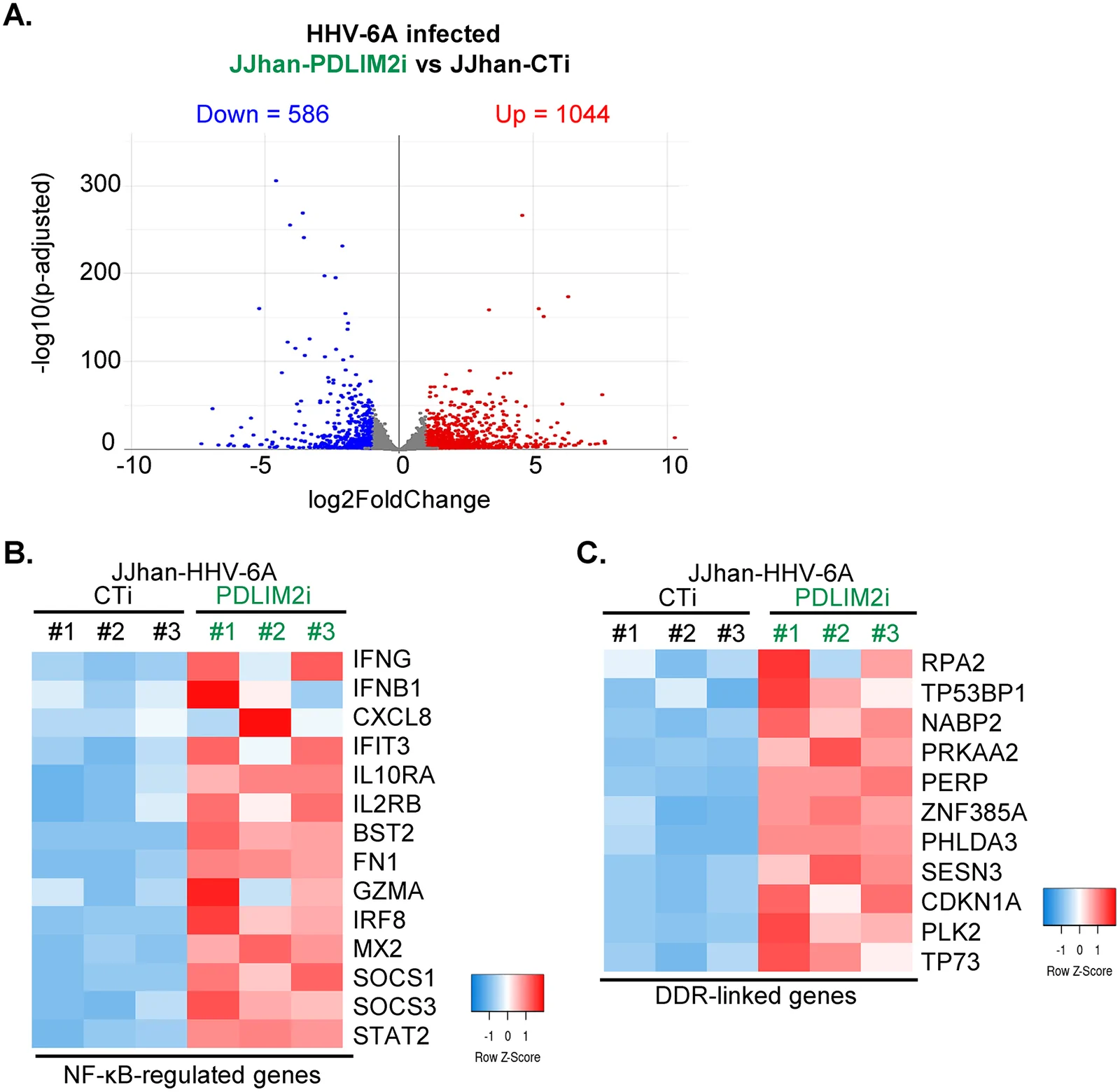
PDLIM2 knockdown is associated with increased NF-κB- and DDR-related gene expression in HHV-6A-infected JJhan cells. (A) Volcano plot showing differential gene expression between HHV-6A-infected JJhan-PDLIM2i and JJhan-CTi cells. Upregulated genes in PDLIM2i cells are shown in red, and downregulated genes are shown in blue. (B) Heatmap showing the expression of selected NF-κB-regulated genes in HHV-6A-infected JJhan-CTi and JJhan-PDLIM2i cells. (C) Heatmap showing the expression of selected DNA damage response (DDR)-linked genes in HHV-6A-infected JJhan-CTi and JJhan-PDLIM2i cells. For panels B and C, each column represents 1 biological replicate (n = 3 per group), and values are displayed as row Z-scores across samples.

**Fig 6:**
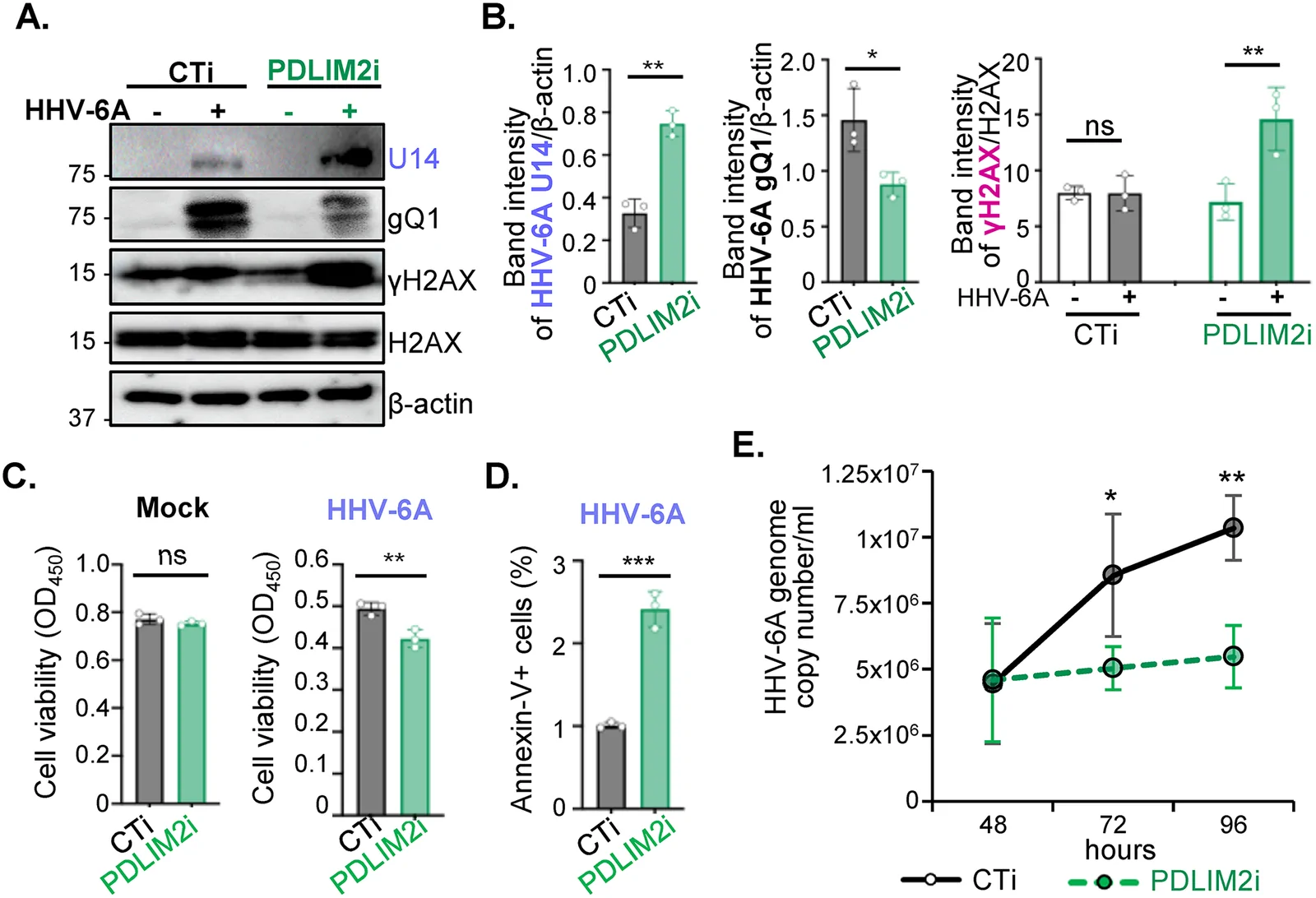
PDLIM2 knockdown increases U14 accumulation and DNA damage signaling while reducing extracellular HHV-6A genome copies. (A) Representative immunoblots of U14, gQ1, γH2AX, H2AX, and β-actin in CTi and PDLIM2i cells, either mock-infected or infected with HHV-6A for 72 h. (B) Densitometric quantification of HHV-6A U14 and gQ1 normalized to β-actin, and γH2AX normalized to total H2AX, from (A). Data are shown as means ± SD from three independent experiments. (C) Cell viability measured by CCK-8 assay in CTi and PDLIM2i cells under mock or HHV-6A-infected conditions at 72 h post-infection. Absorbance was measured at 450 nm. Data are shown as means ± SD from three independent experiments. (D) Percentage of Annexin V-positive cells in HHV-6A-infected CTi and PDLIM2i cells at 72 h post-infection. Data are shown as means ± SD from three independent experiments. (E) HHV-6A genome copy numbers released in culture supernatants from CTi and PDLIM2i cells at 48, 72, and 96 h after infection. Data are shown as means ± SD from three independent experiments. Two-tailed Student’s t-test: *, p < 0.05; **, p < 0.01.

Having found that PDLIM2 contributes to termination of the U14-linked NF-κB pulse, we next examined the downstream consequences in JJhan-PDLIM2i cells during HHV-6A infection. Comparative bulk RNA-seq of HHV-6A-infected JJhan-CTi and JJhan-PDLIM2i cells revealed upregulation of NF-κB-dependent and interferon-stimulated programs, enrichment of inflammatory pathways, and increased DNA damage–related transcripts in JJhan-PDLIM2i cells (Fig 5A–5C). Of note, HHV-6A infection enhanced NF-κB-regulated transcripts in control JJhan-CTi cells, as expected (S3A–S3D Fig). RT-qPCR validation supported this pattern, with higher induction of CXCL8 and IFNB1 in infected JJhan-PDLIM2i cells than in infected control cells (S4E Fig). Consistent with these transcriptional changes, U14 accumulated in JJhan-PDLIM2i cells to 2.26-fold, whereas the late protein gQ1 decreased to 0.60-fold and γH2AX levels increased, consistent with enhanced infection-associated stress and reduced late viral protein accumulation (Fig 6A–6D). Functionally, CRISPRi-mediated PDLIM2 knockdown had little effect on proliferation in uninfected cells but reduced cell viability and increased Annexin V positivity during infection, while lowering HHV-6A genome copies in culture supernatants (Fig 6E). Thus, these findings are consistent with a model in which PDLIM2 contributes to a replication-permissive state by facilitating phospho-p65 (Ser536) downregulation and U14 turnover, thereby limiting prolonged antiviral signaling and nuclear stress.

### Nuclear U14 has the capacity to induce ATM-linked DNA damage signaling that is limited by PDLIM2

**Fig 7:**
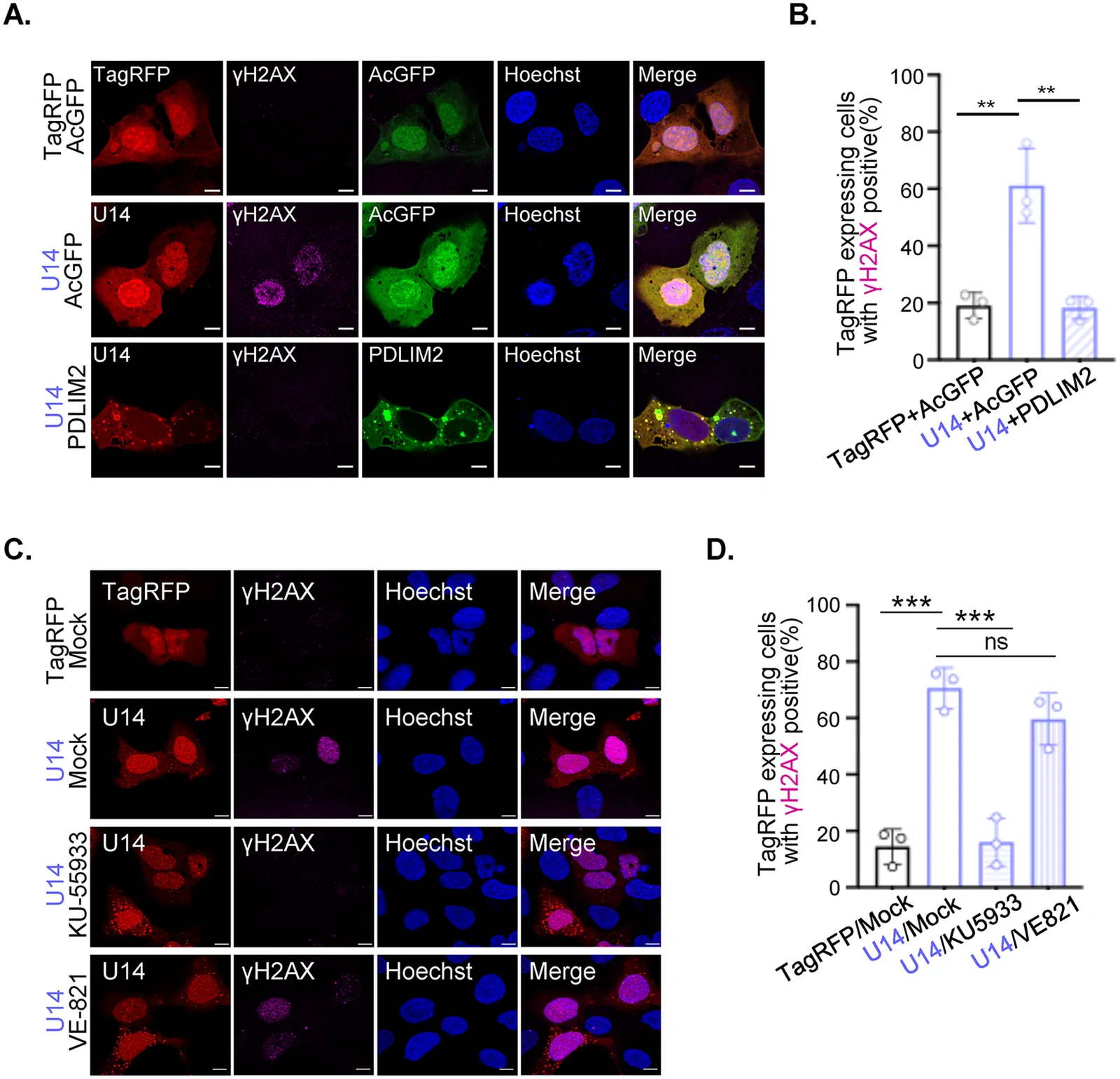
Nuclear U14 induces ATM-linked γH2AX accumulation that is constrained by PDLIM2. (A) Representative immunofluorescence images of U2OS cells expressing TagRFP, TagRFP-tagged HHV-6A U14, or TagRFP-tagged HHV-6A U14 together with PDLIM2–AcGFP, stained for γH2AX (magenta). Scale bars, 10 μm. (B) Quantification of the percentage of TagRFP-positive cells that were also γH2AX positive under the indicated conditions. (C) Representative immunofluorescence images of U2OS cells expressing TagRFP or TagRFP-tagged HHV-6A U14 and treated with KU-55933, VE-821, or vehicle control, followed by staining for γH2AX (magenta). Scale bars, 10 μm. (D) Quantification of the percentage of TagRFP-positive cells that were also γH2AX positive under the indicated conditions in (C). Data in (B and D) are shown as means ± SD from three independent experiments with n > 100 cells for each condition. One-way ANOVA followed by Tukey’s multiple-comparison test: **, p < 0.01; ***, p < 0.001; ns, not significant.

Because PDLIM2 knockdown was associated with increased nuclear U14 accumulation and elevated DNA damage response (DDR)-related transcripts (Fig 3D, 3E; Fig 5C), we examined whether nuclear U14 itself contributes to nuclear stress. Expression of TagRFP–HHV-6A U14 in U2OS cells increased the proportion of γH2AX-positive cells by 3.20-fold relative to the TagRFP control (Fig 7A, 7B). Consistently, doxycycline induction of U14 expression for 12 h in U2OS-Strep–FLAG–HHV-6A U14 cells increased γH2AX levels by 2.1-fold (S4A, S4B Fig). Co-expression of PDLIM2–AcGFP reduced the U14-induced γH2AX signal to 0.30-fold relative to the U14-alone condition, corresponding to an approximately 70% reduction (Fig 7A, 7B). Conversely, during HHV-6A infection, JJhan-PDLIM2i cells showed a 6.75-fold increase in γH2AX levels relative to control cells (S4C, S4D Fig). Together, these data support the intrinsic capacity of nuclear U14 to elicit γH2AX-marked nuclear stress and are consistent with PDLIM2 limiting this response during infection.

γH2AX can arise downstream of either ATM- or ATR-dependent DDR pathways. ATM responds predominantly to DNA double-strand breaks (DSBs), whereas ATR is activated by single-stranded DNA (ssDNA)-containing replication intermediates. To define the DDR pathway engaged by nuclear U14, we mapped γH2AX induction using kinase inhibitors. Treatment with the ATM inhibitor KU-55933 reduced HHV-6A U14-induced γH2AX, whereas the ATR inhibitor VE-821 had a smaller effect (Fig 7C, 7D), supporting an ATM-dominant DDR downstream of U14. Consistent with an ATM-linked, DSB-like damage signature, HHV-6A U14 was associated mainly with 53BP1 focus formation but limited representation of RPA2-positive foci (S5 Fig).

**Fig 8:**
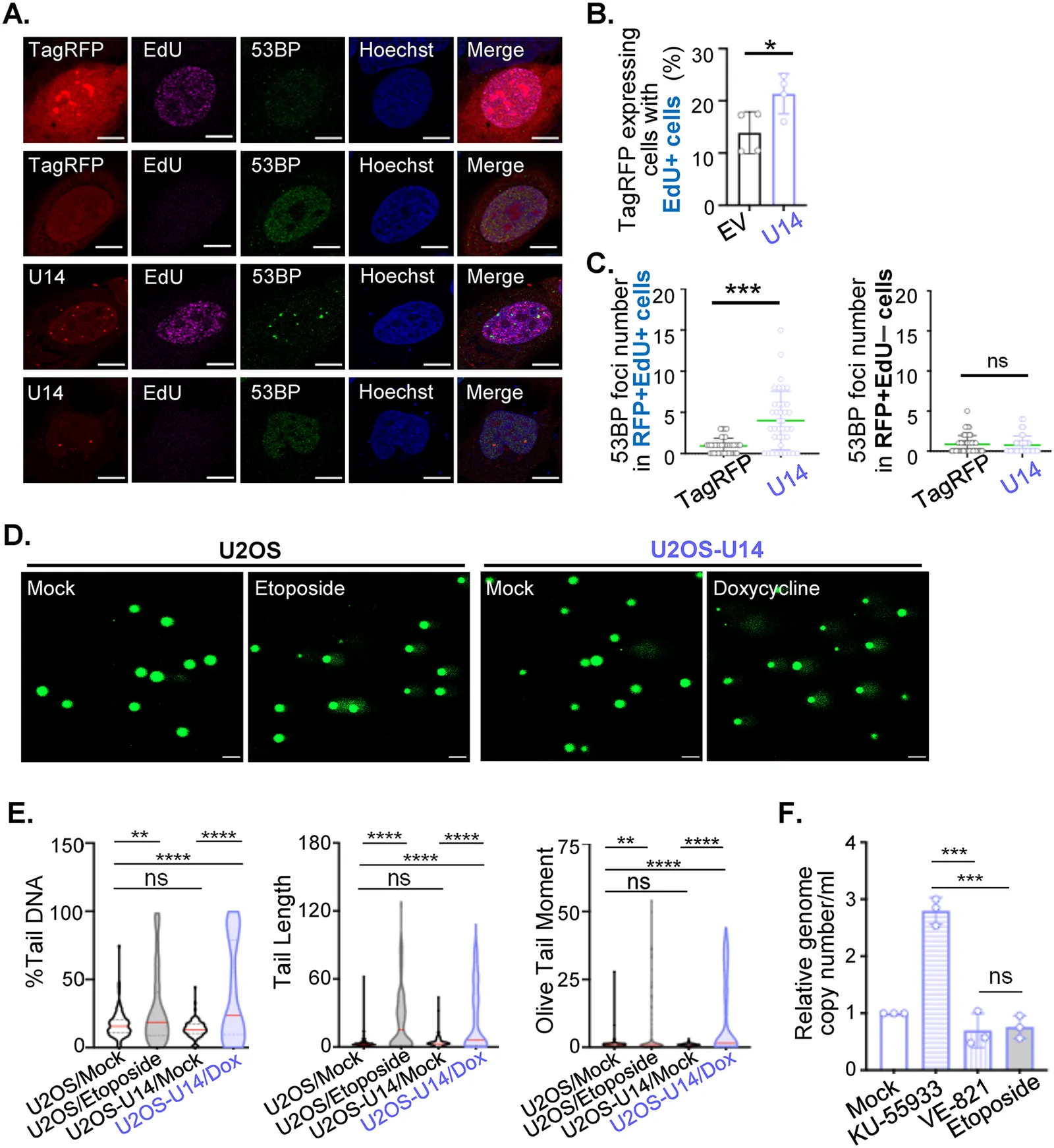
U14 induces DNA synthesis-associated DSB-like damage signaling, while ATM inhibition increases extracellular HHV-6A genome copies. (A) Representative immunofluorescence images of U2OS cells expressing TagRFP or TagRFP-tagged HHV-6A U14 for 24 h, pulse-labeled with EdU for 30 min before fixation, and stained for EdU-Alexa Fluor 647 (magenta), 53BP1 (green), and Hoechst 33342. Scale bars, 10 μm. (B) Quantification of the percentage of TagRFP-positive cells that were also EdU positive under the indicated conditions. Data are shown as means ± SD from three independent experiments with n = 300–500 cells for each condition. Two-tailed Student’s t-test: *, p < 0.05. (C) Quantification of the number of 53BP1 foci in TagRFP-positive EdU-positive cells (left) and TagRFP-positive EdU-negative cells (right). Each point represents a single cell. Horizontal lines indicate the median ± SD with n > 38 cells for each condition. Two-tailed Student’s t-test: ***, p < 0.001; ns, not significant. (D) Neutral comet assays in U2OS cells treated with etoposide as a positive control or vehicle, and in doxycycline-inducible U2OS-Strep–FLAG–HHV-6A U14 cells with or without induction. Images show representative comets from three independent experiments. Scale bars, 100 μm. (E) DNA breaks were quantified from neutral comet assays as % tail DNA, tail length, and Olive tail moment. Violin plots show single-cell distributions with n > 100 cells per condition, from one representative experiment; center lines denote the median, and diagonal hatch marks indicate the first and third quartiles. Statistical significance across experiments was determined by a linear mixed-effects model. Significance: **, p < 0.01; ****, p < 0.0001; ns, not significant. (F) JJhan cells were infected with HHV-6A for 48 h and then treated for an additional 24 h with KU-55933 (10 nM), VE-821 (1 μM), etoposide (10 μM), or vehicle. Viral genome copy numbers in culture supernatants were quantified by qPCR and normalized to the vehicle-treated infected control. Data are shown as means ± SD from three independent experiments. Statistical significance in (F) was determined by one-way ANOVA with Tukey’s multiple-comparison test: ***, p < 0.001; ns, not significant.

Because replication-associated lesions can progress to DSB-like breaks when fork perturbation persists, and γH2AX often marks DNA break formation arising from replication stress, we next asked whether the U14-associated DNA damage signals were enriched during ongoing DNA synthesis. To address this, we stratified U14-expressing cells by S-phase status using EdU pulse labeling. U14 modestly increased the EdU-positive fraction among TagRFP-positive cells and selectively increased 53BP1 focus formation within the EdU-positive subset, with minimal change in EdU-negative cells (Fig 8A–8C). This compartmental pattern indicates that U14-associated DNA damage signals are preferentially enriched in cells undergoing DNA synthesis.

To determine whether these DDR signals coincide with the accumulation of DSB-like DNA breaks, we performed neutral comet assays in the doxycycline-inducible U14 expression system. U14 expression increased the percentage of tail DNA, tail length, and Olive tail moment, approaching the values observed in etoposide-treated cells (Fig 8D, 8E), consistent with the accumulation of DSB-like DNA breaks in U14-expressing cells. Together, the preferential suppression of U14-induced γH2AX by the ATM inhibitor KU-55933 (Fig 7C, 7D), the S-phase enrichment of 53BP1 foci (Fig 8A–8C), and the neutral comet readout (Fig 8D–8E) support a model in which nuclear U14 has the capacity to promote ATM-linked, DSB-like DNA damage signaling associated with DNA synthesis-coupled stress [23–25]. Consistent with this framework, prior work showing that U14 associates with EDD, a mediator of DNA damage signal transduction, and induces G2/M arrest supports the idea that U14 engages a DDR-linked checkpoint program [26]. Functionally, ATM inhibition during the late phase of infection increased HHV-6A genome copies in culture supernatants to 2.79-fold relative to vehicle-treated controls without measurably impairing cell viability (Fig 8F; S6 Fig), suggesting that ATM signaling limits extracellular HHV-6A genome accumulation in this context and that timely attenuation of U14-associated DDR signaling may reduce cumulative nuclear stress over multi-day infection cycles.

**Fig 9:**
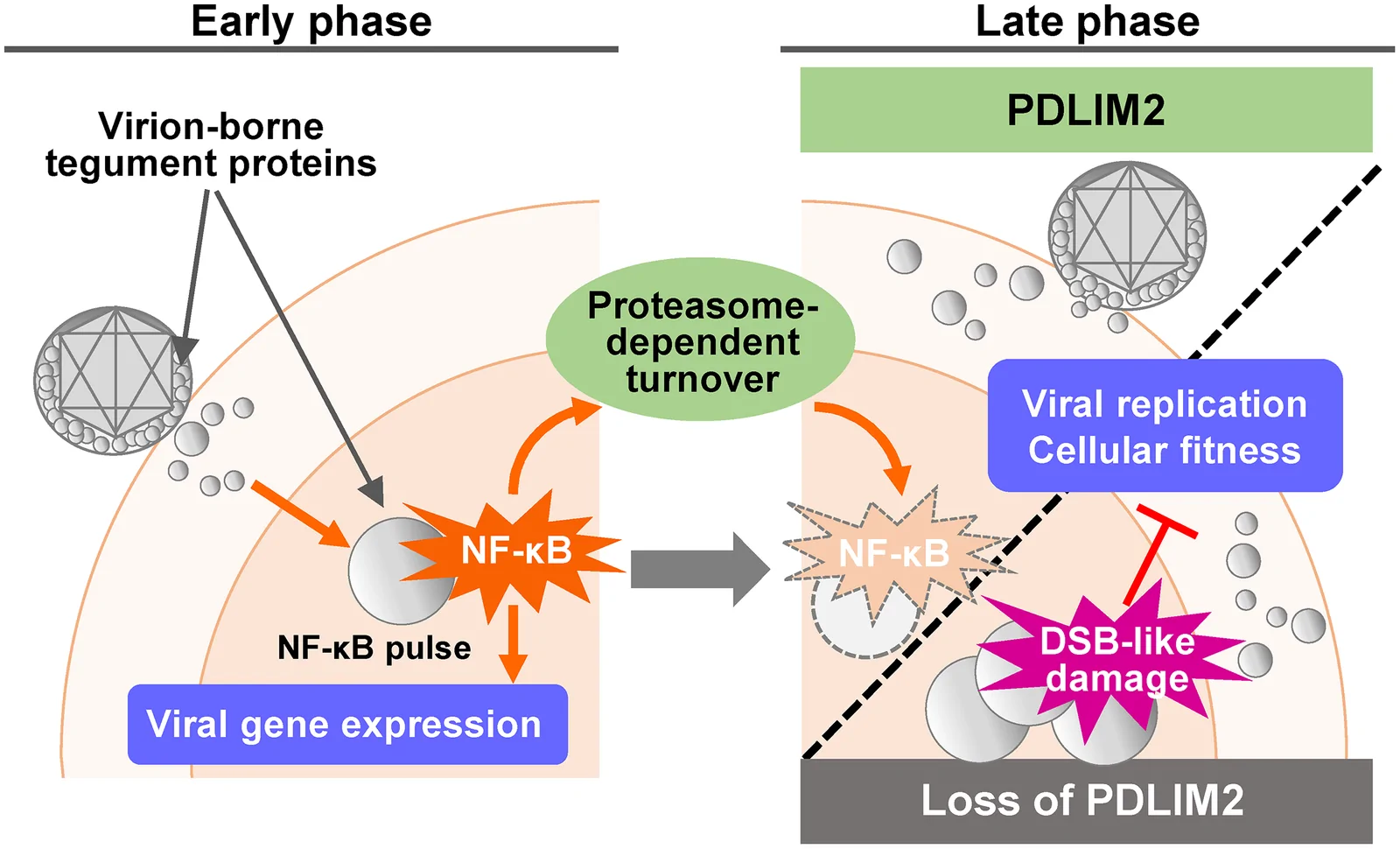
Schematic model of U14-linked NF-κB signaling and PDLIM2-mediated termination during HHV-6A infection. In the early phase, multiple entry- and infection-associated inputs, including virion-delivered U14, may contribute to a transient NF-κB response that supports viral gene expression. In the late phase, the ubiquitin adaptor PDLIM2 promotes proteasome-dependent phospho-p65 downregulation and U14 turnover. This timely termination limits prolonged NF-κB and antiviral signaling, persistent nuclear U14 accumulation, and ATM-linked DSB-like DNA damage signaling, and may thereby support virion production and cellular fitness. Under PDLIM2-knockdown conditions, nuclear U14 and NF-κB signaling persist, leading to increased DNA damage signaling and reduced extracellular HHV-6A genome copies.

Collectively, our data support a timing-based model for HHV-6A infection, in which infection-associated NF-κB signaling, to which U14 contributes, is actively limited together with nuclear U14 accumulation through a proteasome-dependent mechanism. This process limits excessive antiviral signaling and ATM-linked DNA damage signaling while supporting viral gene expression and extracellular viral genome accumulation over prolonged infection cycles (Fig 9). This timing model supports a role for PDLIM2 as a host factor that promotes activated-p65 (RelA) downregulation and U14 turnover, thereby preventing prolonged activation of U14-linked antiviral and DNA damage-associated responses. Together, these findings define the U14–PDLIM2 axis as a regulatory mechanism that coordinates NF-κB termination with control of nuclear stress during HHV-6A infection.

## Discussion

During HHV-6A infection, U14 has the intrinsic capacity to induce NF-κB signaling and contributes to an infection-associated NF-κB pulse whose waveform likely reflects multiple inputs, including viral entry, U14, subsequent viral gene expression, and host and viral regulatory mechanisms. Rather than suppressing infection, PDLIM2 limits the persistence of this response by facilitating phospho-p65 (Ser536) downregulation and U14 turnover, thereby preserving cellular fitness and supporting viral gene expression and extracellular HHV-6A genome accumulation. Failure of this regulation may have multiple adverse consequences, including prolonged NF-κB and interferon-associated responses and nuclear stress associated with persistent U14. Our data identify ATM-linked DNA damage signaling as one functionally relevant late-phase consequence, although it is unlikely to fully explain the broader phenotype of CRISPRi-mediated PDLIM2 knockdown.

Mechanistically, PDLIM2 functions as the timer–terminator for an HHV-6A infection-associated NF-κB response to which U14 contributes. U14 associates with p65 and has the intrinsic capacity to promote NF-κB activation, whereas PDLIM2 associates with U14, is associated with enhanced ubiquitin modification of U14, and promotes proteasome-dependent U14 turnover together with the downregulation of phospho-p65 (Ser536). CRISPRi-mediated PDLIM2 knockdown was associated with prolonged phospho-p65 signaling and increased nuclear U14 accumulation, elevated innate immune and DDR-related programs, compromised cellular fitness during infection, and reduced extracellular HHV-6A genome copies. These findings support a double-edged model in which nuclear U14 contributes to NF-κB activation and viral gene expression but becomes detrimental when it persists into late phases. By coupling phospho-p65 downregulation to U14 turnover, PDLIM2 limits the late-phase costs of sustained antiviral signaling and persistent nuclear U14, including ATM-linked DNA damage signaling.

Prior work shows that entry-delivered tegument proteins act early in the nucleus and subsequently diminish in abundance or relocalize as infection progresses. For example, the HCMV tegument protein pp71 (UL82) enters the nucleus to antagonize DAXX/ATRX and de-repress immediate-early transcription, after which its nuclear abundance decreases or redistributes as infection advances [27–29]. Similarly, the HSV-1 tegument protein VP16 (UL48) is imported into the nucleus soon after entry to assemble the VP16-induced complex and activate immediate-early genes, and later changes its subcellular localization [30–32]. Taken together, these observations support a broader view that nuclear residence of tegument proteins is temporally regulated during herpesvirus infection. Our findings extend this concept by showing that, in HHV-6A infection, turnover of a nuclear tegument protein is functionally linked to termination of an NF-κB pulse and control of nuclear stress. Importantly, this view does not require that all nuclear tegument proteins initiate DDR or use the same host adaptor pathway.

Several considerations refine the scope of these conclusions. Additional ubiquitin adaptors and E3 ligases may operate in a cell-type- or time-dependent manner. Although PDLIM2 is associated with enhanced ubiquitin modification of U14 and promotes its proteasome-dependent turnover, it is not itself an E3 ubiquitin ligase, and the E3 ligase that directly catalyzes U14 ubiquitination remains to be identified. Both U14 shRNAs targeted distinct sequences within the U14 coding region, neither of which directly overlaps the reported mature miR-aU14 sequence or any annotated adjacent protein-coding ORFs. However, because miR-aU14 is transcribed antisense from within the U14 locus and its expression was not measured in the present study, indirect effects of the shRNAs on miR-aU14 expression or processing cannot be excluded. In addition, because the full-length transcript architecture surrounding HHV-6A U14 has not been completely defined, the shRNAs may also deplete longer U14-containing sense transcripts, if such transcripts are expressed. Nevertheless, the concordant phenotypes produced by two independent shRNAs, together with the observed reduction in U14 protein abundance, support a contribution of U14 protein depletion to the observed effects.

Some mechanistic assays relied on the ectopic expression of U14 and PDLIM2. This experimental strategy should be interpreted as a reductionist approach that reveals the intrinsic capacity of U14 to induce NF-κB signaling and ATM-linked, DSB-like DNA damage signaling, rather than as a quantitative representation of the infected-cell state. This distinction is especially important for tegument proteins, because the level of virion-delivered tegument protein present immediately after entry is expected to be much lower than that achieved by ectopic expression or by accumulation during late infection. Consistent with this point, U14 was undetectable or only faintly detectable by immunoblotting at early times after infection, whereas phospho-p65 (Ser536) was already detectable; therefore, the magnitude and kinetics of signaling observed after ectopic U14 expression should not be directly extrapolated to infection. During HHV-6A infection, U14 is delivered and expressed together with other viral factors, and herpesviruses encode multiple proteins that modulate DDR. Consistent with this interpretation, CRISPRi-mediated PDLIM2 knockdown during HHV-6A infection was associated with increased nuclear U14 accumulation and enhanced γH2AX levels, whereas both nuclear U14 and γH2AX signals were limited in control-infected cells. Thus, the relatively limited γH2AX signal observed in infected cells may reflect the combined effects of viral DDR modulators and host regulatory mechanisms, including PDLIM2-dependent attenuation of nuclear U14 persistence. Testing these effects at endogenous expression levels and conducting broader virological phenotyping, including in primary infection contexts, will further strengthen the generalizability of these findings beyond the systems studied here.

The relationship between DDR and DNA viruses has long been studied: early DDR activation can facilitate immediate-early gene expression, yet unresolved DDR becomes harmful [33–35]. This phase-dependent duality may underlie the variable effects of ATM inhibition reported across viral systems. In our experiments, ATM inhibition during the late phase increased extracellular HHV-6A genome copies, supporting a restrictive role for ATM-linked DDR at this stage of infection. Together with the increased nuclear U14 accumulation and γH2AX signaling following CRISPRi-mediated PDLIM2 knockdown, these findings identify U14-associated DDR as a functionally relevant late-phase cost of failed termination, although it is unlikely to fully account for the broader knockdown phenotype.

This work delineates a proteasome-dependent timing mechanism in which PDLIM2 couples phospho-p65 (Ser536) downregulation to U14 turnover. By limiting prolonged NF-κB signaling and U14-associated nuclear stress, including ATM-linked DDR, this mechanism preserves cellular fitness and promotes a replication-permissive state during HHV-6A infection.

## Materials and methods

### Cell lines

HEK293T and U2OS cells were cultured in Dulbecco’s modified Eagle medium (DMEM) supplemented with 8% fetal bovine serum (FBS) [36–38]. The JJhan human T-lymphoblastoid cell line was cultured in RPMI-1640 medium containing L-glutamine (Sigma-Aldrich) with 8% FBS [8,39]. Umbilical cord blood mononuclear cells (CBMCs) were cultured as described [8,39,40]. CBMCs were purchased from the Cell Bank of the RIKEN BioResource Center, Tsukuba, Japan. The use of CBMCs in this study was approved by the Ethics Committee of Kobe University Graduate School of Medicine (approval number: No.1209). The samples had been anonymized before acquisition from the RIKEN BioResource Center, and the authors had no access to personally identifiable information. Therefore, informed consent was not required for this study. Transfection experiments were performed using Lipofectamine 3000 (Thermo Fisher Scientific).

### Viruses

Human herpesvirus 6A (HHV-6A) strain U1102 was propagated in cord blood mononuclear cells (CBMCs) as previously reported [39,41–43]. For infecting the target cell line JJhan with HHV-6A virus stock, cells were collected by centrifugation, resuspended in RPMI medium with virus stock containing 1 × 10^7^ genome copies of the virus or medium only as a mock control. The cells were then centrifuged at 35 °C for 30 min at 240 g, followed by culturing in RPMI supplemented with 2% FBS. Infected JJhan cells were centrifuged at 880 × g for 5 min at room temperature, and the supernatants were collected and subjected to DNA extraction, whereas the cell pellets were used for RNA extraction to analyze host and viral gene expression and for immunoblot analysis.

### Plasmids

The entire coding sequence of HHV-6A U14, fused to either an HA tag or a Strep–FLAG tag, was cloned into the pCAGGS-MCS expression vector and used for transient expression analyses [8]. The TagRFP sequence was amplified by PCR from pTagRFP-C (Evrogen) and cloned into pCAG-Strep–FLAG–HHV-6A U14 to generate pTagRFP–HHV-6A U14, expressing a TagRFP–HHV-6A U14 fusion protein. Plasmid pPIDCBneo is an empty Dox-inducible vector with piggyBac [42,44]. The coding sequence of mScarlet-1 in pPIDCBneo was replaced with that of Strep–FLAG–HHV-6A U14 from pCAG-Strep–FLAG–HHV-6A U14 or FLAG–HHV-6A U29 from HHV-6A U1102 genome to design pPIDCBneo-Strep–FLAG–HHV-6A U14 or pPIDCBneo-FLAG–HHV-6A U29, respectively. The entire coding sequence of PDLIM2 was cloned into pCMV14 (Sigma) or pAcGFP-N1 (Takara) to express a PDLIM2–FLAG or PDLIM2–AcGFP fusion protein, respectively. The HA-Ub expression plasmid, designed to express ubiquitin fused with an HA tag, was constructed by cloning the ubiquitin coding sequence linked to the HA tag into the pcDNA3.1-mycHis (–) vector (Thermo Fisher Scientific). To generate the pLV-KRAB-PDLIM2 lentiviral vector expressing a gRNA targeting human PDLIM2, coupled to dCas9-KRAB, complementary oligonucleotides (5′-GCCTGGGCCTGGAGGAACGA-3′) were annealed and cloned into pLV hU6-sgRNA hUbC-dCas9-KRAB-T2a-Puro (Addgene #71236) [45]. To generate lentiviral shRNA plasmids targeting HHV-6A U14 (shU14-1 and shU14-2), sense and antisense oligonucleotides were annealed and cloned into pLKO**.**1-puro (Addgene #8453) [46]. The oligonucleotide sequences were: shU14A-1, 5′-CCGGTGGTTATCATCACAAAGTTTACTCGAGTAAACTTTGTGATGATAACCATTTTTG-3′; and 5′-AATTCAAAAATGGTTATCATCACAAAGTTTACTCGAGTAAACTTTGTGATGATAACCA-3′; and shU14A-2, 5′-CCGGAACCTTAAACTCCTTTCATAACTCGAGTTATGAAAGGAGTTTAAGGTTTTTTTG-3′; and 5′-AATTCAAAAAAACCTTAAACTCCTTTCATAACTCGAGTTATGAAAGGAGTTTAAGGTT-3′. These oligonucleotides were used to generate pLKO**.**1-puro-U14A-1 or pLKO**.**1-puro-U14A-2, respectively. The corresponding 21-nt HHV-6A U14 target sequences were 5′-TGGTTATCATCACAAAGTTTA-3′ for shU14A-1 and 5′-AACCTTAAACTCCTTTCATAA-3′ for shU14A-2. The two non-overlapping target sites are located within the HHV-6A U14 coding region and do not extend into any adjacent annotated protein-coding open reading frames. pLKO**.**1-puro non-targeting shRNA plasmid (shNT) (Addgene #109012) [46] was used as a control.

### Construction of cell lines

U2OS cells were transfected with pPIDCBneo-Strep–FLAG–HHV-6A U14, pPIDCBneo, or pPIDCBneo-FLAG–HHV-6A U29 together with a hyPBase expression cassette amplified by PCR from pPB[Exp]-Puro-CAG > hyPBase (Vector Builder) using Lipofectamine 3000 (Invitrogen) following the manufacturer’s protocol. Twenty-four hours post-transfection, cells were placed under G418 selection for 10–14 days with medium changes every 2–3 days. Resistant polyclonal cells were expanded. Inducible expression was verified by doxycycline treatment (1 µg/mL, 24 h) followed by immunoblotting with anti-FLAG. The resulting lines were referred to as U2OS-Strep–FLAG–HHV-6A U14, U2OS-mScarlet-1, or U2OS-FLAG–HHV-6A U29, respectively.

To generate CRISPRi-mediated knockdown of PDLIM2 in JJhan cells, HEK293T cells were transfected with either pLV hU6-sgRNA hUbC-dCas9-KRAB-T2a-Puro or pLV-KRAB-PDLIM2 together with the packaging plasmids pCAG-HIV-gp and pCMV-VSV-G-RSV-Rev, as previously described [39]. At 48 h post-transfection, virus-containing supernatants were collected and used to transduce JJhan cells, which were then selected with puromycin (1 μg/mL). The puromycin-resistant populations were designated JJhan-CTi and JJhan-PDLIM2i.

To generate shRNA-mediated knockdown of HHV-6A U14 in JJhan cells, HEK293T cells were transfected with either pLKO.1-puro or pLKO.1-puro-shU14A-1 or pLKO.1-puro-shU14A-2 together with the packaging plasmids pCAG-HIV-gp and pCMV-VSV-G-RSV-Rev. At 72 h post-transfection, virus-containing supernatants were collected and used to transduce JJhan cells, which were then selected with puromycin (1 μg/mL). The puromycin-resistant populations were designated JJhan-shCT, JJhan-shU14A-1, or JJhan-shU14A-2.

### Antibodies and reagents

For immunoblotting and immunofluorescence analysis, we used mouse monoclonal antibodies against β-actin (AC15; Sigma), FLAG (M2; Sigma), GFP antibody (JL-8; Takara), IκBα (L35A5; Cell Signaling Technology), HA (M180-3; MBL), Phospho-Histone H2A.X (Ser139) (D7T2V; Cell Signaling Technology) for immunoblot and IFA, and Strep-tag II (4F1; MBL); rabbit monoclonal antibodies against p65 (D14E12; Cell Signaling Technology), Phospho-NF-κB p65 (Ser536) (93H1; Cell Signaling Technology), H2A.X (ab124781, Abcam), Phospho-Histone H2A.X (Ser139) (20E3; Cell Signaling Technology) for IFA and immunoblotting, Phospho-Chk1 (Ser345) (133D3; Cell Signaling Technology), Phospho-Chk2 (Thr68) (C13C1; Cell Signaling Technology) and 53BP1 (ab175933; Abcam); rabbit polyclonal antibody against RFP (pabr1, Proteintech); and a rat monoclonal antibody against RPA2 (4E4; Cell Signaling Technology). Rabbit polyclonal antibody against U14 and mouse monoclonal antibodies against HHV-6A U14 (BU14) and gQ1 (AgQ1-119) were produced and used as previously described [8,39]. SC75741, QNZ (EVP4593), KU55933, VE-821, and MG132 were purchased from Selleck Chemicals. Etoposide was purchased from FUJIFILM Wako.

### Immunoblotting and immunofluorescence

Immunoblotting and immunofluorescence were performed as described previously [37,47–49]. Immunoblot images were acquired with a Fusion FX imaging system (Vilber), and band intensities were quantified using EvolutionCapt Edge software (Vilber). For immunofluorescence, the cells were fixed with 4% paraformaldehyde (for transfected cells) or methanol/acetone (for infected cells) and stained with the indicated antibodies. Nuclear DNA was stained with Hoechst 33342 (Dojindo), and specific signals were detected using a confocal laser-scanning microscope (LSM800 microscope; Zeiss). For quantification of fluorescence, images of each cell acquired on the LSM 800 were analyzed using the Histo function in ZEN 3.1 software (Zeiss) as previously reported [50,51].

### Affinity precipitation

HEK293T cells were transfected with a plasmid expressing Strep–Flag–HHV-6A U14 or an empty plasmid (EV), together with a plasmid expressing PDLIM2-AcGFP. After 48 h, cells were harvested and lysed with 0.1% NP-40 buffer (50 mM Tris-HCl [pH 8.0], 150 mM NaCl, 0.1% NP-40) containing a protease inhibitor cocktail (Nacalai Tesque). Lysates were clarified by centrifugation, and the supernatants were incubated with Strep-Tactin magnetic beads (IBA) with rotation for 2h at 4°C. Beads were collected using a magnetic rack, washed extensively with 0.1% NP-40 buffer, and bound proteins were analyzed by immunoblotting.

### Immunoprecipitation

HEK293T cells were transfected with a plasmid expressing PDLIM2-AcGFP or an empty plasmid (EV), together with a plasmid expressing HA-HHV-6A U14. After 48 h, cells were harvested and lysed with 0.5% NP-40 buffer (50 mM Tris-HCl [pH 7.5], 150 mM NaCl, 0.5% NP-40) containing a protease inhibitor cocktail (Nacalai Tesque). Lysates were clarified by centrifugation, and the supernatants were incubated with GFP antibody and Protein A/G magnetic beads (Thermo Fisher Scientific) with rotation for 4 h at 4°C. Beads were collected using a magnetic rack, washed extensively with 0.1% NP-40 buffer, and bound proteins were analyzed by immunoblotting.

### Ubiquitination pulldown assay

HEK293T cells were co-transfected with pCAG-Strep–FLAG–HHV-6A U14, AcGFP–PDLIM2, and pcDNA-HA-Ub. After 24 h, cells were treated with MG132 (20 μM) for 6 h. Cells were lysed in denaturing buffer (50 mM Tris-HCl, pH 8.0; 150 mM NaCl; 1% NP-40; 0.5% sodium deoxycholate; 1% SDS; 1 mM PMSF; 10 mM iodoacetamide [FUJIFILM Wako]), sonicated, and boiled (95 °C, 10 min). Lysates were clarified (15,000 × g, 10 min, 4 °C), diluted to a final SDS concentration of <0.1%, and incubated overnight with Strep-Tactin magnetic beads (IBA). Beads were washed five times with 0.1% NP-40 buffer and eluted in 2 × SDS sample buffer. Inputs and eluates were analyzed by immunoblotting with anti-HA, anti-FLAG, and anti-GFP antibodies.

### Quantitative PCR (qPCR)

RNA was extracted with NucleoSpin RNA (Macherey-Nagel) according to the manufacturer’s instructions, and cDNA was synthesized from the isolated RNA with SuperScript IV (Thermo Fisher Scientific). qPCR was performed with Power SYBR Green Master Mix (Thermo Fisher Scientific) on a QuantStudio 1 instrument (Thermo Fisher Scientific). Viral DNA was extracted using the DNeasy Blood and Tissue Kit (QIAGEN) and quantified by U67 primers (5′-CGCTAGGTTGAGAATGATCGA-3′ and 5′-CAAAGCCAAATTATCCAGAGCG-3′) as described previously [8,39]. The primer sequences for IFN-β were 5′-CTTGGATTCCTACAAAGAAGCAGC-3′ and 5′-TCCTCCTTCTGGAACTGCTGCA-3′; The primer sequences for TNFA were 5′-CTCTTCTGCCTGCTGCACTTTG-3′(forward) and 5′-ATGGGCTACAGGCTTGTCACTC-3′ (reverse); those for IL-6 were 5′-GACCCAACCACAAATGCCA-3′ (forward) and 5′-GTCATGTCCTGCAGCCACTG-3′ (reverse); those for IL-8 were 5′-CTGGCCGTGGCTCTCTTG-3′ (forward) and 5′-CCTTGGCAAAACTGCACCTT-3′ (reverse). The primer sequences for U14 were 5′-TTCGACACCGAAGAAGCCA-3′ (forward) and 5′-GTCCGGTCGATTATGAAAGGAG-3′(reverse); those for U27 were 5′-AAAGCACGTTGTTGACGGTG-3′ (forward) and 5′-CCTGTTTTGATGCCAACGCA-3′ (reverse); those for U38 were 5′-GATGTGTGGTGTTCGAGGGT-3′ (forward) and 5′- TGCGAAGACACCATACTCGG-3′ (reverse); those for PDLIM2 were 5′-GTATGGCGTTGACGGTGGATG-3′ and 5′-GGAGGTCAGCGTCCTTGGCT-3′; those for β-actin were 5′-GCACCCAGCACAATGAAGA-3′ and 5′-CGATCCACACGGAGTACTTG-3′;

### Neutral comet assay

Neutral comet assays were performed essentially as described previously [52,53], with minor modifications. U2OS cells treated with etoposide (20 μM, 1 h) or mock-treated, and U2OS-Strep–FLAG–HHV-6A U14 cells induced with doxycycline (1 μg/mL, 24 h) or mock-treated, were collected. Cells were embedded in 0.5% low-melting-point agarose (Nippon Gene) on slides pre-coated with 1% normal agarose (Nacalai Tesque). Slides were immersed in neutral lysis buffer (2.5 M NaCl; 100 mM EDTA; 10 mM Tris-HCl, pH 10.0; 1% Triton X-100; 10% DMSO) for 2 h at 4 °C. Electrophoresis was performed in 1 × TBE buffer (90 mM Tris; 90 mM Boric acid; 2 mM EDTA; pH 8.0) at 18 V for 30 min. DNA was stained with GelGreen Nucleic Acid Stain (Biotium). Images were acquired on a Zeiss LSM 800 confocal microscope using an FITC filter set and processed with ZEN 3.1 software. Comet metrics were quantified using CometScore 2.0 (TriTek) software.

### Immunofluorescence-based DNA damage response (DDR) assays

U2OS cells were transfected with pTagRFP–HHV-6A U14 for 24 h. Where indicated, cells were treated with the ATM inhibitor KU-55933 (10 nM), the ATR inhibitor VE-821 (1 μM) or etoposide (10 μM) for 1 h. Cells were then fixed and analyzed by immunofluorescence staining.

### Cell viability assay

Cell viability was assessed using two complementary approaches. For metabolic activity, cells were cultured in flat-bottom 96-well plates at 2 × 10^3^ cells per well in 100 µL complete medium. Where indicated, cells were treated with compounds for the specified times before the addition of Cell Counting Kit-8 (CCK-8; Dojindo) reagent. After incubation at 37 °C, absorbance at 450 nm was measured using a microplate reader, and values were normalized to mock-treated controls. To monitor apoptosis and loss of membrane integrity, cells were harvested together with culture supernatants and stained with Annexin V-633 and propidium iodide (PI) (Nacalai Tesque) in Annexin V-633 binding buffer. Samples were analyzed by flow cytometry on a Sony SA3800 instrument. Early apoptotic (Annexin V ⁺ /PI^-^) and late apoptotic (Annexin V ⁺ /PI⁺) populations were quantified. All experiments were performed in biological triplicate. Data and images were analyzed with Floreada.io and SA3800 software.

### EdU incorporation

U2OS cells were transfected with TagRFP empty vector or TagRFP-U14. At 24 h post-transfection, cells were pulse-labeled with 10 µM EdU for 30 min at 37°C, washed once with PBS, and fixed with 4% paraformaldehyde in PBS for 15 min at room temperature. Cells were permeabilized with 0.5% Triton X-100 in PBS for 20 min and EdU was detected using the Click-iT EdU Imaging Kit (Alexa Fluor 647) (Invitrogen) reaction cocktail prepared immediately before use and incubated for 30 min at room temperature protected from light. After washing with 3% BSA in PBS, cells were processed for immunofluorescence assay.

### RNA-seq library preparation and sequencing

JJhan-CTi or JJhan-PDLIM2i cells (1 × 10^5^) were mock-infected or infected with HHV-6A U1102 for 72 h. Under these conditions, about 20% of JJhan cells were found to be infected as determined by immunofluorescence. RNA was extracted using the Maxwell RSC simplyRNA kit (Promega). Sequencing libraries were generated using NEBNext Poly(A) mRNA Magnetic Isolation Module and NEBNext Ultra II Directional RNA Library Prep Kits (NEB) following the manufacturer’s recommendations. After cluster generation, the library preparations were sequenced on an Illumina NovaSeq X Plus. Read count tables were processed in R (v4.0.3). Differential expression was modeled using DESeq2 (v1.30) with the formula *design = ~ group*. Log_2_ fold changes were moderated with apeglm shrinkage [54,55]. Genes were considered differentially expressed at Benjamini–Hochberg adjusted *p* < 0.05 and |log_2_FC| ≥ 1. For gene set enrichment analysis, Hallmark gene sets were obtained from Molecular Signatures Database (MSigDB) via the msigdbr R package (v25.1.1) [56,57]. Heatmaps were generated with Heatmapper.ca [58].

### Infection assay with inhibitors

JJhan cells (1 × 10^5^ per well) were infected with HHV-6A U1102 (1 × 10^7^ genome copies per well). At 48 hpi, cultures were treated for an additional 24 h with KU-55933 (10 nM), VE-821 (1 μM), etoposide (10 μM), or vehicle. Supernatants were then collected, and viral DNA was quantified.

### Statistical analysis

All experiments were independently repeated at least three times. Data are presented as mean ± SD. Statistical analyses were performed using GraphPad Prism (v8.4.3) and R (v4.0.3). Unless otherwise specified, significance was assessed with an unpaired two-tailed Student’s t-test. For comparisons involving more than two groups, one-way ANOVA followed by Tukey’s post hoc test was used. Statistical significance was defined as p < 0.05. Graph annotations: *; p < 0.05, **; p < 0.01, ***; p < 0.001, ****; p < 0.0001, ns; not significant.

### Software

R (v4.0.3), GraphPad Prism (v8.4.3), SA3800 software (Sony), Floreada.io, Heatmapper.ca, EvolutionCapt Edge (Vilber), iBright (Thermo Fisher Scientific), and ZEN 3.1 (Zeiss) were used.

### Data availability

RNA sequencing data have been registered in the DNA Data Bank of Japan under BioProject accession number PRJDB39636.

## Supporting information

S1 Fig Early kinetics and specificity of the U14-induced NF-κB pulse. (A) Immunoblots for phospho-p65 (Ser536), total p65, IκBα, FLAG–U14, and β-actin of doxycycline-inducible U2OS-Strep–FLAG–HHV-6A U14 cells collected at the indicated times after induction. Representative of three independent experiments. (B) Immunoblots for phospho-p65 (Ser536), total p65, mScarlet-1, and β-actin in doxycycline-inducible U2OS-mScarlet-1 cells collected at the indicated times after induction. Representative of three independent experiments. (C) Immunoblots for phospho-p65 (Ser536), total p65, FLAG–U29, and β-actin of doxycycline-inducible U2OS-FLAG–HHV-6A U29 cells collected at the indicated times after induction. Representative of three independent experiments. (D) Densitometric quantification of phospho-p65 (Ser536) normalized to total p65 in doxycycline-inducible U2OS-mScarlet-1, U2OS-Strep–FLAG–HHV-6A U14, and U2OS-FLAG–HHV-6A U29 cells. (E) Densitometric quantification of IκBα normalized to β-actin in doxycycline-inducible U2OS-Strep–FLAG–HHV-6A U14 cells at the indicated time points. Data are means ± SD (n = 3). Statistical significance was assessed by two-way ANOVA followed by Dunnett’s multiple-comparison test: *, p < 0.05; **, p < 0.01; ***, p < 0.001; ****, p < 0.0001; ns, not significant.
(PDF)

S2 Fig Pharmacologic inhibition of NF-κB increases nuclear U14 accumulation. (A) Cell viability measured by CCK-8 assay under the conditions used in Fig 1G and H. Absorbance was measured at 450 nm. Data are shown as means ± SD from three independent experiments. (B) Representative images of HEK293T cells co-expressing TagRFP–HHV-6A U14 and PDLIM2–AcGFP for 24 h; cells were treated with the NF-κB inhibitor SC75741 (200 nM), QNZ (10 nM), or vehicle for the final 2 h before fixation. Scale bars, 10 μm. (C) Quantification of nuclear TagRFP–HHV-6A U14 intensity. Nuclear contours were traced from the Hoechst channel. Single-cell measurements from one representative experiment (n > 30 cells per condition); red horizontal lines indicate the median; diagonal hatch marks indicate the first and third quartiles. Statistical significance was assessed by one-way ANOVA followed by Tukey’s multiple-comparison test: ***, p < 0.001; ****, p < 0.0001. Independent experiments were repeated three times with similar results.
(PDF)

S3 Fig HHV-6A alters NF-κB- and DDR-linked transcripts, and PDLIM2 knockdown reshapes infection-associated gene expression. JJhan-CTi and JJhan-PDLIM2i cells were mock-infected or infected with HHV-6A for 72 h, and RNA was extracted for bulk RNA-seq. (A) Heatmap of NF-κB-regulated genes in mock-infected and HHV-6A-infected JJhan-CTi cells. (B) Heatmap of DDR-related genes in mock-infected and HHV-6A-infected JJhan-CTi cells. (C, D) Top 10 enriched Hallmark gene sets among genes downregulated (C) or upregulated (D) in HHV-6A-infected JJhan-PDLIM2i cells relative to HHV-6A-infected JJhan-CTi cells. (E) RT-qPCR validation of IFN-β and CXCL8 mRNA expression, normalized to β-actin. Data are means ± SD (n = 3); one-way ANOVA followed by Tukey’s multiple-comparison test. Significance: **, p < 0.01; ns, not significant.
(PDF)

S4 Fig PDLIM2 limits U14-associated DNA damage signaling during HHV-6A infection. (A) Immunoblot analysis of doxycycline-inducible U2OS-Strep–FLAG–HHV-6A U14 cells harvested at 0 or 12 h after doxycycline addition and probed with the indicated antibodies. β-actin was used as a loading control. (B) Densitometric quantification of γH2AX normalized to total H2AX from (A). Data are shown as means ± SD from three independent experiments. Two-tailed unpaired Student’s t-test: *, p < 0.05. (C) Representative immunofluorescence images of JJhan-CTi and JJhan-PDLIM2i cells infected with HHV-6A for 72 h, stained for U14 (green), γH2AX (red), and Hoechst 33342. For each condition, the panels directly below show magnified views of the corresponding white dashed boxes; nuclear γH2AX puncta are indicated by arrowheads. Scale bars, 10 μm. (D) Quantification of HHV-6A–infected cells exhibiting nuclear γH2AX puncta from (C). Data are means ± SD (n = 3); two-tailed unpaired Student’s t-test. Significance: ***, p < 0.001.
(PDF)

S5 Fig U14 expression is associated with 53BP1 nuclear foci. (A) U2OS cells were transfected with TagRFP–HHV-6A U14 or TagRFP for 24 h; as a positive control, TagRFP-expressing cells were treated with etoposide (10 μM, 1 h). Cells were stained for 53BP1 (magenta), RPA2 (green), and Hoechst 33342. Scale bars, 10 μm. (B) Distribution of nuclear foci patterns in U2OS cells transfected as in (A). Stacked bars represent the proportions of nuclei showing 53BP1 foci (magenta), RPA2 foci (green), both 53BP1 and RPA2 foci (black), or neither (gray) under the indicated conditions. Data are means from three independent experiments (n = 3; > 100 cells per condition).
(PDF)

S6 Fig DDR marker panel and viability control for kinase-inhibitor treatment. (A) Immunoblot analysis of JJhan cells mock-infected or infected with HHV-6A for 72 h. Cells were treated with KU-55933 (10 nM), VE-821 (1 μM), etoposide (10 μM), or vehicle for the final 24 h before harvest. Blots were probed for U14, viral glycoprotein gQ1, phospho-Chk1 (p-Chk1), phospho-Chk2 (p-Chk2), γH2AX, total H2AX, and β-actin as a loading control. For p-Chk1 and p-Chk2, both high- and low-exposure images are shown. (B) Cell viability of JJhan cells treated as in (A). Data are means ± SD (n = 3); one-way ANOVA followed by Tukey’s multiple-comparison test. Significance: ns, not significant.
(PDF)

S1 Raw Gel Original uncropped and unadjusted images underlying the blot results presented in Figs 1A, 1D, 1G, 2B, 2D–2F, 3B, 4A, and 6A and S1A–S1C, S4A, and S6A Figs. (PDF)

## References

[1] Purvis JE, Karhohs KW, Mock C, Batchelor E, Loewer A, Lahav G. p53 dynamics control cell fate. Science. 2012;336(6087):1440–4. doi: 10.1126/science.1218351 22700930 PMC4162876

[2] Purvis JE, Lahav G. Encoding and decoding cellular information through signaling dynamics. Cell. 2013;152(5):945–56. doi: 10.1016/j.cell.2013.02.005 23452846 PMC3707615

[3] Dorrington MG, Fraser IDC. NF-κB signaling in macrophages: dynamics, crosstalk, and signal integration. Front Immunol. 2019;10:705. doi: 10.3389/fimmu.2019.00705 31024544 PMC6465568

[4] Sucharita S, Krishnagopal A, van Drunen Littel-van den Hurk S. Comprehensive analysis of the tegument proteins involved in capsid transport and virion morphogenesis of alpha, beta and gamma herpesviruses. Viruses. 2023;15(10):2058. doi: 10.3390/v15102058 37896835 PMC10611259

[5] Compton T, Kurt-Jones EA, Boehme KW, Belko J, Latz E, Golenbock DT, et al. Human cytomegalovirus activates inflammatory cytokine responses via CD14 and Toll-like receptor 2. J Virol. 2003;77(8):4588–96. doi: 10.1128/jvi.77.8.4588-4596.2003 12663765 PMC152130

[6] Taddeo B, Zhang W, Lakeman F, Roizman B. Cells lacking NF-kappaB or in which NF-kappaB is not activated vary with respect to ability to sustain herpes simplex virus 1 replication and are not susceptible to apoptosis induced by a replication-incompetent mutant virus. J Virol. 2004;78(21):11615–21. doi: 10.1128/JVI.78.21.11615-11621.2004 15479802 PMC523294

[7] Hargett D, Rice S, Bachenheimer SL. Herpes simplex virus type 1 ICP27-dependent activation of NF-kappaB. J Virol. 2006;80(21):10565–78. doi: 10.1128/JVI.01119-06 16928747 PMC1641752

[8] Aktar S, Arii J, Tjan LH, Nishimura M, Mori Y. Human herpesvirus 6A tegument protein U14 induces NF-κB signaling by interacting with p65. J Virol. 2021;95(23):e0126921. doi: 10.1128/JVI.01269-21 34549982 PMC8577390

[9] Gianni T, Leoni V, Campadelli-Fiume G. Type I interferon and NF-κB activation elicited by herpes simplex virus gH/gL via αvβ3 integrin in epithelial and neuronal cell lines. J Virol. 2013;87(24):13911–6. doi: 10.1128/JVI.01894-13 24109241 PMC3838217

[10] Tanaka T, Grusby MJ, Kaisho T. PDLIM2-mediated termination of transcription factor NF-kappaB activation by intranuclear sequestration and degradation of the p65 subunit. Nat Immunol. 2007;8(6):584–91. doi: 10.1038/ni1464 17468759

[11] Amici C, Belardo G, Rossi A, Santoro MG. Activation of I kappa b kinase by herpes simplex virus type 1. A novel target for anti-herpetic therapy. J Biol Chem. 2001;276(31):28759–66. doi: 10.1074/jbc.M103408200 11387335

[12] Yurochko AD, Hwang ES, Rasmussen L, Keay S, Pereira L, Huang ES. The human cytomegalovirus UL55 (gB) and UL75 (gH) glycoprotein ligands initiate the rapid activation of Sp1 and NF-kappaB during infection. J Virol. 1997;71(7):5051–9. doi: 10.1128/JVI.71.7.5051-5059.1997 9188570 PMC191738

[13] Liu X, Fitzgerald K, Kurt-Jones E, Finberg R, Knipe DM. Herpesvirus tegument protein activates NF-kappaB signaling through the TRAF6 adaptor protein. Proc Natl Acad Sci U S A. 2008;105(32):11335–9. doi: 10.1073/pnas.0801617105 18682563 PMC2516262

[14] Leban J, Baierl M, Mies J, Trentinaglia V, Rath S, Kronthaler K, et al. A novel class of potent NF-kappaB signaling inhibitors. Bioorg Med Chem Lett. 2007;17(21):5858–62. doi: 10.1016/j.bmcl.2007.08.022 17869512

[15] Ehrhardt C, Rückle A, Hrincius ER, Haasbach E, Anhlan D, Ahmann K, et al. The NF-κB inhibitor SC75741 efficiently blocks influenza virus propagation and confers a high barrier for development of viral resistance. Cell Microbiol. 2013;15(7):1198–211. doi: 10.1111/cmi.12108 23320394

[16] Tobe M, Isobe Y, Tomizawa H, Nagasaki T, Takahashi H, Fukazawa T, et al. Discovery of quinazolines as a novel structural class of potent inhibitors of NF-kappa B activation. Bioorg Med Chem. 2003;11(3):383–91. doi: 10.1016/s0968-0896(02)00440-6 12517433

[17] Tobe M, Isobe Y, Tomizawa H, Nagasaki T, Takahashi H, Hayashi H. A novel structural class of potent inhibitors of NF-kappa B activation: structure-activity relationships and biological effects of 6-aminoquinazoline derivatives. Bioorg Med Chem. 2003;11(18):3869–78. doi: 10.1016/s0968-0896(03)00438-3 12927847

[18] Jiang X, Chen ZJ. The role of ubiquitylation in immune defence and pathogen evasion. Nat Rev Immunol. 2011;12(1):35–48. doi: 10.1038/nri3111 22158412 PMC3864900

[19] Bhat SA, Vasi Z, Adhikari R, Gudur A, Ali A, Jiang L, et al. Ubiquitin proteasome system in immune regulation and therapeutics. Curr Opin Pharmacol. 2022;67:102310. doi: 10.1016/j.coph.2022.102310 36288660 PMC10163937

[20] Jodo A, Shibazaki A, Onuma A, Kaisho T, Tanaka T. PDLIM7 synergizes with PDLIM2 and p62/Sqstm1 to inhibit inflammatory signaling by promoting degradation of the p65 subunit of NF-κB. Front Immunol. 2020;11:1559. doi: 10.3389/fimmu.2020.01559 32849529 PMC7417631

[21] Hennig T, Prusty AB, Kaufer BB, Whisnant AW, Lodha M, Enders A, et al. Selective inhibition of miRNA processing by a herpesvirus-encoded miRNA. Nature. 2022;605(7910):539–44. doi: 10.1038/s41586-022-04667-4 35508655

[22] Amaliin K, Gulijiahani A, Hirai M, Mori Y, Arii J. Conserved U14-mediated NF-kappaB activation in human herpesvirus 6A and 6B. Microbiol Immunol. 2026.doi: 10.1111/1348-0421.70059 42015664

[23] Zeman MK, Cimprich KA. Causes and consequences of replication stress. Nat Cell Biol. 2014;16(1):2–9. doi: 10.1038/ncb2897 24366029 PMC4354890

[24] Branzei D, Foiani M. Maintaining genome stability at the replication fork. Nat Rev Mol Cell Biol. 2010;11(3):208–19. doi: 10.1038/nrm2852 20177396

[25] Lukas C, Savic V, Bekker-Jensen S, Doil C, Neumann B, Pedersen RS, et al. 53BP1 nuclear bodies form around DNA lesions generated by mitotic transmission of chromosomes under replication stress. Nat Cell Biol. 2011;13(3):243–53. doi: 10.1038/ncb2201 21317883

[26] Mori J, Kawabata A, Tang H, Tadagaki K, Mizuguchi H, Kuroda K, et al. Human Herpesvirus-6 U14 induces cell-cycle arrest in G2/M phase by associating with a cellular protein, EDD. PLoS One. 2015;10(9):e0137420. doi: 10.1371/journal.pone.0137420 26340541 PMC4560387

[27] Ishov AM, Vladimirova OV, Maul GG. Daxx-mediated accumulation of human cytomegalovirus tegument protein pp71 at ND10 facilitates initiation of viral infection at these nuclear domains. J Virol. 2002;76(15):7705–12. doi: 10.1128/jvi.76.15.7705-7712.2002 12097584 PMC136388

[28] Tomtishen JP 3rd. Human cytomegalovirus tegument proteins (pp65, pp71, pp150, pp28). Virol J. 2012;9:22. doi: 10.1186/1743-422X-9-22 22251420 PMC3278345

[29] Kalejta RF, Albright ER. Expanding the known functional repertoire of the human cytomegalovirus pp71 protein. Front Cell Infect Microbiol. 2020;10:95. doi: 10.3389/fcimb.2020.00095 32226778 PMC7080695

[30] Wysocka J, Herr W. The herpes simplex virus VP16-induced complex: the makings of a regulatory switch. Trends Biochem Sci. 2003;28(6):294–304. doi: 10.1016/S0968-0004(03)00088-4 12826401

[31] Mossman KL, Sherburne R, Lavery C, Duncan J, Smiley JR. Evidence that herpes simplex virus VP16 is required for viral egress downstream of the initial envelopment event. J Virol. 2000;74(14):6287–99. doi: 10.1128/jvi.74.14.6287-6299.2000 10864638 PMC112134

[32] La Boissière S, Izeta A, Malcomber S, O’Hare P. Compartmentalization of VP16 in cells infected with recombinant herpes simplex virus expressing VP16-green fluorescent protein fusion proteins. J Virol. 2004;78(15):8002–14. doi: 10.1128/JVI.78.15.8002-8014.2004 15254172 PMC446094

[33] Luftig MA. Viruses and the DNA damage response: activation and antagonism. Annu Rev Virol. 2014;1(1):605–25. doi: 10.1146/annurev-virology-031413-085548 26958736

[34] Full F, Ensser A. Early nuclear events after herpesviral infection. J Clin Med. 2019;8(9):1408. doi: 10.3390/jcm8091408 31500286 PMC6780142

[35] Mertens ME, Knipe DM. Herpes simplex virus 1 manipulates host cell antiviral and proviral DNA damage responses. mBio. 2021;12(1):e03552-20. doi: 10.1128/mBio.03552-20 33563816 PMC7885110

[36] Arii J, Shindo K, Koyanagi N, Kato A, Kawaguchi Y. Multiple roles of the cytoplasmic domain of herpes simplex virus 1 envelope glycoprotein D in infected cells. J Virol. 2016;90(22):10170–81. doi: 10.1128/JVI.01396-16 27581980 PMC5105654

[37] Arii J, Watanabe M, Maeda F, Tokai-Nishizumi N, Chihara T, Miura M, et al. ESCRT-III mediates budding across the inner nuclear membrane and regulates its integrity. Nat Commun. 2018;9(1):3379. doi: 10.1038/s41467-018-05889-9 30139939 PMC6107581

[38] Arii J, Takeshima K, Maruzuru Y, Koyanagi N, Nakayama Y, Kato A, et al. Role of the arginine cluster in the disordered domain of herpes simplex virus 1 UL34 for the recruitment of ESCRT-III for viral primary envelopment. J Virol. 2022;96(2):e0170421. doi: 10.1128/JVI.01704-21 34730397 PMC8791252

[39] Aktar S, Arii J, Nguyen TTH, Huang JR, Nishimura M, Mori Y. ATF1 restricts human herpesvirus 6A replication via beta interferon induction. J Virol. 2022;96(19):e0126422. doi: 10.1128/jvi.01264-22 36154610 PMC9555150

[40] Arii J, Aktar S, Huang JR, Hirai M, Kawamura Y, Miura H, et al. A viral APOBEC3 antagonist distinguishes HHV-6A from HHV-6B. Nat Commun. 2026;17(1):3566. doi: 10.1038/s41467-026-71951-6 42067535 PMC13134964

[41] Huang JR, Arii J, Hirai M, Nishimura M, Mori Y. Human herpesvirus 6A nuclear matrix protein U37 interacts with heat shock transcription factor 1 and activates the heat shock response. J Virol. 2023;97(9):e0071823. doi: 10.1128/jvi.00718-23 37671864 PMC10537701

[42] Gulijiahani A, Arii J, Isakovic V, Huang JR, Kawaguchi Y, Mori Y. ESCRT-III is recruited by human herpesvirus 6A nuclear egress complex to promote nuclear egress of the nucleocapsid. J Virol. 2025;99(9):e0084425. doi: 10.1128/jvi.00844-25 40827914 PMC12456135

[43] Gulijiahani A, Isakovic V, Mori Y, Arii J. Comparative analysis of the nuclear egress complex in human herpesvirus 6A and 6B. Microbiol Immunol. 2025;69(10):502–10. doi: 10.1111/1348-0421.70004 40715028

[44] Chu D, Nguyen A, Smith SS, Vavrušová Z, Schneider RA. Stable integration of an optimized inducible promoter system enables spatiotemporal control of gene expression throughout avian development. Biol Open. 2020;9(10):bio055343. doi: 10.1242/bio.055343 32917762 PMC7561481

[45] Thakore PI, D’Ippolito AM, Song L, Safi A, Shivakumar NK, Kabadi AM, et al. Highly specific epigenome editing by CRISPR-Cas9 repressors for silencing of distal regulatory elements. Nat Methods. 2015;12(12):1143–9. doi: 10.1038/nmeth.3630 26501517 PMC4666778

[46] Stewart SA, Dykxhoorn DM, Palliser D, Mizuno H, Yu EY, An DS, et al. Lentivirus-delivered stable gene silencing by RNAi in primary cells. RNA. 2003;9(4):493–501. doi: 10.1261/rna.2192803 12649500 PMC1370415

[47] Arii J, Fukui A, Shimanaka Y, Kono N, Arai H, Maruzuru Y, et al. Role of phosphatidylethanolamine biosynthesis in herpes simplex virus 1-infected cells in progeny virus morphogenesis in the cytoplasm and in viral pathogenicity in vivo. J Virol. 2020;94(24):e01572-20. doi: 10.1128/JVI.01572-20 32999028 PMC7925202

[48] Arii J, Goto H, Suenaga T, Oyama M, Kozuka-Hata H, Imai T, et al. Non-muscle myosin IIA is a functional entry receptor for herpes simplex virus-1. Nature. 2010;467(7317):859–62. doi: 10.1038/nature09420 20944748

[49] Arii J, Maeda F, Maruzuru Y, Koyanagi N, Kato A, Mori Y, et al. ESCRT-III controls nuclear envelope deformation induced by progerin. Sci Rep. 2020;10(1):18877. doi: 10.1038/s41598-020-75852-6 33139753 PMC7606583

[50] Maeda F, Arii J, Hirohata Y, Maruzuru Y, Koyanagi N, Kato A, et al. Herpes simplex virus 1 UL34 protein regulates the global architecture of the endoplasmic reticulum in infected cells. J Virol. 2017;91(12):e00271-17. doi: 10.1128/JVI.00271-17 28356536 PMC5446647

[51] Hirai M, Amaliin K, Huang JR, Aktar S, Mori Y, Arii J. HHV-6B ribonucleotide reductase sequesters NF-κB subunit p65 to inhibit innate immune responses. iScience. 2024;28(2):111710. doi: 10.1016/j.isci.2024.111710 39877902 PMC11772975

[52] Aiello FA, D’Archivio L, Attili M, Ferraro E, Macrì E, Mazzocchi R, et al. Neddylation inhibition induces DNA double-strand breaks, hampering tumor growth in vivo, and promotes radiosensitivity in PAX3-FOXO1 rhabdomyosarcoma. Cell Death Discov. 2025;11(1):496. doi: 10.1038/s41420-025-02787-0 41184241 PMC12583468

[53] Nikolova T, Marini F, Kaina B. Genotoxicity testing: comparison of the γH2AX focus assay with the alkaline and neutral comet assays. Mutat Res Genet Toxicol Environ Mutagen. 2017;822:10–8. doi: 10.1016/j.mrgentox.2017.07.004 28844237

[54] Anders S, Huber W. Differential expression analysis for sequence count data. Genome Biol. 2010;11(10):R106. doi: 10.1186/gb-2010-11-10-r106 20979621 PMC3218662

[55] Zhu A, Ibrahim JG, Love MI. Heavy-tailed prior distributions for sequence count data: removing the noise and preserving large differences. Bioinformatics. 2019;35(12):2084–92. doi: 10.1093/bioinformatics/bty895 30395178 PMC6581436

[56] Subramanian A, Tamayo P, Mootha VK, Mukherjee S, Ebert BL, Gillette MA, et al. Gene set enrichment analysis: a knowledge-based approach for interpreting genome-wide expression profiles. Proc Natl Acad Sci U S A. 2005;102(43):15545–50. doi: 10.1073/pnas.0506580102 16199517 PMC1239896

[57] Liberzon A, Birger C, Thorvaldsdóttir H, Ghandi M, Mesirov JP, Tamayo P. The Molecular Signatures Database (MSigDB) hallmark gene set collection. Cell Syst. 2015;1(6):417–25. doi: 10.1016/j.cels.2015.12.004 26771021 PMC4707969

[58] Babicki S, Arndt D, Marcu A, Liang Y, Grant JR, Maciejewski A, et al. Heatmapper: web-enabled heat mapping for all. Nucleic Acids Res. 2016;44(W1):W147-53. doi: 10.1093/nar/gkw419 27190236 PMC4987948

