# Supplementary material for: Host-directed clearance of nuclear tegument U14 terminates NF-κB signaling and supports HHV-6A replication
Source: PLoS Pathog. 2026 Sep 23;22(9):e1014576. doi: 10.1371/journal.ppat.1014576 (PMC13600490; doi:10.1371/journal.ppat.1014576)
Supplement: Supplementary file 1 [file ppat.1014576.s001.pdf]

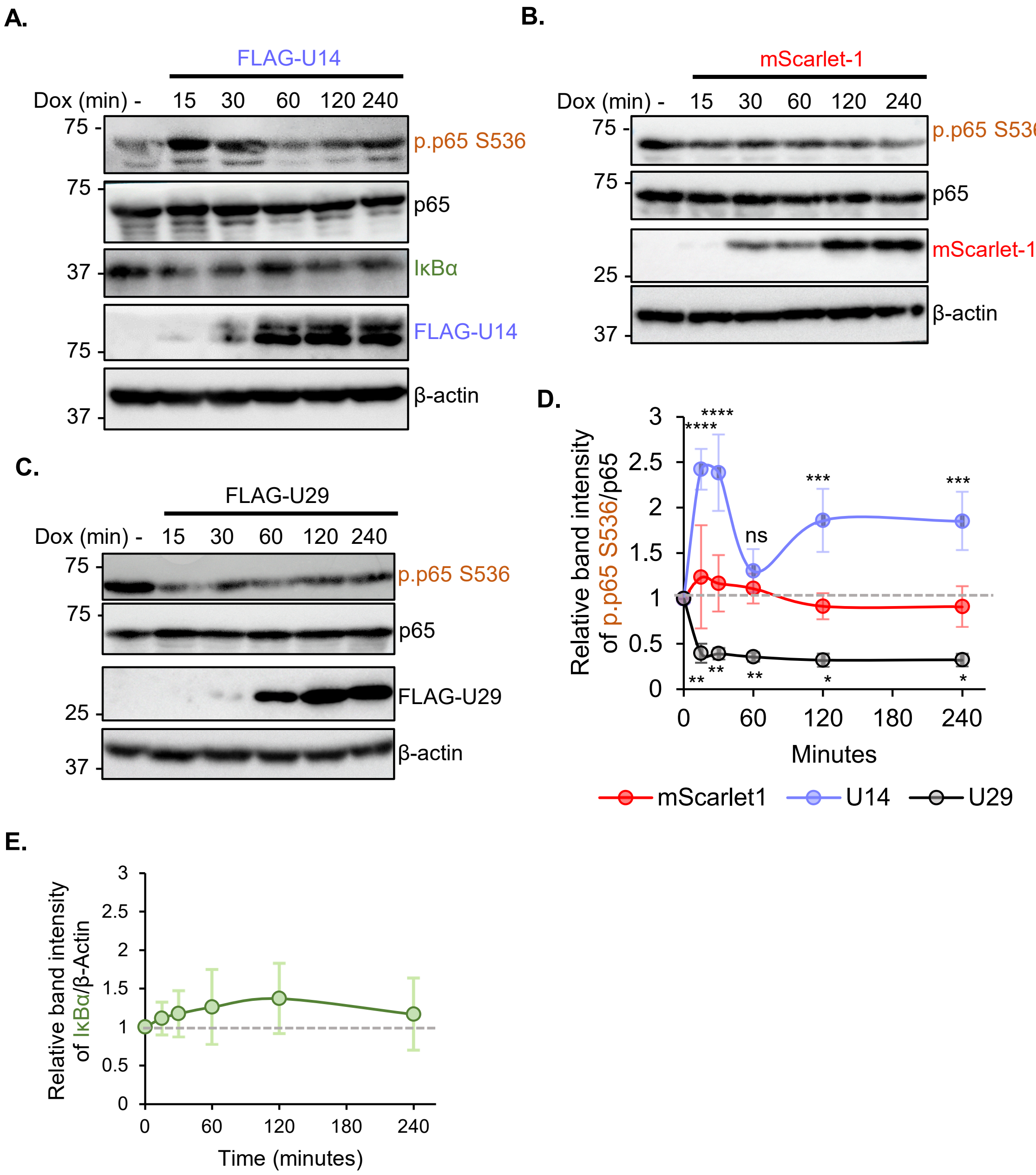
A.
FLAG-U14
Dox (min) - 15 30 60 120 240
75
p.p65 S536
75
p65
37
IκBα
FLAG-U14
75
β-actin
37
B.
mScarlet-1
Dox (min) - 15 30 60 120 240
75
p.p65 S536
75
p65
mScarlet-1
25
β-actin
37
C.
FLAG-U29
Dox (min) - 15 30 60 120 240
75
p.p65 S536
75
p65
FLAG-U29
25
β-actin
37
D.
Relative band intensity of p.p65 S536/p65
3
2.5
2
1.5
1
0.5
0
****
****
ns
***
***
**
**
**
*
*
0 60 120 180 240
Minutes
mScarlet1
U14
U29
E.
Relative band intensity of IκBα/β-Actin
3
2.5
2
1.5
1
0.5
0
0 60 120 180 240
Time (minutes)
