# Supplementary material for: Host-directed clearance of nuclear tegument U14 terminates NF-κB signaling and supports HHV-6A replication
Source: PLoS Pathog. 2026 Sep 23;22(9):e1014576. doi: 10.1371/journal.ppat.1014576 (PMC13600490; doi:10.1371/journal.ppat.1014576)
Supplement: Supplementary file 2 [file ppat.1014576.s002.pdf]

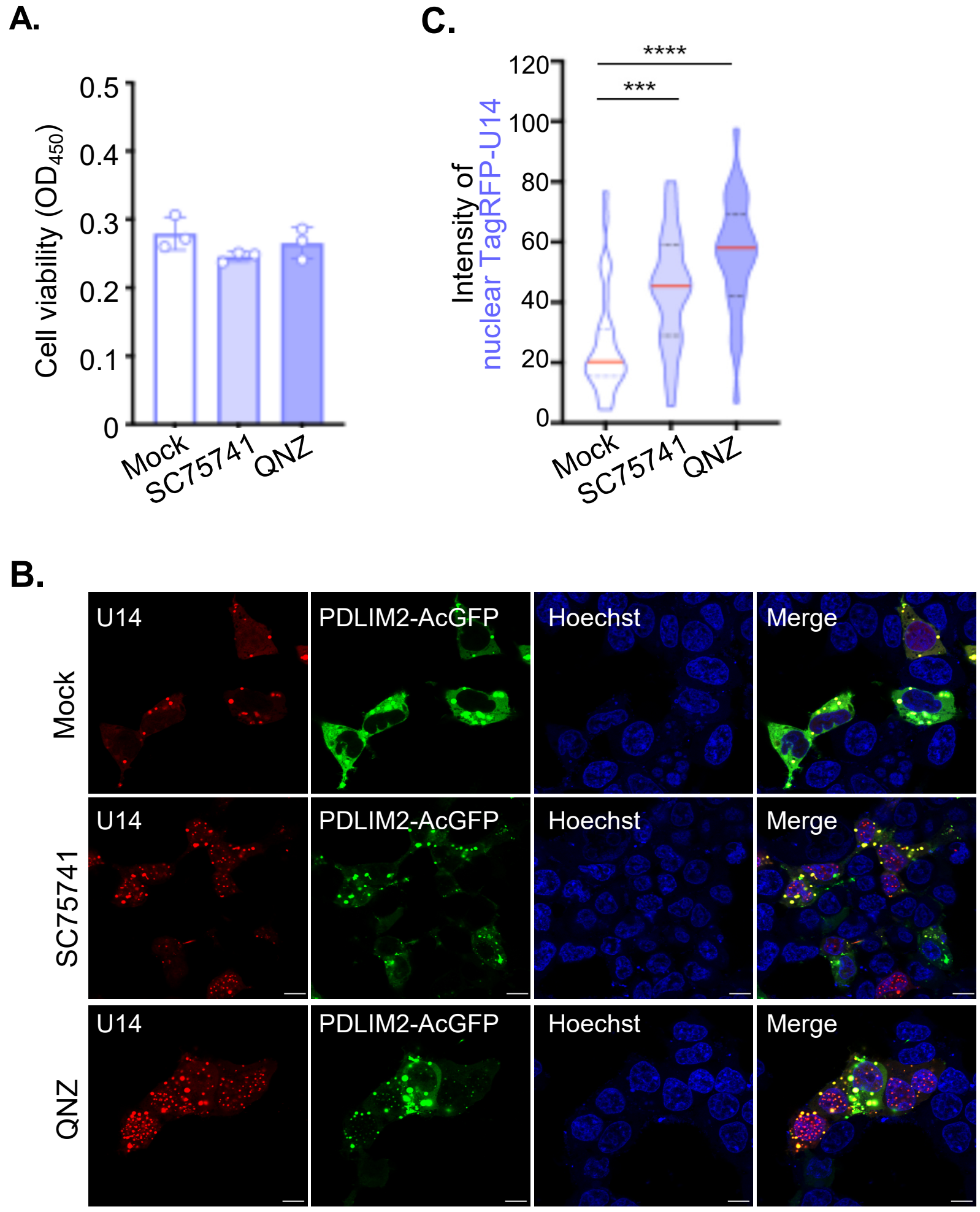
A.
Cell viability (OD_{450})
0
0.1
0.2
0.3
0.4
0.5
Mock
SC75741
QNZ
C.
Intensity of
nuclear TagRFP-U14
0
20
40
60
80
100
120
****
***
Mock
SC75741
QNZ
B.
Mock
SC75741
QNZ
U14
PDLIM2-AcGFP
Hoechst
Merge
