# Supplementary material for: Host-directed clearance of nuclear tegument U14 terminates NF-κB signaling and supports HHV-6A replication
Source: PLoS Pathog. 2026 Sep 23;22(9):e1014576. doi: 10.1371/journal.ppat.1014576 (PMC13600490; doi:10.1371/journal.ppat.1014576)
Supplement: Supplementary file 3 [file ppat.1014576.s003.pdf]

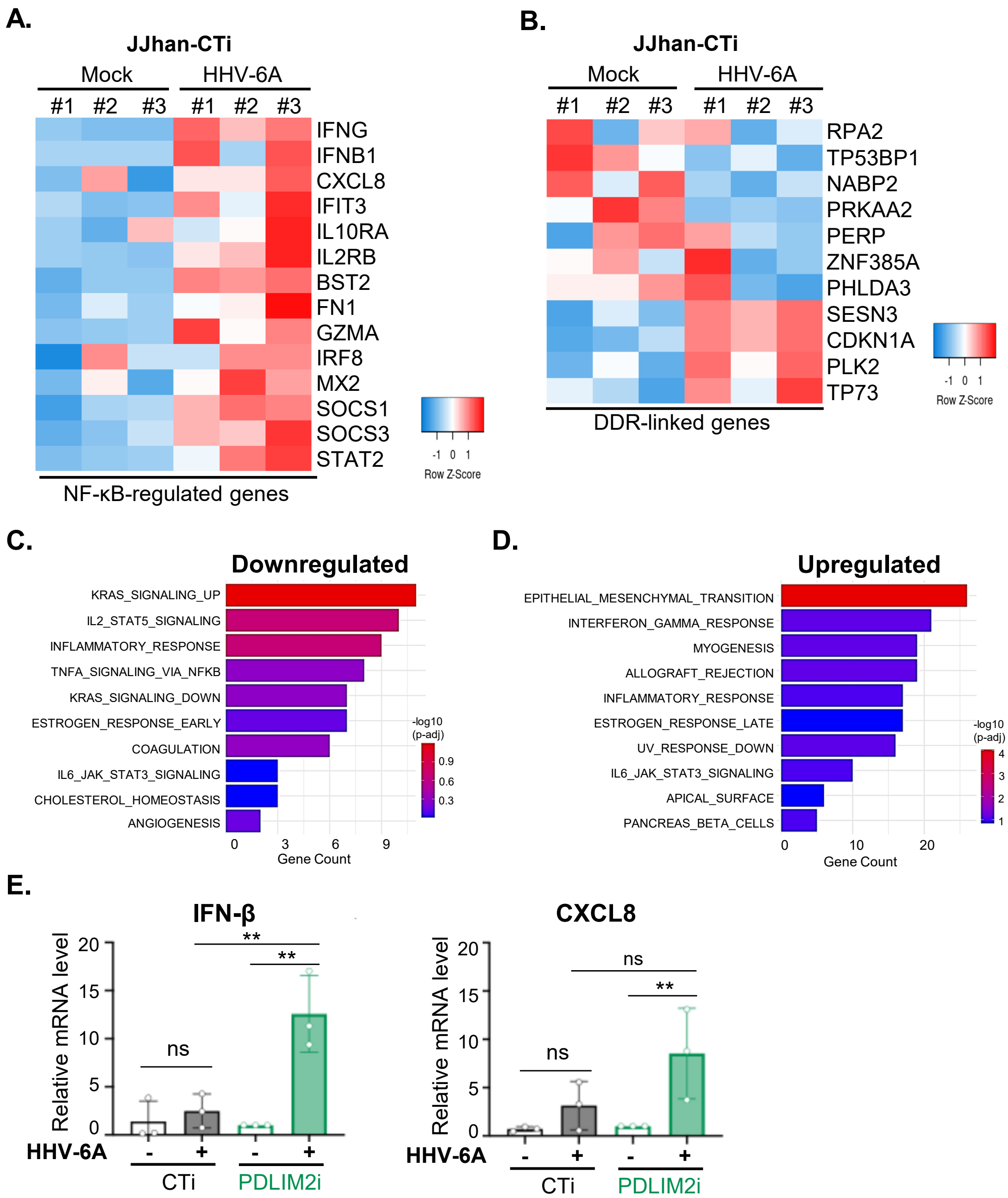
A.
JJhan-CTi
Mock
HHV-6A
#1 #2 #3 #1 #2 #3
IFNG
IFNB1
CXCL8
IFIT3
IL10RA
IL2RB
BST2
FN1
GZMA
IRF8
MX2
SOCS1
SOCS3
STAT2
NF-κB-regulated genes
-1 0 1
Row Z-Score
B.
JJhan-CTi
Mock
HHV-6A
#1 #2 #3 #1 #2 #3
RPA2
TP53BP1
NABP2
PRKAA2
PERP
ZNF385A
PHLDA3
SESN3
CDKN1A
PLK2
TP73
DDR-linked genes
-1 0 1
Row Z-Score
C.
Downregulated
KRAS_SIGNALING_UP
IL2_STAT5_SIGNALING
INFLAMMATORY_RESPONSE
TNFA_SIGNALING_VIA_NFKB
KRAS_SIGNALING_DOWN
ESTROGEN_RESPONSE_EARLY
COAGULATION
IL6_JAK_STAT3_SIGNALING
CHOLESTEROL_HOMEOSTASIS
ANGIOGENESIS
-log10 (p-adj)
0.9
0.6
0.3
0 3 6 9
Gene Count
D.
Upregulated
EPITHELIAL_MESENCHYMAL_TRANSITION
INTERFERON_GAMMA_RESPONSE
MYOGENESIS
ALLOGRAFT_REJECTION
INFLAMMATORY_RESPONSE
ESTROGEN_RESPONSE_LATE
UV_RESPONSE_DOWN
IL6_JAK_STAT3_SIGNALING
APICAL_SURFACE
PANCREAS_BETA_CELLS
-log10 (p-adj)
4
3
2
1
0 10 20
Gene Count
E.
IFN-β
Relative mRNA level
0 5 10 15 20
ns
**
**
HHV-6A - + - +
CTi
PDLIM2i
CXCL8
Relative mRNA level
0 5 10 15 20
ns
ns
**
HHV-6A - + - +
CTi
PDLIM2i
