# Supplementary material for: Host-directed clearance of nuclear tegument U14 terminates NF-κB signaling and supports HHV-6A replication
Source: PLoS Pathog. 2026 Sep 23;22(9):e1014576. doi: 10.1371/journal.ppat.1014576 (PMC13600490; doi:10.1371/journal.ppat.1014576)
Supplement: Supplementary file 4 [file ppat.1014576.s004.pdf]

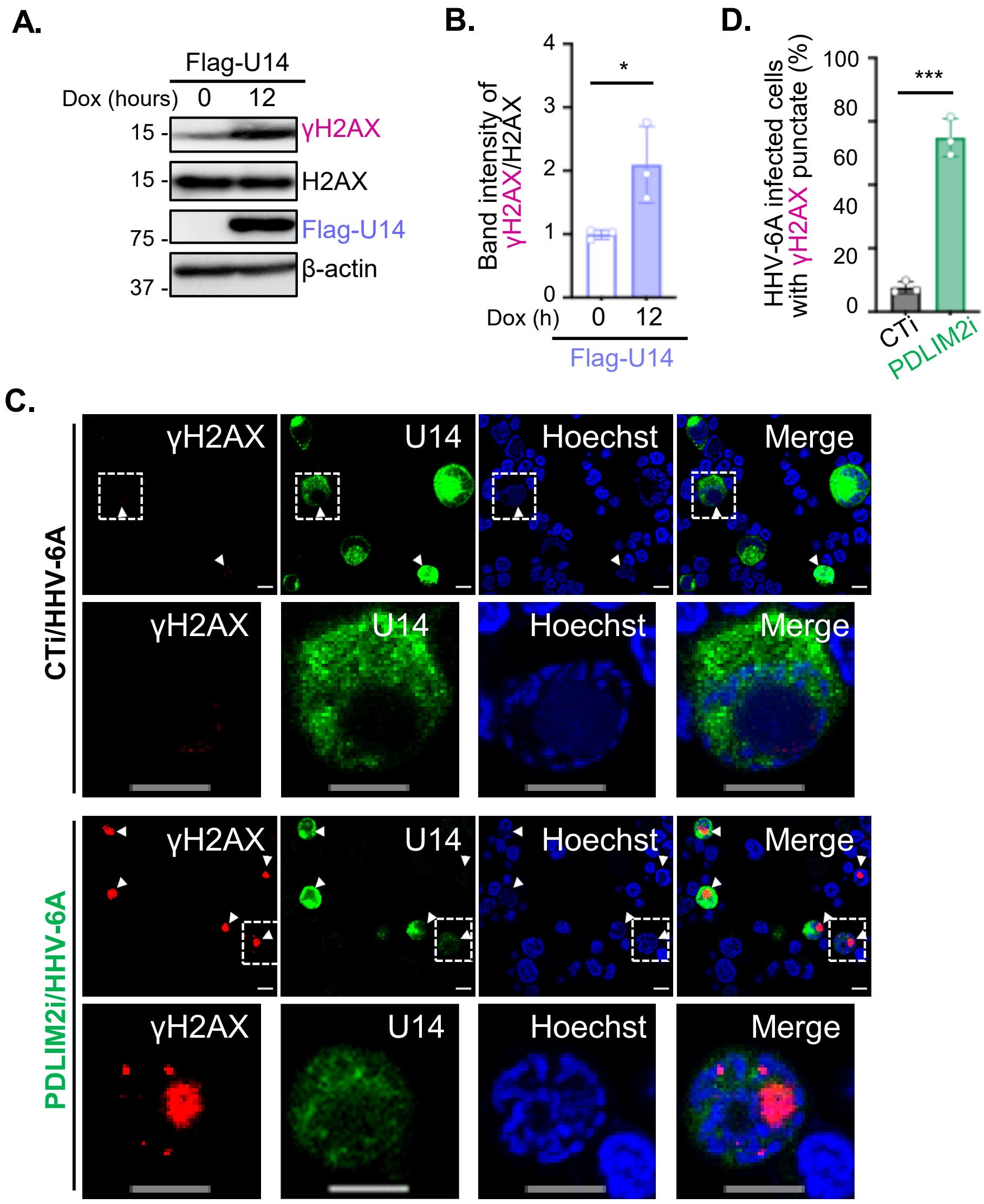
A.
Flag-U14
Dox (hours) 0 12
15
γH2AX
15
H2AX
75
Flag-U14
37
β-actin
B.
Band intensity of γH2AX/H2AX
4
3
2
1
0
*
Dox (h) 0 12
Flag-U14
D.
HHV-6A infected cells with γH2AX punctate (%)
100
80
60
40
20
0
***
CTi
PDLIM2i
C.
CTi/HHV-6A
PDLIM2i/HHV-6A
γH2AX
U14
Hoechst
Merge
