# Supplementary material for: Host-directed clearance of nuclear tegument U14 terminates NF-κB signaling and supports HHV-6A replication
Source: PLoS Pathog. 2026 Sep 23;22(9):e1014576. doi: 10.1371/journal.ppat.1014576 (PMC13600490; doi:10.1371/journal.ppat.1014576)
Supplement: Supplementary file 5 [file ppat.1014576.s005.pdf]

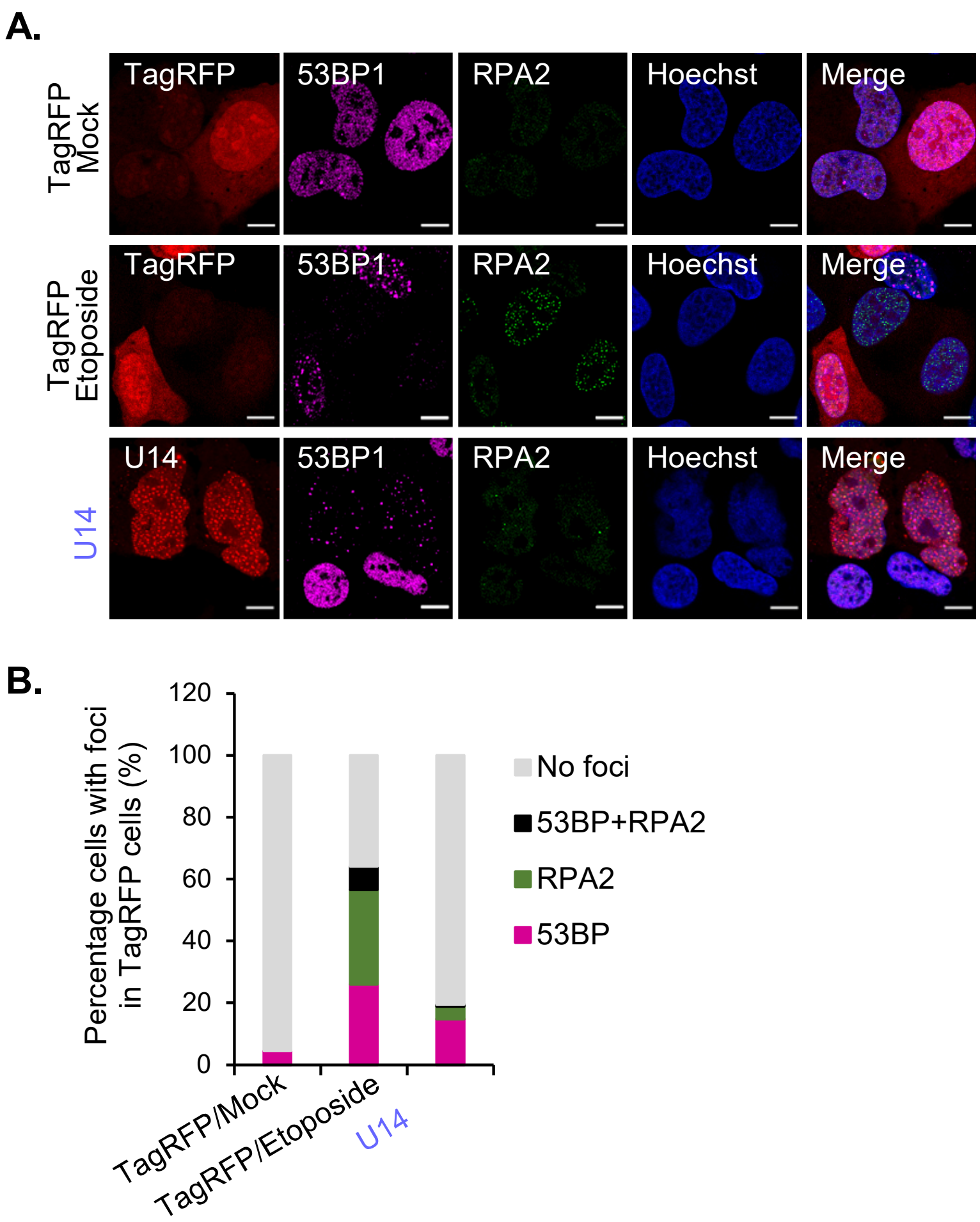
A.
TagRFP
Mock
TagRFP
Etoposide
U14
TagRFP
53BP1
RPA2
Hoechst
Merge
U14
B.
Percentage cells with foci
in TagRFP cells (%)
120
100
80
60
40
20
0
No foci
53BP+RPA2
RPA2
53BP
TagRFP/Mock
TagRFP/Etoposide
U14
