# Supplementary material for: Host-directed clearance of nuclear tegument U14 terminates NF-κB signaling and supports HHV-6A replication
Source: PLoS Pathog. 2026 Sep 23;22(9):e1014576. doi: 10.1371/journal.ppat.1014576 (PMC13600490; doi:10.1371/journal.ppat.1014576)
Supplement: Supplementary file 6 [file ppat.1014576.s006.pdf]

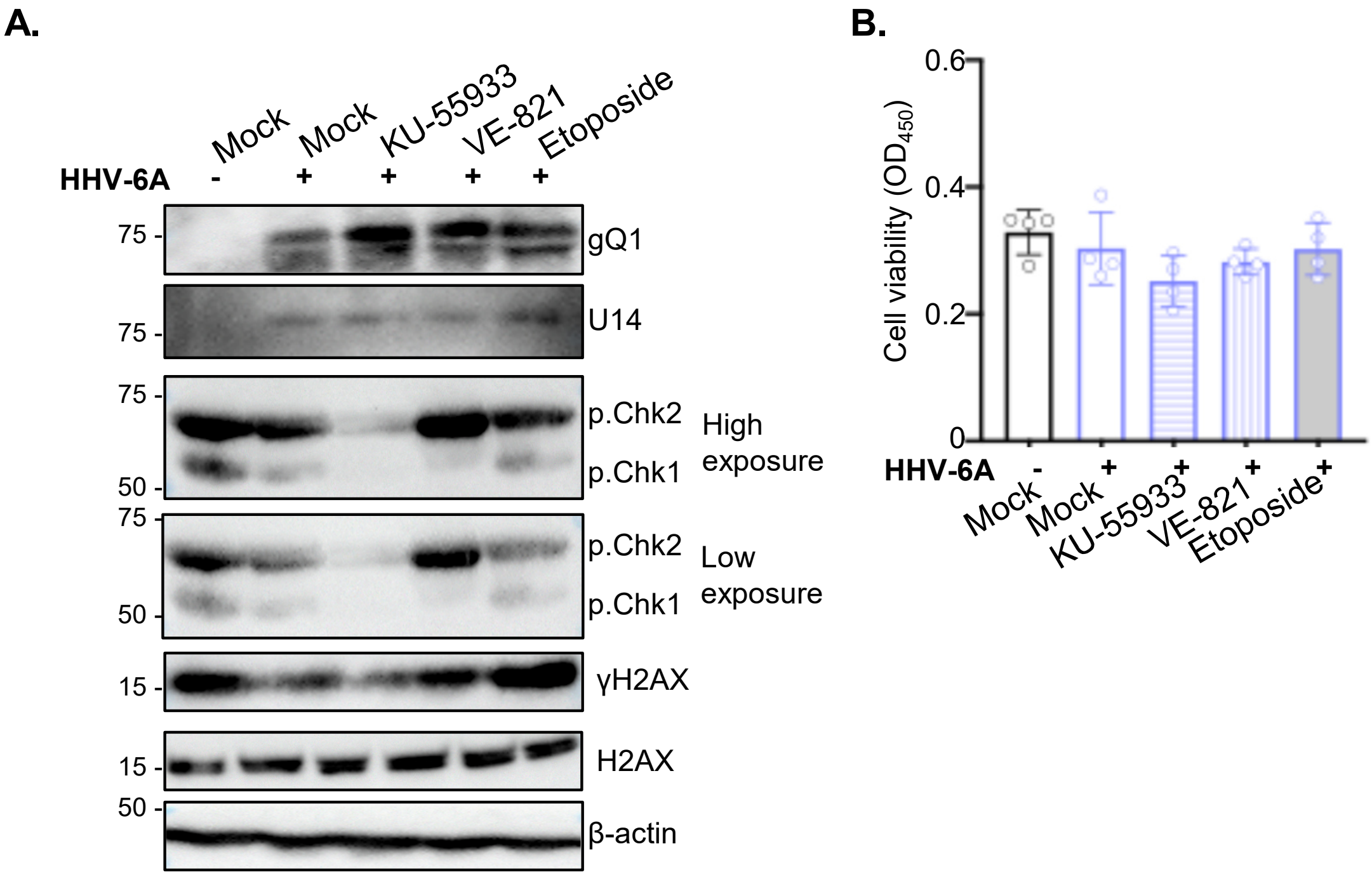
A.
Mock
Mock
KU-55933
VE-821
Etoposide
HHV-6A - + + + +
75
gQ1
75
U14
75
p.Chk2
p.Chk1
50
High exposure
75
p.Chk2
p.Chk1
50
Low exposure
15
γH2AX
15
H2AX
50
β-actin
B.
Cell viability (OD450)
0.6
0.4
0.2
0
HHV-6A - + + + +
Mock
Mock
KU-55933
VE-821
Etoposide
