# Supplementary material for: Host-directed clearance of nuclear tegument U14 terminates NF-κB signaling and supports HHV-6A replication
Source: PLoS Pathog. 2026 Sep 23;22(9):e1014576. doi: 10.1371/journal.ppat.1014576 (PMC13600490; doi:10.1371/journal.ppat.1014576)

Figure 1A

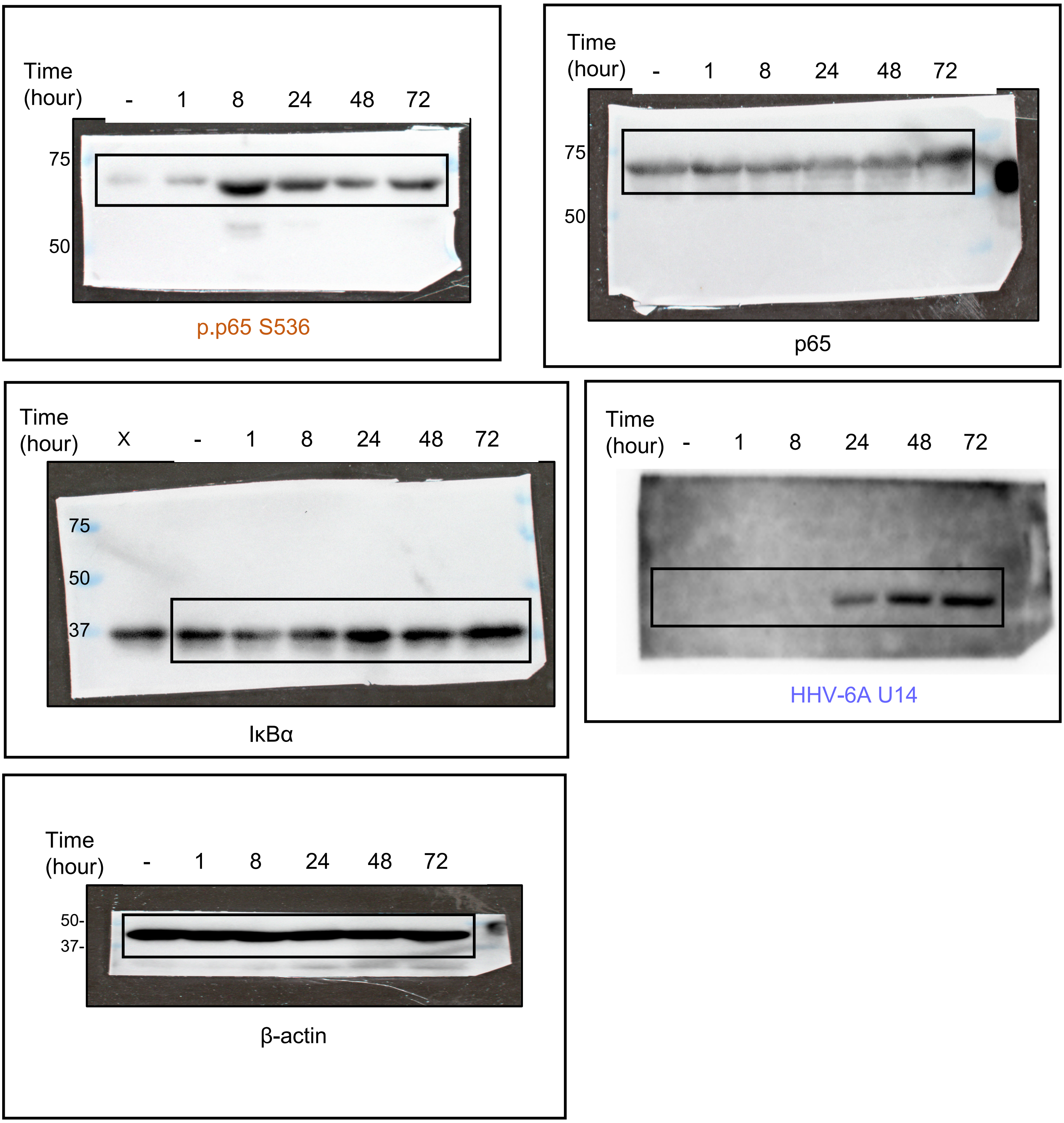

Time (hour) - 1 8 24 48 72
75
50
p.p65 S536
Time (hour) - 1 8 24 48 72
75
50
p65
Time (hour) X - 1 8 24 48 72
75
50
37
IκBα
Time (hour) - 1 8 24 48 72
HHV-6A U14
Time (hour) - 1 8 24 48 72
50-
37-
β-actin

Figure 1D

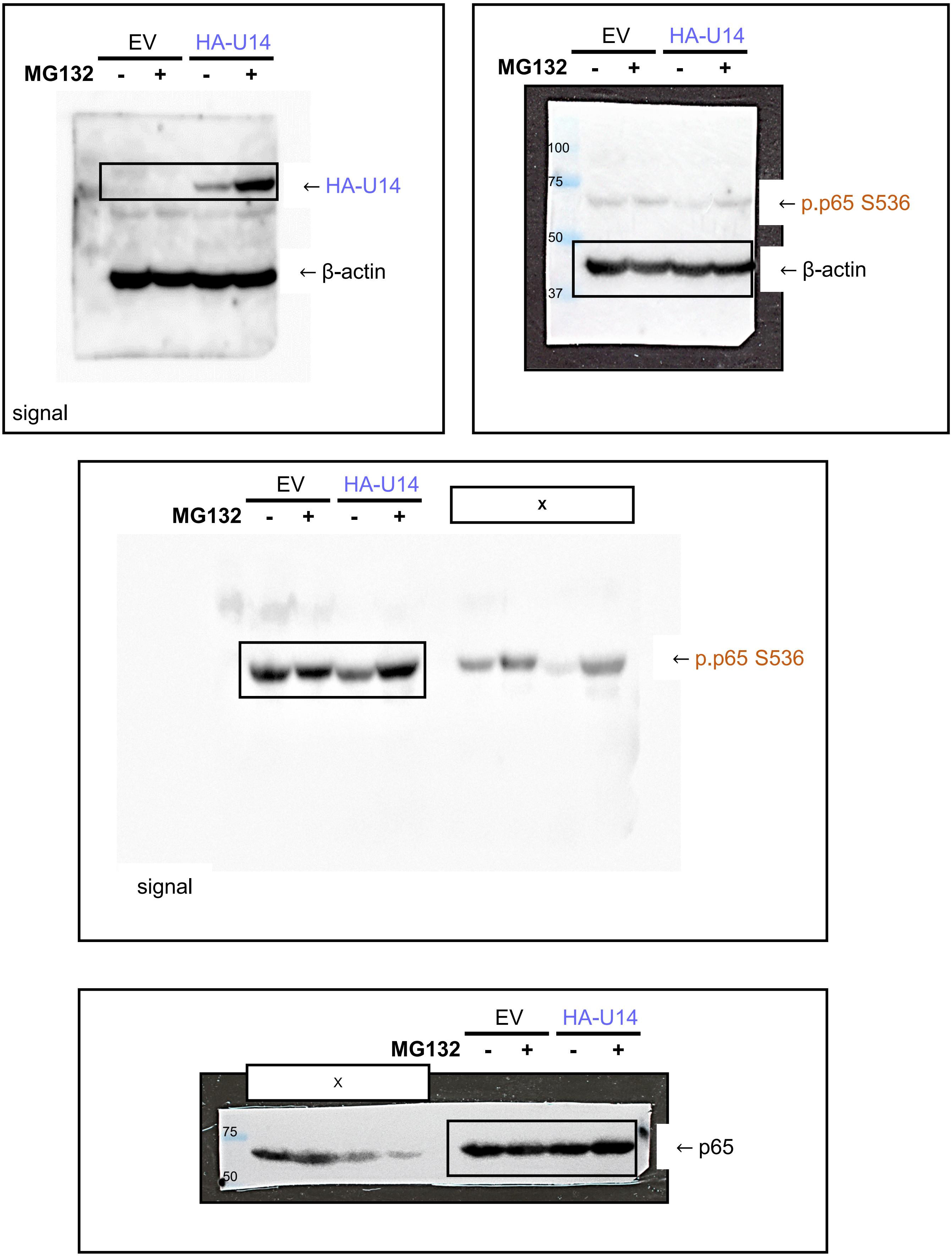
EV
HA-U14
MG132 - + - +
← HA-U14
← β-actin
signal
EV
HA-U14
MG132 - + - +
100
75
50
37
← p.p65 S536
← β-actin
EV
HA-U14
MG132 - + - +
x
← p.p65 S536
signal
EV
HA-U14
MG132 - + - +
x
75
50
← p65

Figure 1G

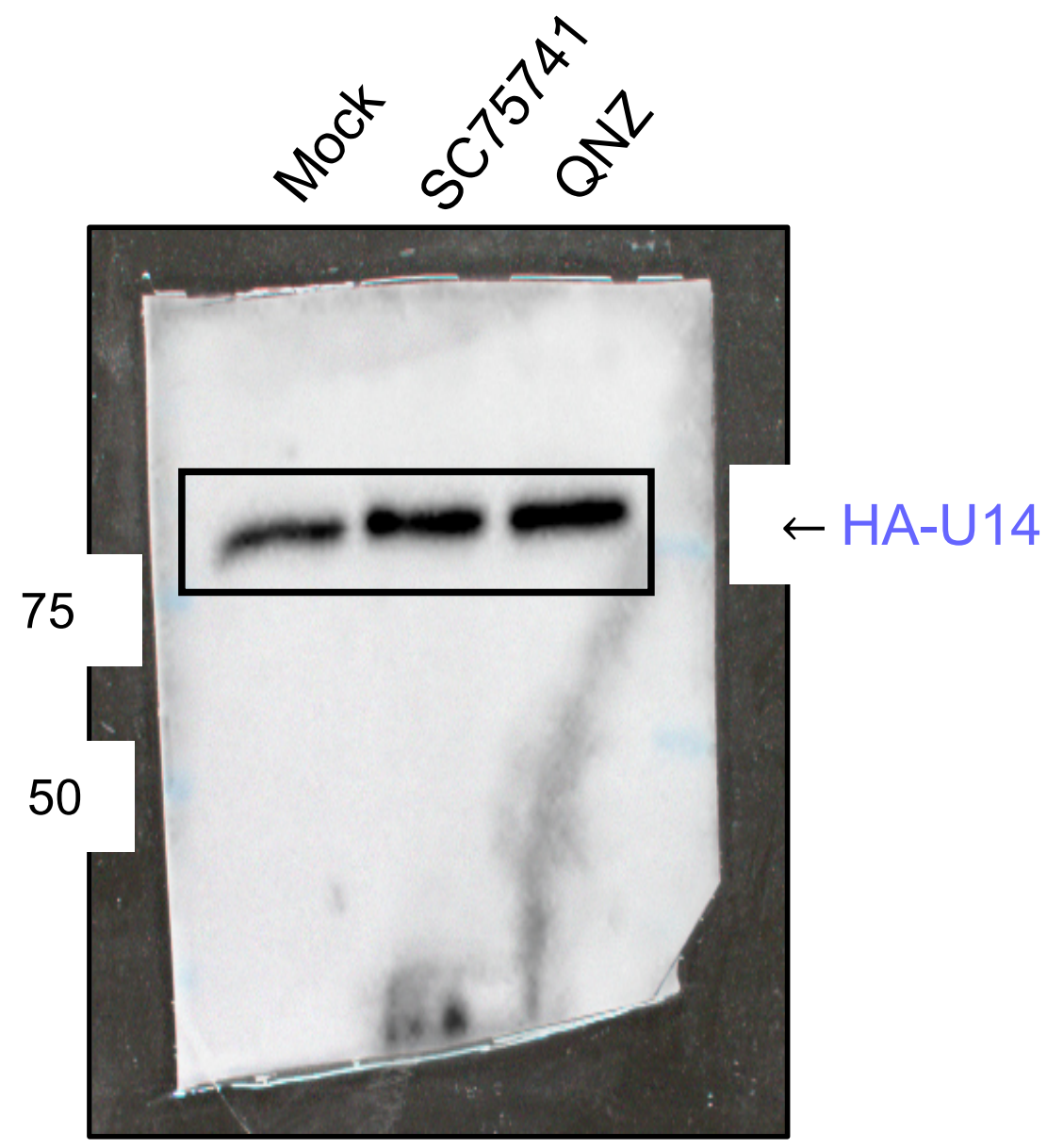

Mock
SC75741
QNZ
75
50
← HA-U14

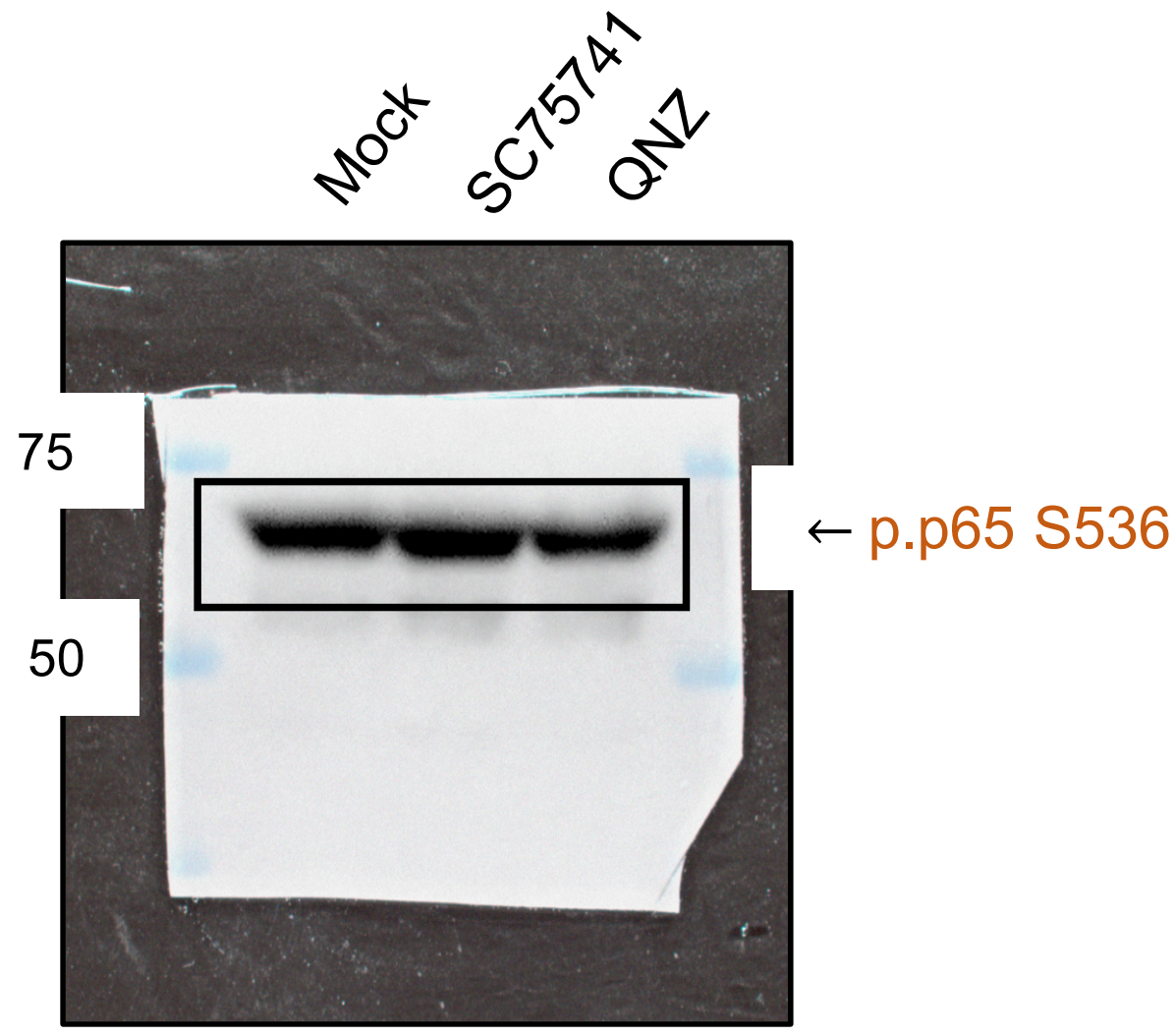

Mock
SC75741
QNZ
75
50
← p.p65 S536

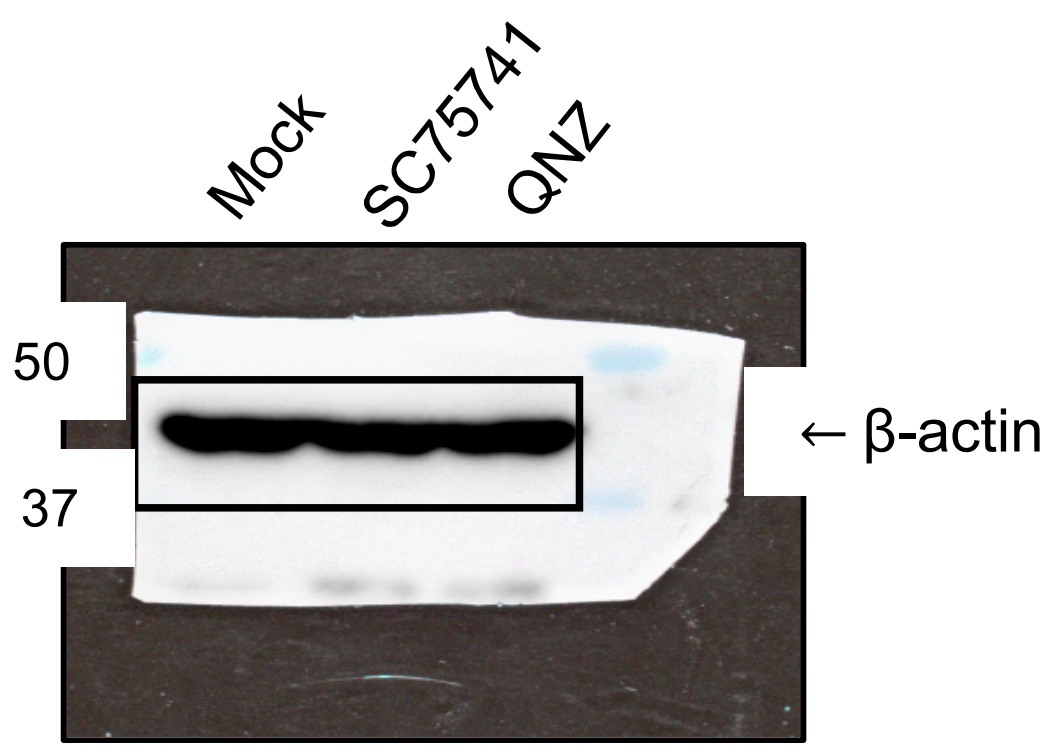

Mock
SC75741
QNZ
50
37
← β-actin

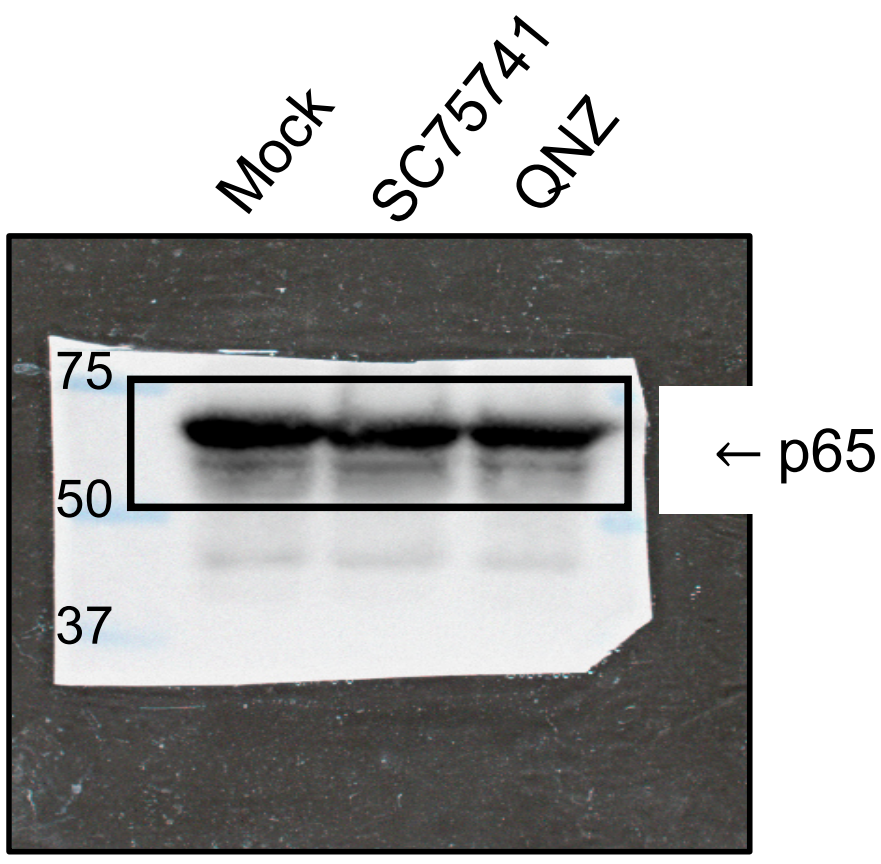

Mock
SC75741
QNZ
75
50
37
← p65

Figure 2B

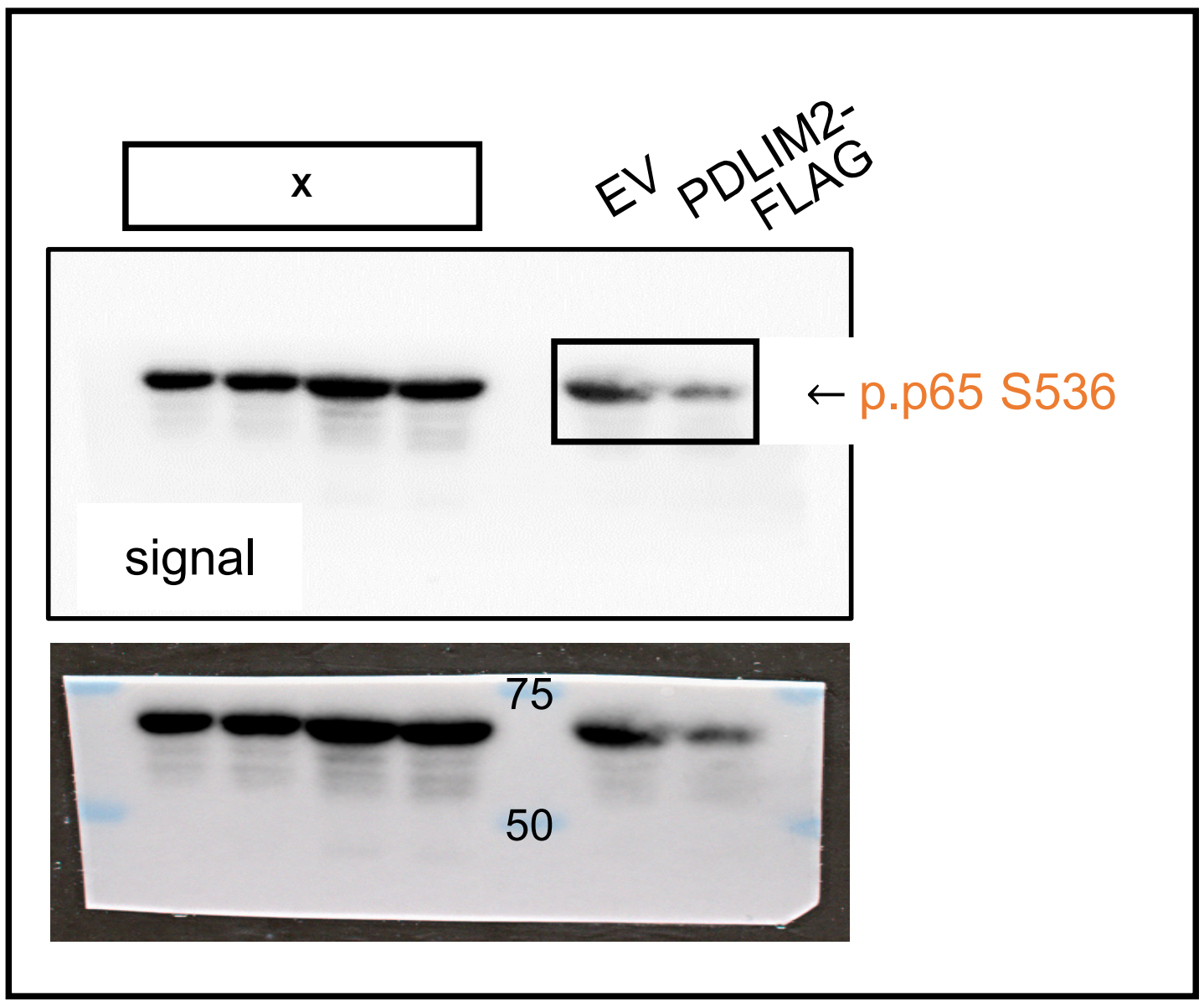

x
EV
PDLIM2-FLAG
← p.p65 S536
signal
75
50

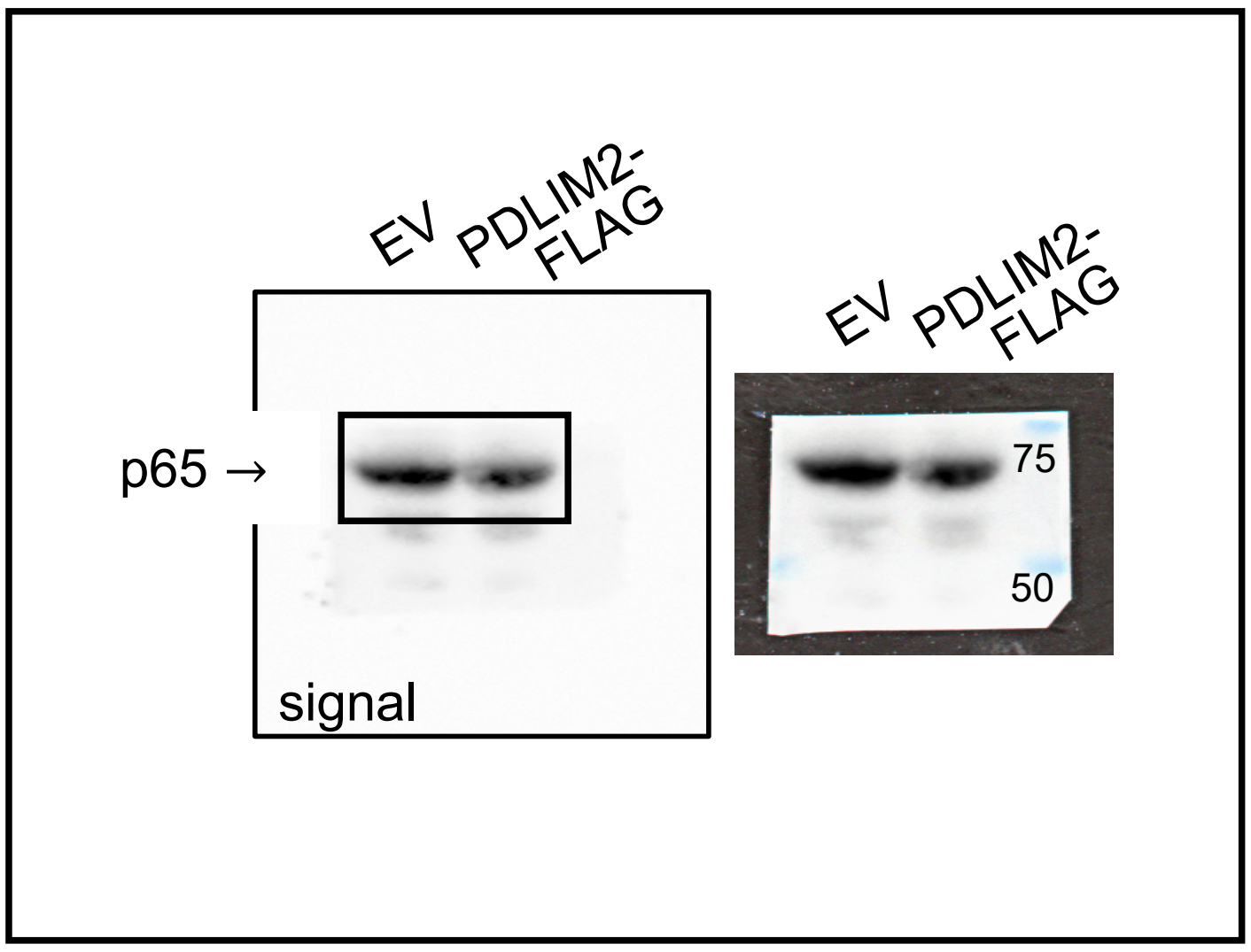

EV
PDLIM2-FLAG
p65 →
signal
EV
PDLIM2-FLAG
75
50

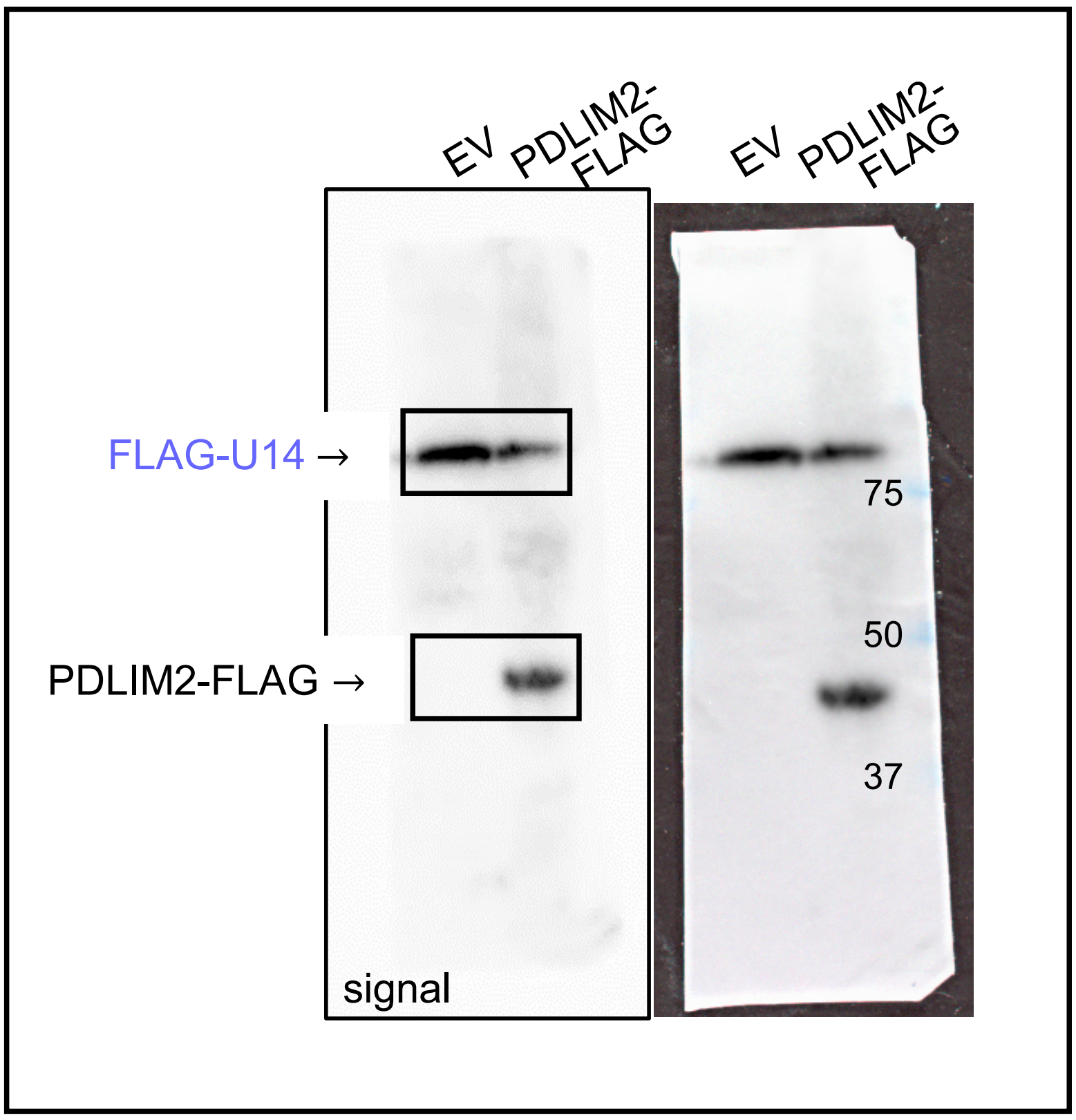

EV
PDLIM2-FLAG
EV
PDLIM2-FLAG
FLAG-U14 →
PDLIM2-FLAG →
signal
75
50
37

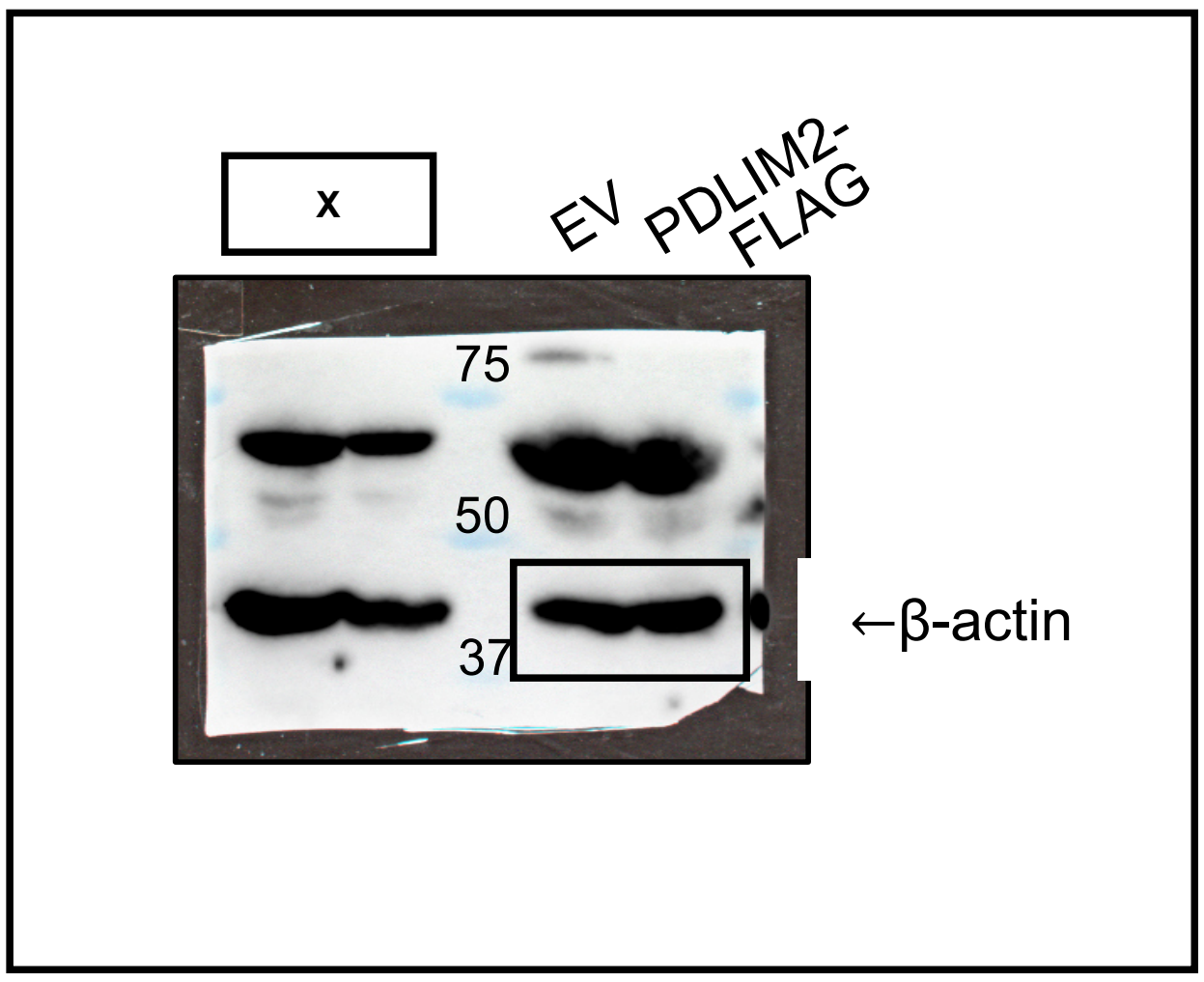

x
EV
PDLIM2-FLAG
75
50
37
←β-actin

Figure 2D

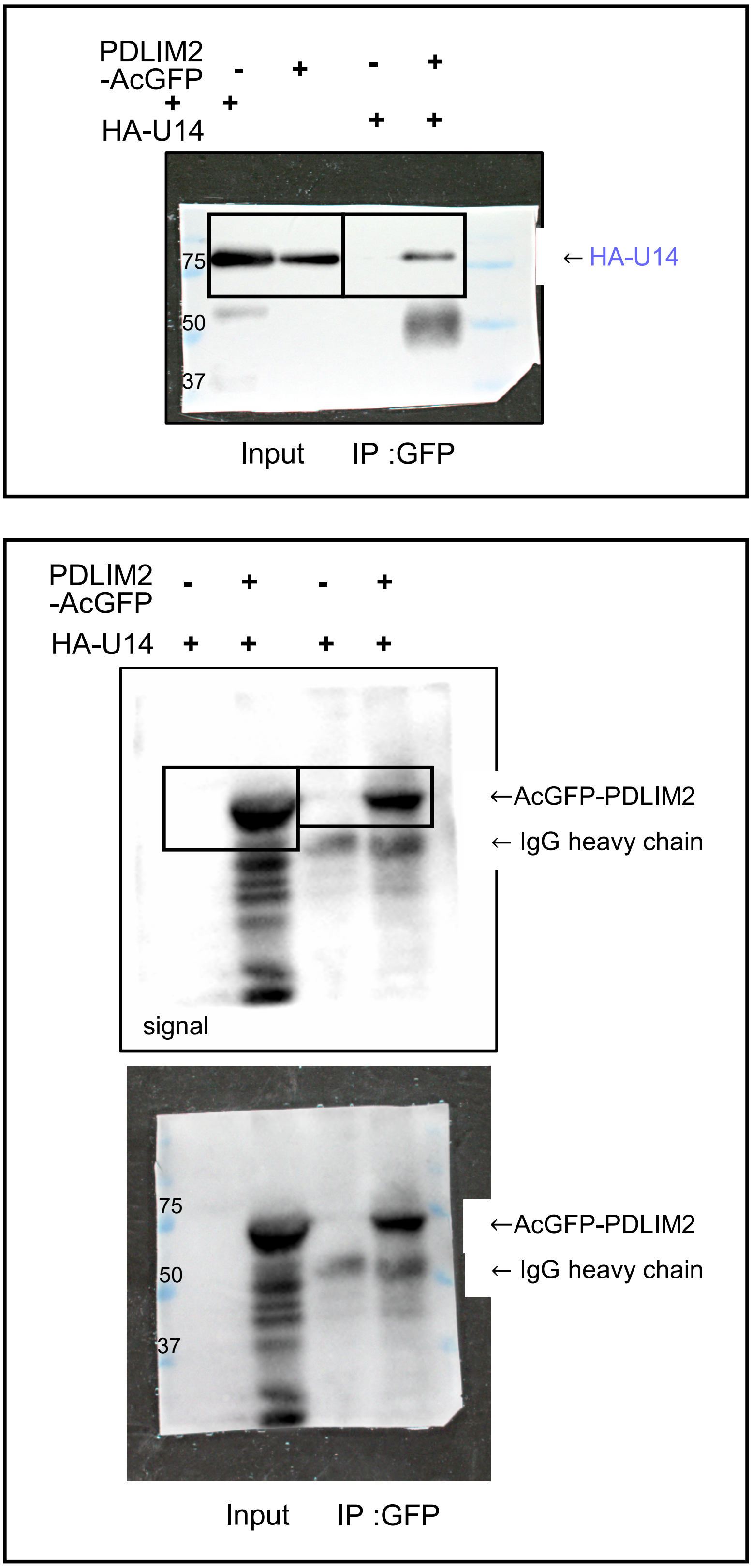
PDLIM2
-AcGFP
- + - +
HA-U14
+ + + +
75
50
37
← HA-U14
Input
IP :GFP
PDLIM2
-AcGFP
- + - +
HA-U14
+ + + +
←AcGFP-PDLIM2
← IgG heavy chain
signal
75
50
37
←AcGFP-PDLIM2
← IgG heavy chain
Input
IP :GFP

Figure 2E

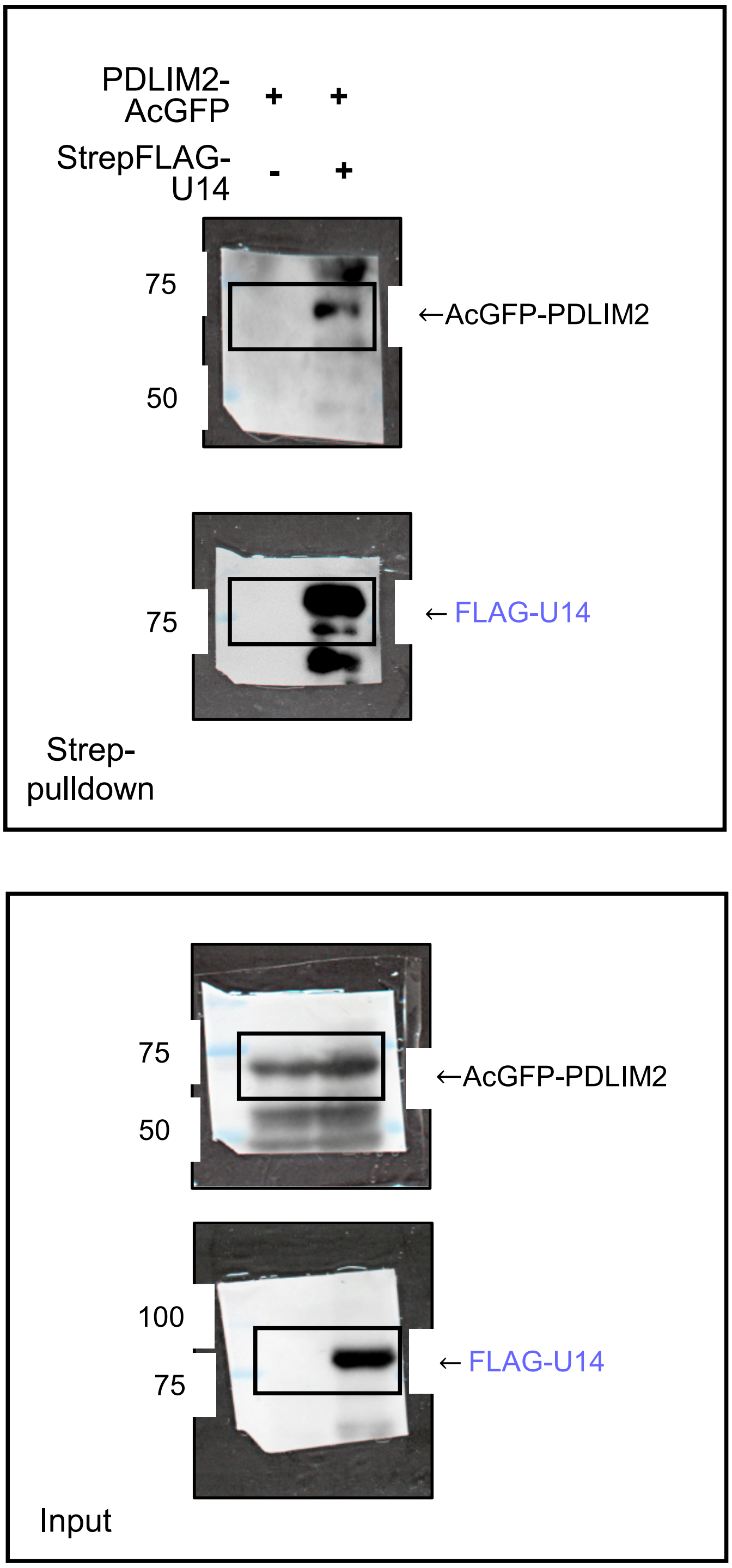

PDLIM2-AcGFP + +
StrepFLAG-U14 - +
75
50
←AcGFP-PDLIM2
75
← FLAG-U14
Strep-pulldown
75
50
←AcGFP-PDLIM2
100
75
← FLAG-U14
Input

Figure 2F

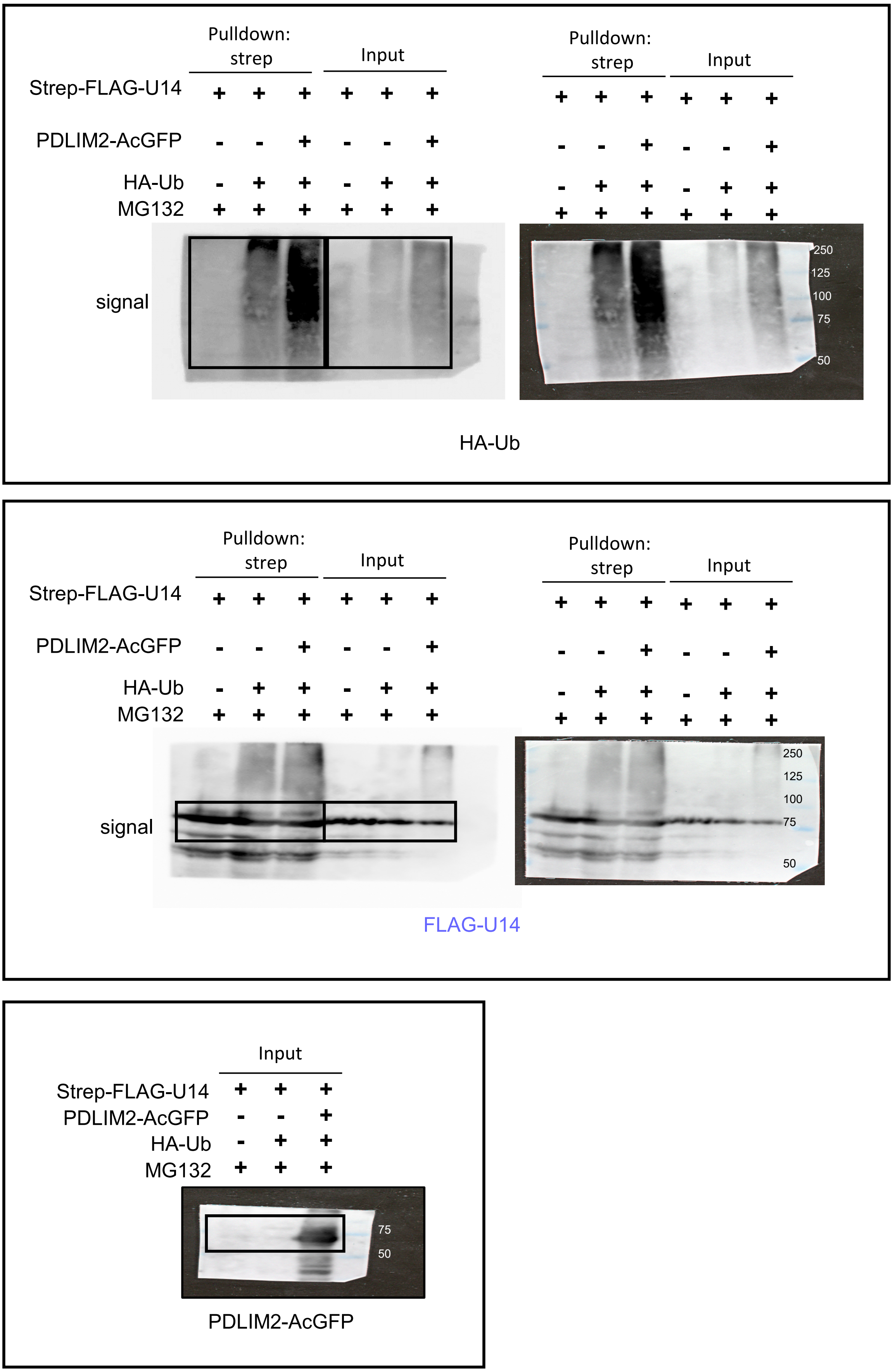
Pulldown: strep
Input
Strep-FLAG-U14 + + + + + +
PDLIM2-AcGFP - - + - - +
HA-Ub - + + - + +
MG132 + + + + + +
signal
250
125
100
75
50
HA-Ub
Pulldown: strep
Input
Strep-FLAG-U14 + + + + + +
PDLIM2-AcGFP - - + - - +
HA-Ub - + + - + +
MG132 + + + + + +
signal
250
125
100
75
50
FLAG-U14
Input
Strep-FLAG-U14 + + +
PDLIM2-AcGFP - - +
HA-Ub - + +
MG132 + + +
75
50
PDLIM2-AcGFP

Figure 3B

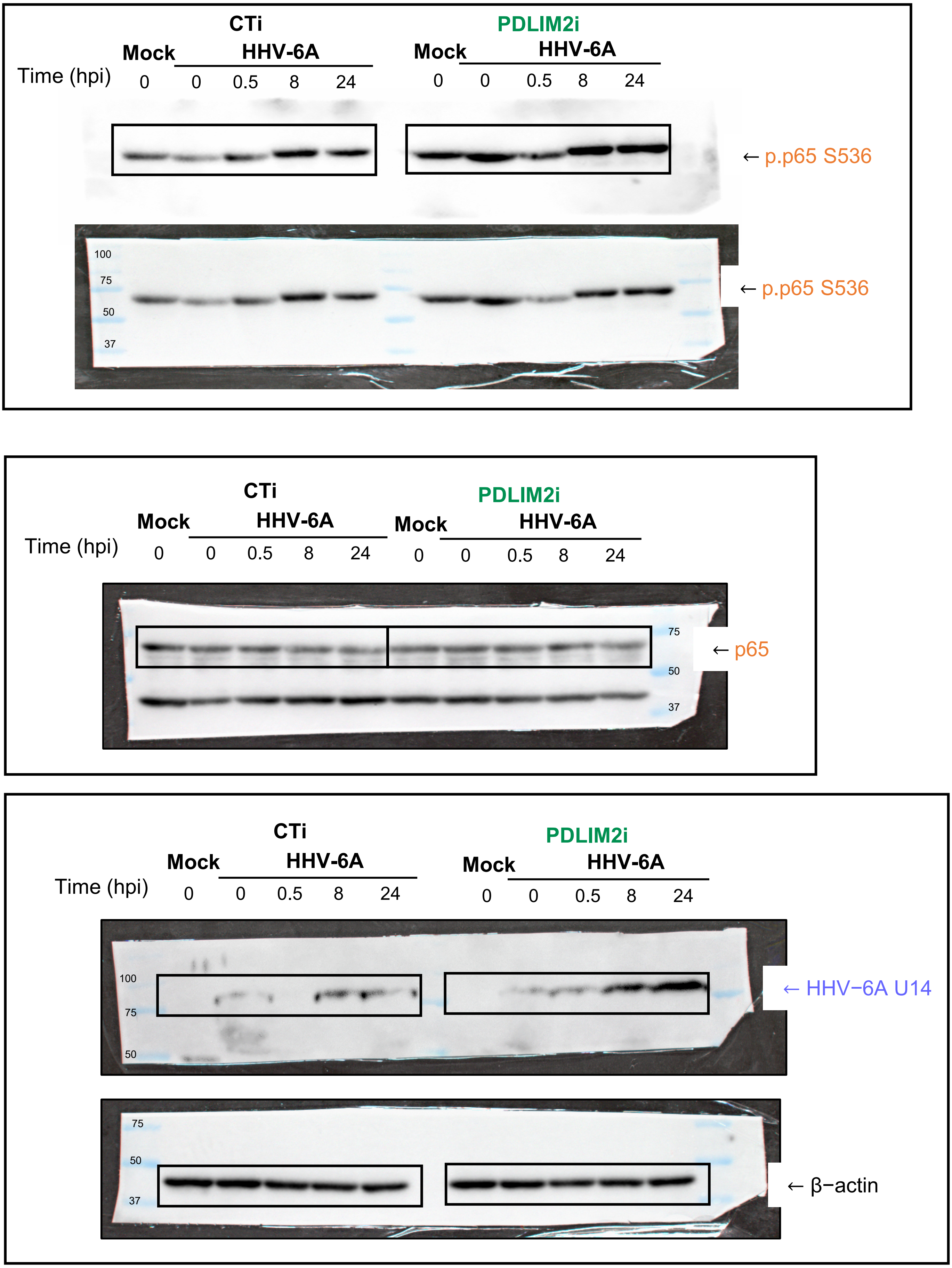
CTi
PDLIM2i
Mock
HHV-6A
Time (hpi)
0
0
0.5
8
24
← p.p65 S536
100
75
50
37
← p.p65 S536
CTi
PDLIM2i
Mock
HHV-6A
Time (hpi)
0
0
0.5
8
24
75
50
37
← p65
CTi
PDLIM2i
Mock
HHV-6A
Time (hpi)
0
0
0.5
8
24
100
75
50
← HHV-6A U14
75
50
37
← β−actin

Figure 4A

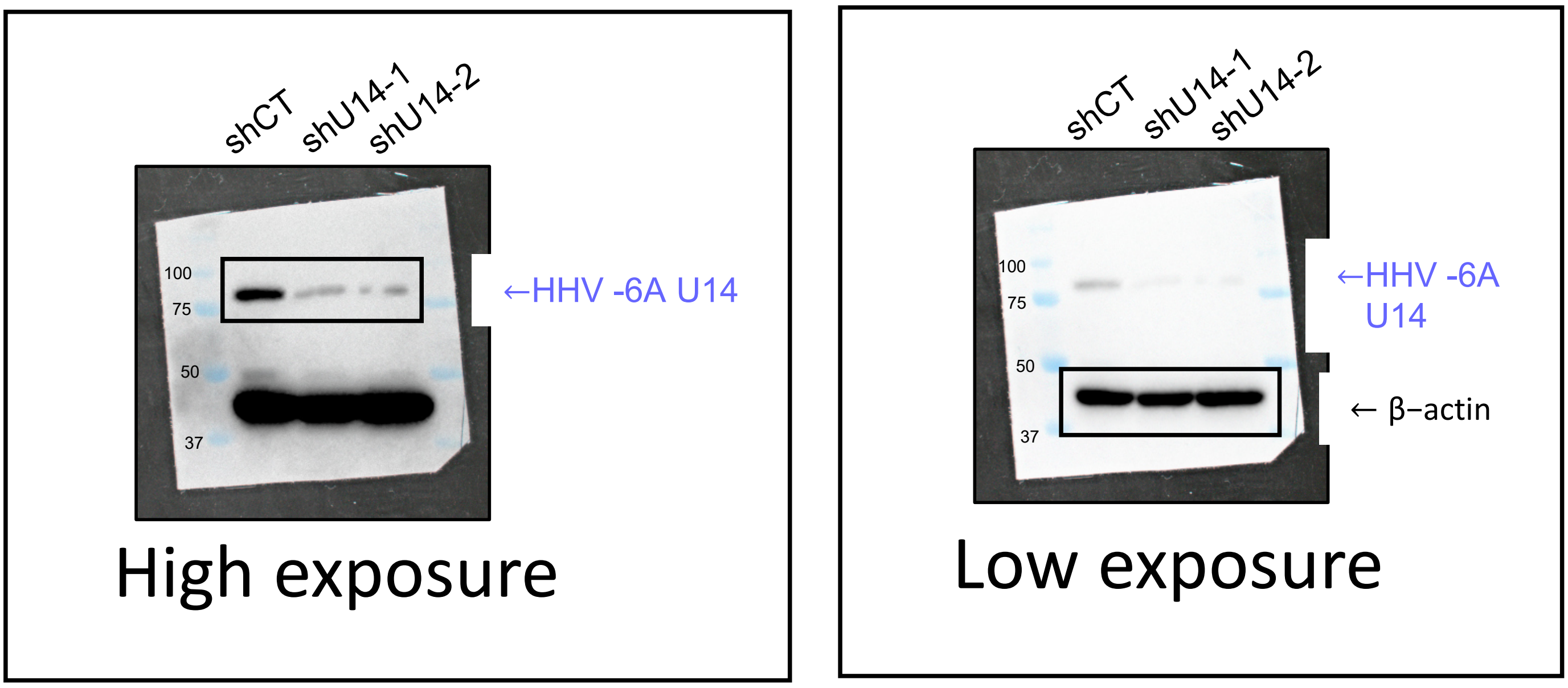

shCT
shU14-1
shU14-2
100
75
50
37
←HHV -6A U14
High exposure
shCT
shU14-1
shU14-2
100
75
50
37
←HHV -6A
U14
← β–actin
Low exposure

Figure 6A

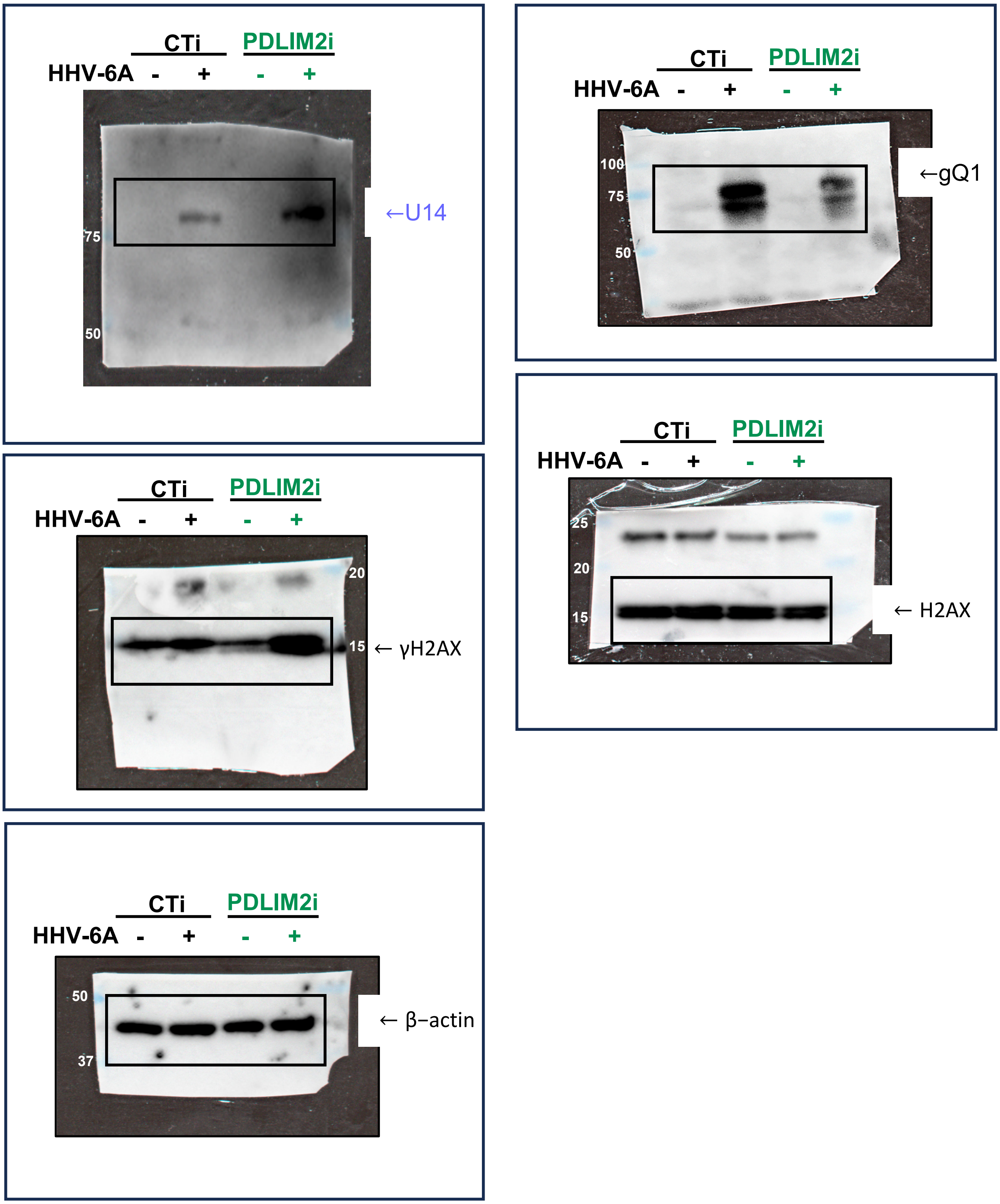
CTi
PDLIM2i
HHV-6A - + - +
75
50
←U14
100
←gQ1
20
15
← γH2AX
25
← H2AX
37
← β–actin

Supplemental Figure S1A

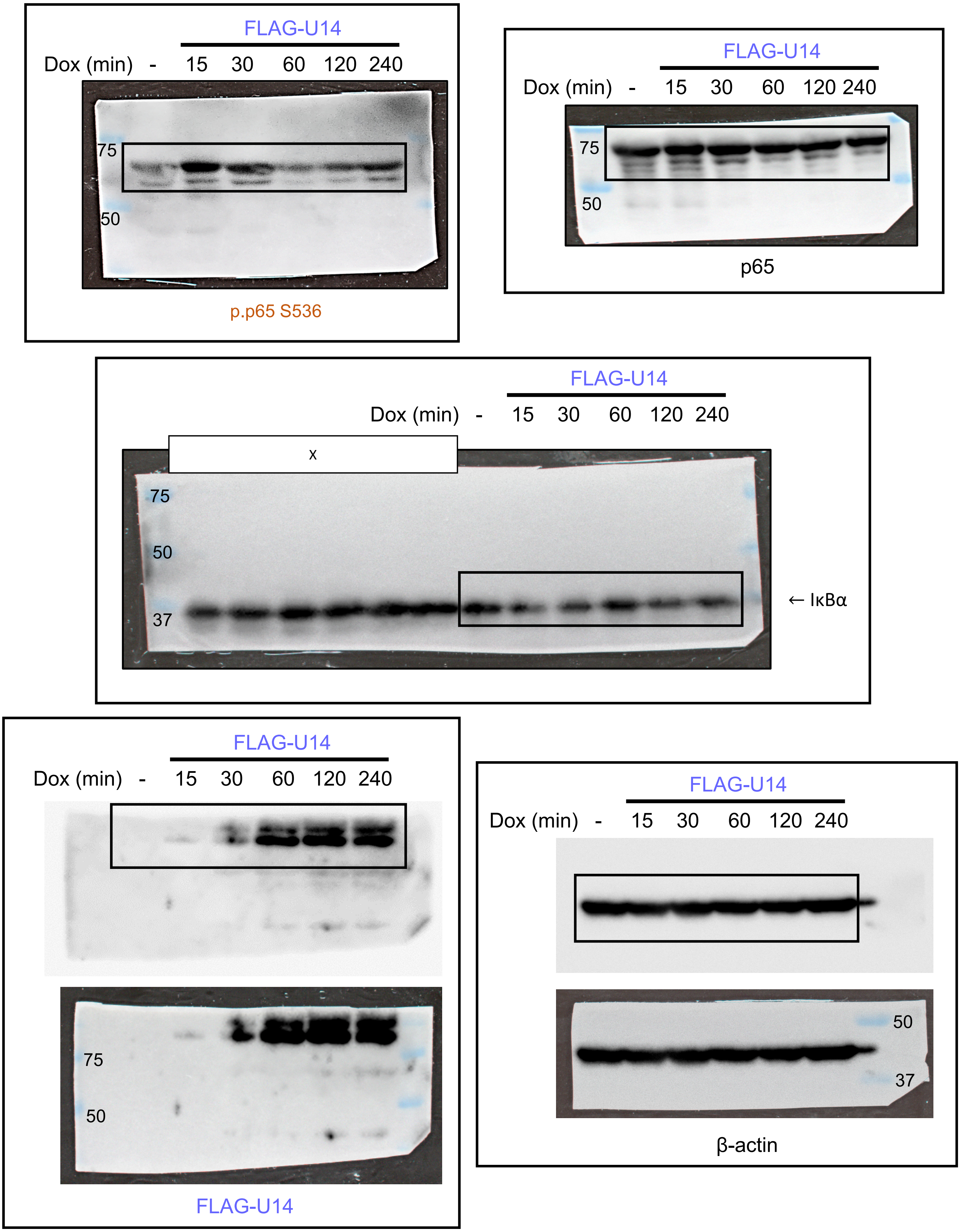

Supplemental Figure S1B

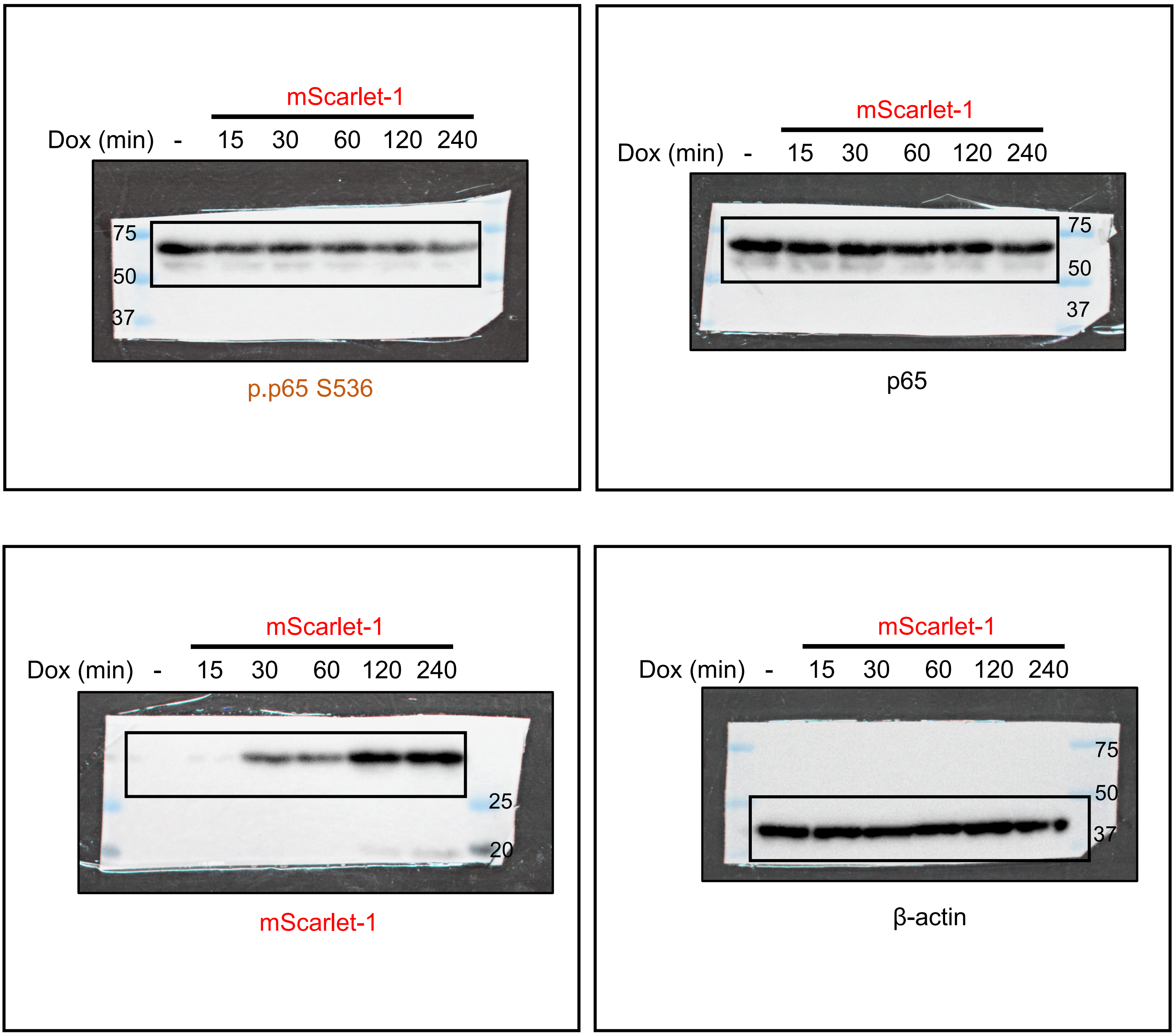

Supplemental Figure S1C

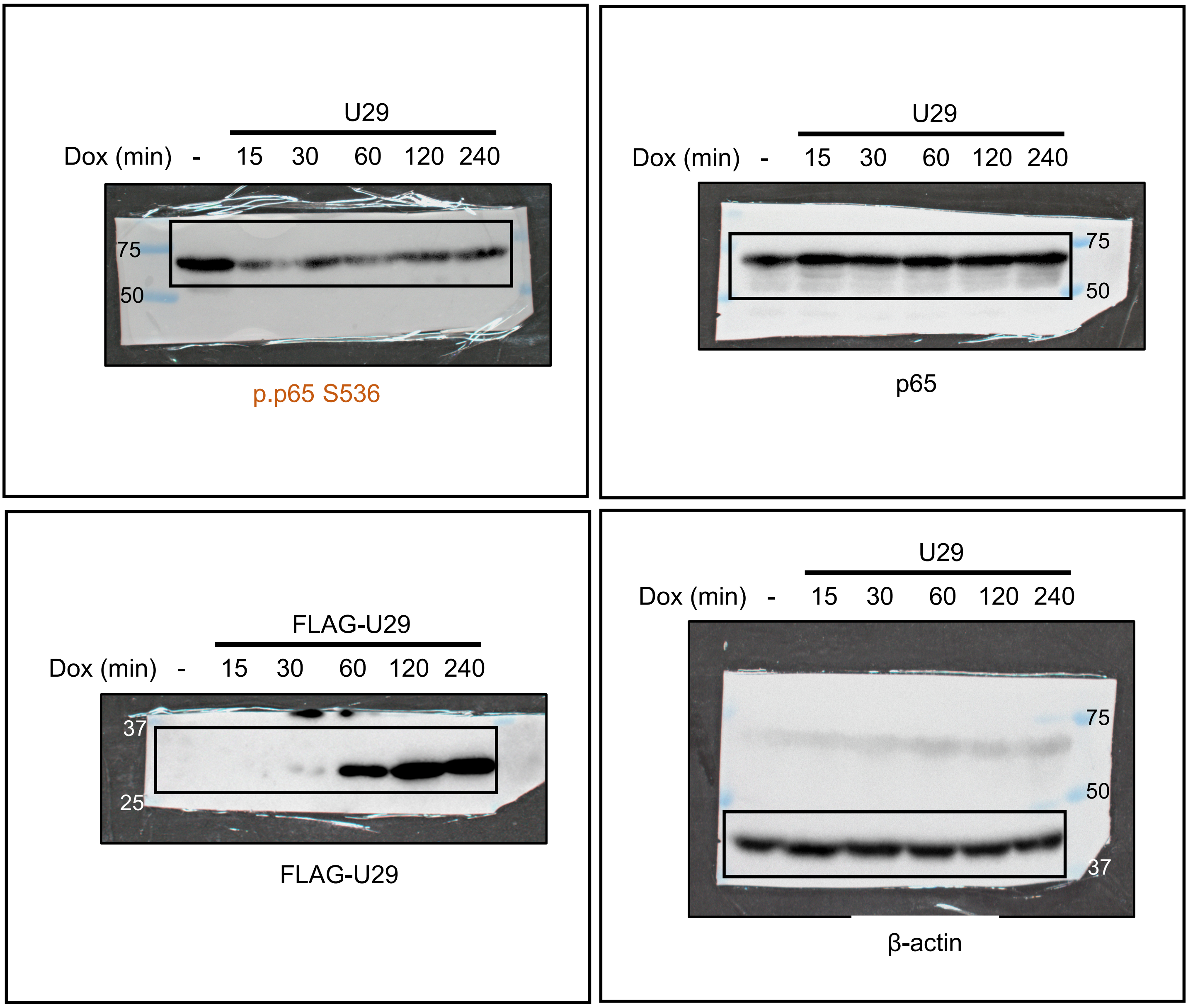

Supplemental Figure S4A

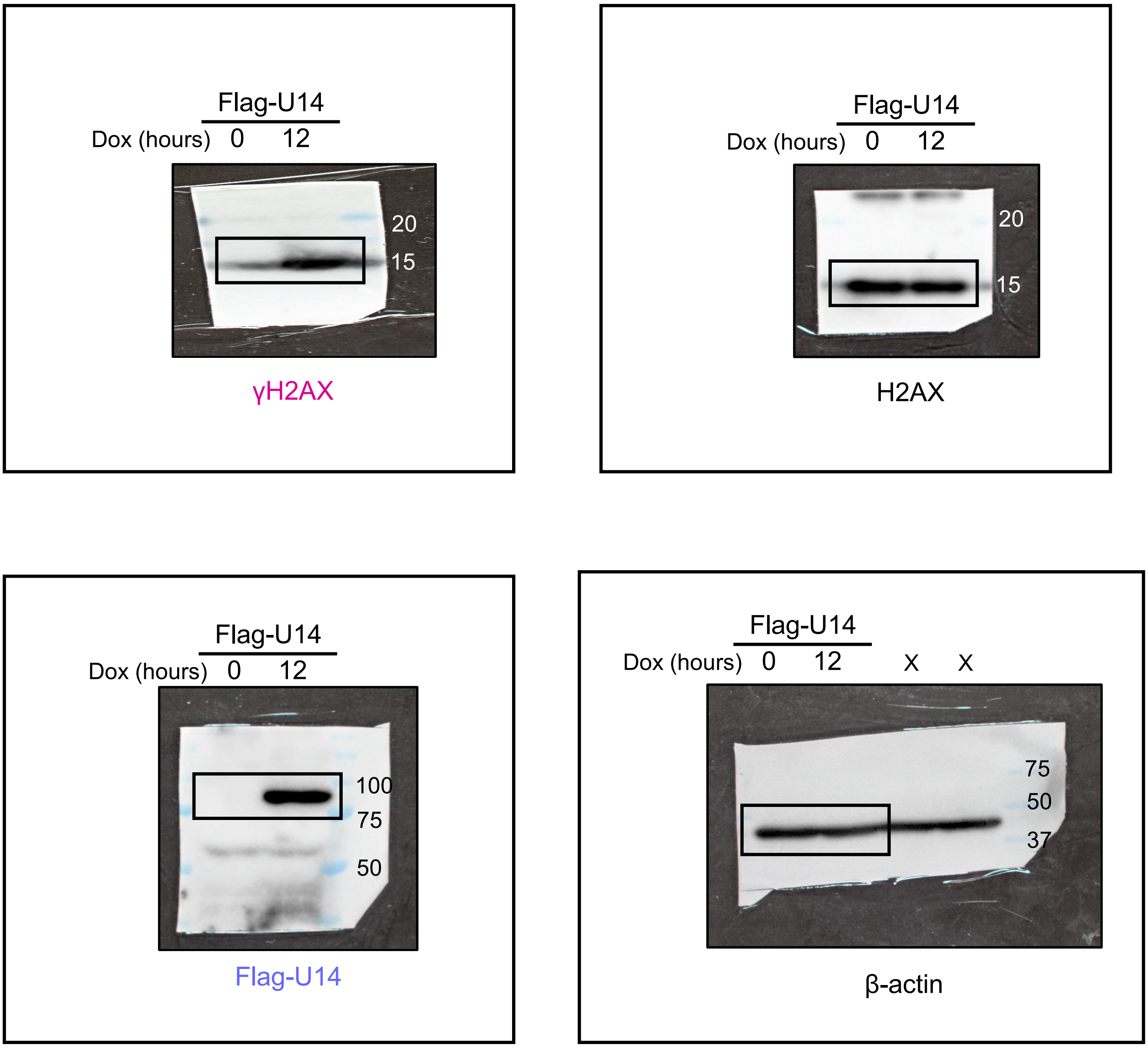

Supplemental Figure S6A

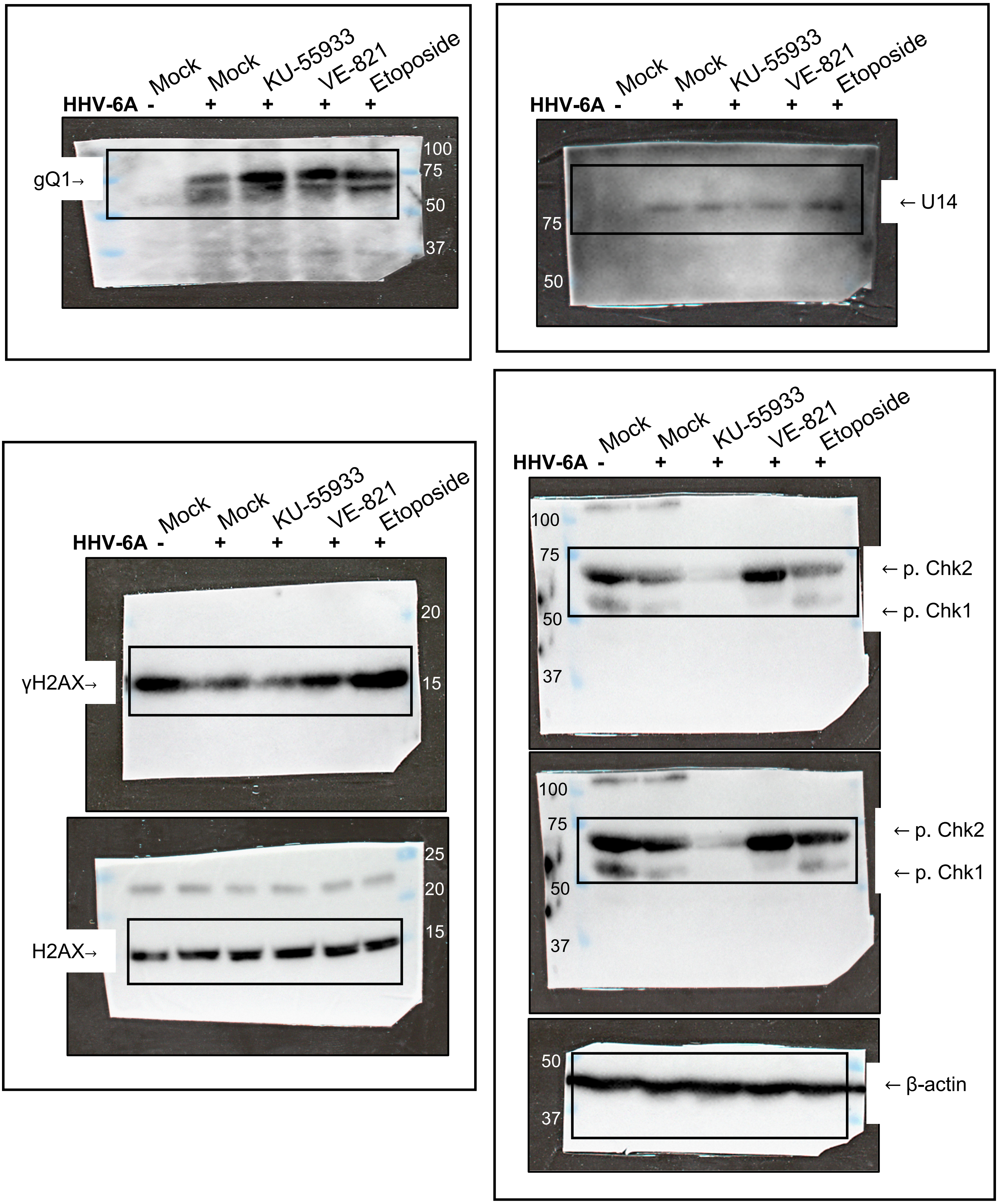

Supplement: Supplementary file 7 [file ppat.1014576.s007.pdf]
